# The combined impact of chemical and non-chemical stressors on adverse health outcomes: an overview of reviews from the epidemiological literature and experimental animal studies

**DOI:** 10.1038/s41370-026-00862-x

**Published:** 2026-06-30

**Authors:** Jessica Trowbridge, Emily Lasher, Xing Gao, Nicholas Chartres, Rashmi Joglekar, Rachel Morello-Frosch, Mary A. Fox, Devon Payne-Sturges, Jessie P. Buckley, Tracey J. Woodruff

**Affiliations:** 1 Program on Reproductive Health and the Environment, Department of Obstetrics, Gynecology, and Reproductive Sciences, University of California, San Francisco, CA USA; 2 School of Pharmacy, Faculty of Medicine and Health, The University of Sydney, Sydney, NSW Australia; 3 Department of Environmental Science and Policy Management and School of Public Health, University of California, Berkeley. Dr. Morello-Frosch stepped off the project as of May 1, Berkeley, CA USA; 4 Risk Sciences and Public Policy Institute, Department of Health Policy and Management, Johns Hopkins Bloomberg School of Public Health, Baltimore, MD USA; 5 Department of Environmental Health Sciences, University of Michigan School of Public Health, Ann Arbor, MI USA; 6 Gillings School of Global Public Health, Department of Epidemiology, University of North Carolina at Chapel Hill, Chapel Hill, NC USA; 7 Department of Epidemiology and Population Health & Woods Institute for the Environment, Stanford University, Stanford, CA USA

**Keywords:** systematic review, overview of reviews, non-chemical stressors, cumulative risk

## Abstract

**Background:**

Assessing environmental chemical health risks requires accounting for a range of factors that influence risk, including exposure to multiple chemicals, biological factors, and non-chemical stressors, such as socioeconomic status, access to health care, and discrimination. Exposure to non-chemical stressors can interact with or modify the effects of chemical exposures on adverse health outcomes, making them a critical component of cumulative risk assessment. However, incorporating this research into evidence-based decision-making, including cumulative risk assessment approaches, requires a comprehensive evaluation of the literature to understand the magnitude of non-chemical stressors' impact on the exposure-outcome relationship.

**Objective:**

To conduct an overview of reviews to identify, evaluate, and summarize the body of evidence on this topic in published systematic and scoping reviews.

**Methods:**

We pre-published our study protocol, conducted a systematic literature search, and we reviewed and extracted data from eligible studies, using the Navigation Guide Methodology for Systematic Review. We adapted and used the AMSTAR 2.0 tool to evaluate and rate the quality of environmental health studies.

**Results:**

We identified 13 systematic and five scoping reviews that met our pre-defined eligibility criteria. All reviews evaluated human epidemiologic evidence, and one evaluated human and animal evidence. Three reviews were of moderate quality, four reviews were of low quality, and 11 reviews were critically low. We summarized the findings from reviews rated as low or moderate quality (*n* = 7). The most common exposure was air pollution. Studies described a broad range of outcomes and used varied definitions and conceptualizations of non-chemical stressors, including using race ethnicity as an indicator of social stress or socioeconomic status (SES) or using direct and indirect measures of SES, such as health insurance status and median household income. Two reviews with certainty assessments found an increased association with adverse health outcomes when environmental chemical and non-chemical stressors were considered together versus individually.

**Impact:**

- Current risk assessment methods fail to account for the combined effect of chemical and non-chemical stressors. Consolidating systematic reviews on the influence of extrinsic non-chemical stressors can facilitate the development of risk assessment methods that account for their impact.

## Introduction

Exposure to environmental chemicals, such as air pollution, industrial pollution, metals, among others, can increase the risk of multiple adverse health outcomes, including respiratory and cardiovascular illness, decreased fertility, and infant and child developmental problems [1–3]. Communities of color and low-income communities face a disproportionate burden of exposure to environmental chemicals from sources, such as air pollution, industrial sites, poor drinking water quality, and toxic consumer products, among others [4–7]. These higher exposures contribute, in part, to racial and economic health inequities in the United States [8].

In addition to higher exposures that can increase health risks, the adverse impact of chemical exposure on health outcomes can interact with or be modified by exposures to non-chemical stressors among individuals and communities [9]. In this paper we define non-chemical stressors as extrinsic factors that can influence a persons or communities susceptibility to an environmental exposure and includes food insecurity, geography (e.g., urban vs rural), poverty, socioeconomic status, lack of access to healthcare, racism, discrimination, poor housing quality, and exposure to violence among others, or protective factors, such as access to greenspace, among others [10]. Environmental chemicals and non-chemical stressors can interact to increase the risk of adverse health outcomes compared to the chemicals or non-chemical stressors alone [11–13]. Low-income communities and communities of color often face what is considered the “double jeopardy” of exposure to chemical- and non-chemical stressors, experiencing disproportionately higher exposures to both [14, 15]. Non-chemical stressors can enhance susceptibility to the adverse health effects of chemical stressor exposure [16, 17]. Thus, a failure to consider the combined impact of non-chemical stressors when evaluating chemical exposures and their associated adverse health outcomes results in an incomplete understanding of the exposure-outcome relationship, potentially underestimating risk to human health, particularly for susceptible communities [10, 18, 19].

Current risk assessment approaches for identifying and quantifying the level of risk from chemical exposures typically account for single-chemical and single-source exposures, often failing to consider the additional risk from non-chemical stressors [19]. In addition, there is a lack of consensus on which non-chemical stressors are most important to consider. Currently, there is a lack of tools or frameworks to rigorously and consistently incorporate non-chemical stressors into risk assessment and risk management strategies [20, 21].

The National Academies of Science, Engineering, and Medicine has recommended the use of systematic reviews for evaluating environmental health-related evidence [22]. Systematic reviews are a method for identifying and evaluating all relevant studies in a transparent manner using a pre-established protocol. Failure to use systematic review methods can lead to the exclusion of relevant studies and to an underestimation of the risk of environmental exposures. Prior systematic reviews have described the combined impact of non-chemical stressors and chemical exposures for various adverse health outcomes [23, 24]. However, no comprehensive study has identified all relevant systematic reviews on these topics and evaluated their quality. Such an overview will both inform strategies for incorporating non-chemical stressors in cumulative risk assessment (CRA) and provide an understanding of the current state of knowledge to guide future research in this area. Thus, our goal was to conduct an overview of reviews to comprehensively identify, evaluate, and synthesize relevant systematic and scoping reviews using best practices that can inform CRA [25, 26]. The objective of this overview of reviews is to consolidate the available evidence of the influence of extrinsic non-chemical stressors on the relationship between environmental chemical exposures and adverse health effects in scoping and systematic reviews and to develop recommendations for accounting for the impact of non-chemical stressors in risk assessment at regulatory agencies like the United States Environmental Protection Agency. We aimed to identify the existing literature, evaluate the quality of existing systematic reviews and scoping reviews on this topic, and determine whether the evidence presented was of sufficient quality to answer our study question: How do non-chemical stressors interact with or modify the relationship between chemical exposures and adverse health outcomes? In cases where the evidence or quality was lacking, this overview would allow us to identify what future research was needed to improve the evidence base.

## Methods

To evaluate the existing literature on the extent to which non-chemical stressors can interact with or modify the chemical exposure-outcome relationship, we conducted an overview of reviews (hereafter referred to as “overview”). Systematic reviews attempt to “identify, appraise, and synthesize all the empirical evidence that meets pre-specified eligibility criteria to answer a specific research question” [27]. Overviews, also referred to as “umbrella reviews,” “review of reviews,” and “meta-reviews,” follow the steps of a full systematic review; however, instead of evaluating primary studies, overviews evaluate and synthesize systematic reviews [25]. This overview adheres to the PRIOR checklist for overview of reviews (Table S1) [28].

### Protocol

The methods of our overview were informed by Cochrane’s Overview of Reviews guidance [25] and the Navigation Guide [26]. The overview protocol was prospectively registered on Open Science Framework (https://osf.io/m7936). There were minor deviations from the protocol (see supplemental materials for “Deviations from the Protocol”).

### Study question

What is the interaction or modification effect of non-chemical stressors on the exposure-outcome relationship?

### PECO statement

To evaluate the impact of non-chemical stressors on the relationship between environmental chemical exposures and adverse health outcomes, we included studies that met our PECO (**P**opulation, **E**xposure, **C**omparator, and **O**utcome) statement and pre-defined eligibility criteria.

**P**opulation: Systematic reviews (as defined by our specific criteria^1^ using modified PRISMA guidelines [30] (Table S2)) and scoping reviews (as defined by study authors) on animal and human epidemiology studies, with or without meta-analyses.

Exposure: environmental agents (chemicals and environmental pollutants, such as metals) and extrinsic susceptibility factors, a subset of non-chemical stressors that can modify or moderate the exposure-outcome relationship. Extrinsic susceptibility factors included indicators for allostatic load, discrimination/racism, education, food insecurity, geography, protective factors, socioeconomic status (SES) or socioeconomic position (SEP), sleep quality, general stress, and work-related stress. Stressors included individual-level factors as well as aggregate or community-level factors. Exposures could occur during any life stage and for any duration.

**C**omparator: high-chemical and high-stressor exposure vs. low-chemical and low-stressor exposures as well as high-exposure/low-stressor and low-stressor/high-chemical exposure categories as intermediate stressor levels vs low-stressor, if possible, with the data presented in the included reviews. The considered stressors will include individual-level factors, as well as aggregate or community-level factors, and will be defined by the available literature.

**O**utcomes: any adverse health outcome. We used the Environmental Protection Agency’s Integrated Risk Information System (IRIS) definition, which is “a biochemical change, functional impairment, or pathologic lesion that affects the performance of the whole organism or reduces an organism’s ability to respond to an additional environmental challenge” [31].

### Eligibility criteria

In addition to fitting our PECO statement, we included studies that met the following criteria:

- Full-text systematic reviews or scoping review articles
- Reviews published at any time up to the search date of Jan 31, 2024

We excluded:

- Any review that is not systematic or scoping, including narrative reviews, based upon the definition provided by the study authors and our modified PRISMA guidelines [30], assessed during our full-text screen (Table S2).
- Conference abstracts with no available full systematic or scoping review.
- Non-full-text articles, or if the full text was unavailable.
- Systematic or scoping reviews in a language other than English (and not available in English). Non-English studies were captured in the search and identified as potentially relevant supplemental materials but excluded in full-text review.
- Primary studies (including studies that used meta-analyses on individual participant data or cohorts to obtain their effect estimate).
- Systematic or scoping reviews that did not have a qualitative or quantitative relevant measure of at least one chemical and one non-chemical stressor.
- Systematic or scoping reviews that evaluated pharmaceuticals or nutritional supplements.

### Search strategy and information sources

We collaborated with an information specialist (JF) and consulted with topic area experts to develop the search strategy, building upon searches that were conducted previously [23, 32]. We searched PubMed and Web of Science to capture peer-reviewed systematic reviews and scoping reviews through January 31, 2024. We did not limit the search by language or publication date. The full search string can be found in Table S3. We did not search the reference lists of included studies.

### Data management

We de-duplicated the studies using EndNote [33], then uploaded the references into a literature review web tool (Litstream^TM^) for screening and data extraction [34]. Each article was assigned a unique identification number in Litstream that was utilized for the entirety of the project.

### Screening and study selection

Prior to screening, the study team pilot-tested the title/abstract and full-text forms with 20 and three studies per screener, respectively, to ensure standard interpretation of criteria [35]. Study screening at the title/abstract and full-text level was conducted in duplicate; two screeners (JT, XG, OS, RS, SF, RJ, ShS, or SwS) screened each reference and decided whether it met our inclusion criteria [35]. Any conflicts were resolved by a third investigator or screener (RJ and CR) to attain 100% agreement for all included studies.

### Data extraction

Prior to data extraction, the study team pilot-tested the extraction form with two studies per extractor to ensure consistency [36]. The following data were extracted from the included reviews:

- study author
- publication year
- corresponding author country
- review objective
- PECO statement
- review methods
- exposures (chemical and non-chemical)
- outcomes evaluated
- review results (relative effects or narrative results), including findings related to joint exposure to chemical and non-chemical stressors
- certainty assessments
- overall conclusions (verbatim)
- author affiliations, and
- funding and conflict of interest statements*

*We used the funding statement to assess if the review had industry sponsorship from the companies that manufacture, market, and sell the chemicals under evaluation, or, in the case of reviews on air pollution, fossil fuel companies or the automobile industry. Industry sponsorship is important to report as a potential source of bias.

We extracted the protocol registration number/web link and contacted the authors when they indicated that they pre-published a protocol, but it was not available online [36, 37]. We emailed one author (Vesterinen) when the protocol was listed as being published, but was not available online, and received the protocol via email. We did not identify missing data in other studies, requiring us to reach out to other authors. One reviewer conducted the primary extraction (XG or OS) and a second reviewer verified the extracted data for accuracy and completeness (JT or CR) [36].

### Quality assessment

We used a modified version of AMSTAR 2.0 (A MeaSurement Tool to Assess Systematic Reviews) to evaluate the quality of the included systematic and scoping reviews (Table S4) [38]. We modified the tool with expert input (NC, TW) to better reflect the evidence base available in the environmental health literature [39]. For instance, we revised criterion 9 (Did the authors use a satisfactory technique for assessing the risk of bias (RoB) in individual studies that were included in the review?) to be more reflective of the tools used in environmental health to evaluate study risk of bias, such as explicitly conducting a qualitative-domain-based tool for RoB. The tool notes that amendments are justifiable and that the criteria are provided as suggestions [38]. Previous evaluations of systematic reviews, including by the National Academies of Sciences, have made minor adaptations to the tool [40].

Each review was evaluated by two investigators (JT, EL, conflicts resolved by consensus), for all sixteen domains, with special consideration given to the six “critical” criteria which include: protocol was registered before the commencement of the review (AMSTAR 2); adequacy of the literature search (AMSTAR 4); justification for excluding individual studies (AMSTAR 7); risk of bias from individual studies being included in the review (AMSTAR 9); appropriateness of the methods (AMSTAR 11); consideration of the risk of bias when interpreting the results of the review (AMSTAR 13).

Scoping reviews were evaluated for the same criteria; however, we did not score them for criteria 9 and 13, as scoping reviews do not conduct risk of bias assessments. Scoping reviews were also exempt from AMSTAR 14 (discussion of heterogeneity).

Eligible systematic and scoping reviews were rated as:

High – the review has no or one non-critical weakness and provides an accurate and comprehensive summary of the results of the available studies that address the question of interest.

Moderate – the review has at least one “partial yes” in a critical component and/or more than one non-critical weakness, and may provide an accurate summary of the results of the available studies that were included in the review.

Low – the review has one critical flaw, with or without non-critical weaknesses, and may not provide an accurate and comprehensive summary of the available studies that address the question of interest.

Critically low – the review has more than one critical flaw, with or without non-critical weaknesses, and should not be relied on to provide an accurate and comprehensive summary of the available studies.

Reviews of high, moderate, or low quality from the AMSTAR tool and those that conducted a certainty of the evidence assessment were used to draw conclusions about the influence of non-chemical stressors on the relationship between environmental chemical exposures and adverse health outcomes. We excluded reviews of critically low quality from data synthesis and analysis.

### Certainty of evidence

Among the studies that were high, moderate or low quality from the AMSTAR tool, we relied on the review authors’ assessment of the certainty of the body of evidence (e.g., GRADE (the grading of recommendations assessment, development, and evaluation). GRADE is a systematic approach for rating the quality of a body of evidence in systematic reviews, first considering the study design and then upgrading or downgrading the quality of evidence based on various factors. Factors that can reduce the quality of evidence include risk of bias, imprecision, inconsistency, indirectness, and publication bias, while factors that can increase the quality of evidence include large magnitude of effect, dose-response, and confounding that minimizes effect [41]. The quality of the body of evidence is rated on a scale of high, moderate, low, or very low, with a rating of high indicating that we are very confident that the true effect is similar to the estimated effect, while a rating of very low indicates that we have very little confidence in the effect estimate [41]. We did not conduct certainty assessments using GRADE or other methods [e.g., the NTP/OHAT (National Toxicology Program Office of Health Assessment and Translation approach)] for reviews not reporting them. First, we included studies that were scored as low, moderate, or high quality using the AMSTAR tool; very low-quality studies were excluded. Then, we prioritized the included systematic reviews that conducted certainty assessments, and on which we can rely to draw conclusions on the relationship between chemical and non-chemical stressors on the outcome (Tier 1). For reviews without a certainty assessment (Tier 2), we cannot rely on their findings to draw conclusions on the effect of chemical and non-chemical stressors on the outcome.

### Primary study overlap

Overviews require an assessment of primary study overlap, as reviews on the same topic may include the same primary studies, introducing bias [25, 42, 43]. To identify overlap, we first mapped the included studies’ exposures and outcomes. In the case that multiple reviews evaluated similar exposures and outcomes, we utilized the established Graphical Representation of Overlap for OverViEs (GROOVE) tool to measure overlap [44]. We calculated the corrected covered area (CCA) and considered less than 5% slight overlap, 5–10% moderate overlap, 10–15% high overlap, and more than 15% very high overlap. As the overview aims to describe the available evidence, we presented the results from all studies regardless of overlap [25].

### Data analysis

We extracted and reported the populations, exposures, non-chemical stressors evaluated, and outcomes measured in the eligible systematic and scoping reviews. We also included narratively reported study results and the results from any meta-analyses conducted. We extracted and reported effect estimates, 95% confidence intervals, and measures of heterogeneity if meta-analyses were conducted. We extracted results to compare high- and low-stressor conditions when available. When available, we included the quantitative results of meta-analyzed data and the authors’ certainty of the evidence assessments. We did not reanalyze any data from the eligible systematic or scoping reviews.

## Results

### Study selection

**Fig. 1:**
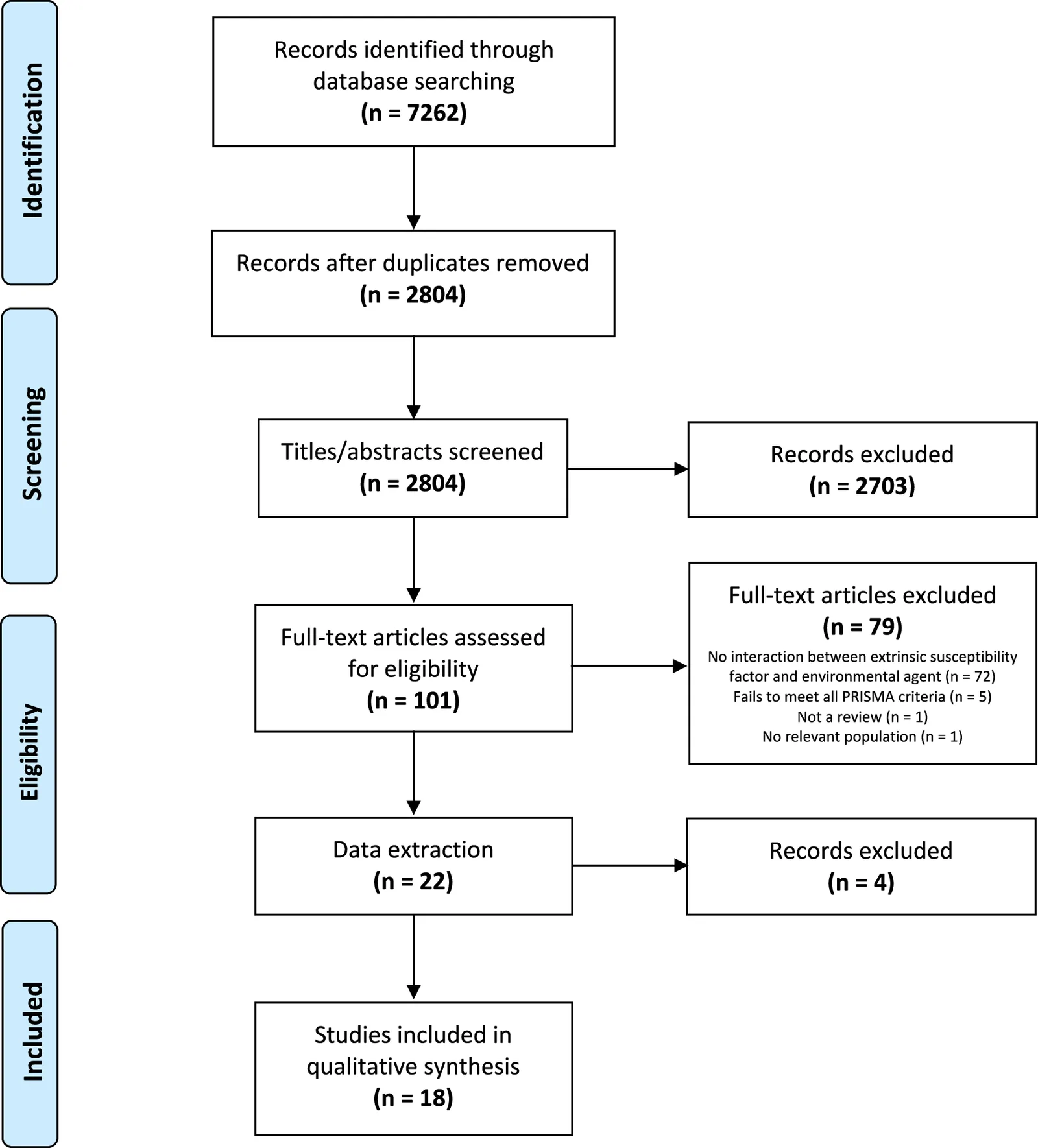
Flowchart of the search and selection process of systematic and scoping reviews that met inclusion criteria.

Our literature search initially identified 7262 records. After deduplication, the final number of returns was 2804 (Fig. 1). We excluded a total of 2703 records after reviewing the titles and abstracts. We further excluded 79 of the remaining 101 records after reviewing the full text. A complete list of the citations and justification for exclusion at full text can be found in Table S5. Ultimately, we extracted data from 22 reviews; however, after extraction, we identified four additional reviews that did not meet our inclusion criteria (Table S6).

### Description of included reviews

Of the 18 reviews, 13 were systematic reviews and five were scoping reviews (details in Table 1). The reviews were published between 2010 and 2022 by corresponding authors with affiliations in the United States (*n* = 11), China (*n* = 2), Canada (*n* = 2), Mexico (*n* = 1), Germany (*n* = 1), and Israel (*n* = 1). Four reviews pre-published a protocol detailing the review methods. Each review contained between 10 and 281 primary studies. Six reviews conducted a risk of bias assessment, using a variety of tools. These included tools that use a domain-based approach, such as the National Toxicology Program’s Office of Health Assessment and Translation (NTP OHAT) tool (*n* = 2) and the Navigation Guide (*n* = 2). Others used a quantitative or scoring system that is not empirically supported or recommended by leading authoritative bodies [45], such as the Newcastle Ottawa Scale (NOS) (*n* = 2), ROBINS-E (*n* = 1), and the Joanna Briggs Institute Meta-analysis of Statistics Assessment and Review Instrument (*n* = 1). Two reviews conducted a certainty assessment using the GRADE approach. Two reviews were sponsored by the Health Effects Institute, which receives funding from the automobile industry. Six reviews did not disclose whether the review had received funding or if the authors had a financial conflict of interest. Ten reviews did not have a conflict of interest. The majority of reviews (17/18) evaluated studies relating to air pollution, though three reviews investigated other chemical exposures, such as metals, pesticides, and phthalates. One review explored proximity to oil and gas development. The reviews assessed a myriad of health outcomes, including mortality, injury, obesity, cancer, and endpoints related to the reproductive, respiratory, cardiovascular, nervous, endocrine, and immune systems. Reviews also considered a wide range of non-chemical stressors, such as race and ethnicity, socioeconomic status, country development status, and neighborhood characteristics. A total of five reviews conducted a meta-analysis accounting for both environmental exposures and non-chemical stressors.

**Table 1:** Study characteristics of systematic and scoping reviews included in the overview.

Review	Type	Country	Search dates	Protocol	number of studies	number of studies evaluating chemical and non-chemical stressors	RoB tool	Certainty assessment	Environmental stressors	Outcomes	Non-chemical stressors	Meta-analysis with chemical and non-chemical stressors	Author COI	Industry Sponsorship
**Bell et al., 2013** **[**46]	SR	USA	1996 - 26 July 2012	None	63	9	None	No	Air pollution (PM_2.5_ and PM_10_)	Mortality, hospital admissions, emergency room visits	Race/ethnicity, SES indicators (education, income, poverty, employment status)	No	No	Yes
**Bell et al., 2014** **[**47]	SR	USA	1988 - 2013	None	51	17	None	No	Air pollution (O_3_)	Mortality (total, cardiovascular disease, respiratory), hospitalization (cardiovascular disease, respiratory, asthma)	Race/ethnicity, SES indicators (education, income, employment/occupation, poverty), air conditioning	No	No	Yes
**Deziel et al., 2020** **[**48]	Scoping	Israel	From inception to 15 Aug 2019	None	29	3	None	No	Unconventional oil and gas development	Adverse pregnancy outcomes, hospitalization, asthma exacerbation, cardiovascular disease, cancer, sexually transmitted infections, injuries	Family income, urban/rural, environmental law violations	No	No	No
**Fuller et al., 2017** **[**49]	SR	USA	1 Jan 2005 – 31 Dec 2016	None	30	30	None	No	Air pollution (O_3_, PM_10_, PM_2.5_, NO_2_, CO, SO_2_, TSP)	Cardiovascular disease outcomes	Material resources at individual and community level (education, income, employment, deprivation index); Psychosocial stress	No	No	No
**Heo et al., 2019** **[**50]	SR	USA	1 Jan 2000 – 31 July 2019	None	45	31	None	No	Air pollution (PM_2.5_, PM_10_, smoke, coarse PM, NO_x_, TSP, traffic volume)	Birth outcomes (birthweight, preterm birth, small-for-gestational age, gestational diabetes)	Race/ethnicity, educational attainment, income, area-level SES	No	Unclear	No
**Li et al., 2022** **[**51]	SR	China	From inception to 17 Dec 2020	PROSPERO CRD42021250279	35	0*	NOS, JBI	No	Air pollution (PM_10_, NO_2_, SO_2_, O_3_, CO)	Allergic rhinitis	Developing country status, geographic area	Yes	No	No
**Lin et al., 2022** **[**52]	SR	China	From inception to Jan 2022	None	30	0*	NTP-OHAT	Yes	Air pollution (PM_2.5_, PM_10_, “chemicals”, SO_2_, NO_2_, CO, traffic density, coarse PM)	Obesity	Developing country status	Yes	No	No
**Munoz-Pizza et al., 2020** **[**53]	Scoping	Mexico	Jan 2000 – Dec 2017	None	17	17	None	No	Air pollution (O_3_, SO_2_, NO_2_, PM_10_, TSP, NO_x_)	Children’s respiratory health	Household income, health insurance status, parental education, race, poverty, unemployment, migratory status, psychosocial stress, community SES	No	No	No
**Namin et al., 2021** **[**54]	Scoping	USA	1 Jan 1990 – 1 Aug 2020	None	281	108	None	No	Air pollution, contaminated water, residential radon, undesirable land use	Cancer	Residential history/mobility (social environment factors, built environment, individual factors)	No	Unclear	No
**Payne-Sturges et al., 2023** **[**23]	Scoping	USA	From inception to 18 Nov 2022	None	212	74	None	No	Air pollution, lead, mercury, organophosphate pesticides, phthalates, polybrominated diphenyl ethers, polychlorinated biphenyls	Child neurodevelopment (IQ, FSIQ, etc.)	Race/ethnicity, geography, SES, social adversity indices, language, and Home Observation for Measurement of the Environment (HOME) Inventory scores	No	No	No
**Rodriguez-Villamizar et al., 2016** **[**55]	SR	Canada	1950 – 1 July 2015	PROSPERO CRD42015022166	10	10	NTP-OHAT	No	Air pollution (O_3_, CO, NO_2_, PM_10_, SO_2_)	Asthma	Area-level socioeconomic position (income, education, occupation) based on census data; Individual-level socioeconomic position	No	Unclear	No
**Ruiz Idel et al., 2016** **[**56]	Scoping	USA	2003 - 2013	None	258	21	None	No	Arsenic, fluoride, lead, manganese, indoor NO_2_, polycyclic aromatic hydrocarbons, polybrominated diphenyl ethers, phthalates, pesticides	Children’s cognitive health (neurodevelopment scores)	Maternal education, SES, race/ethnicity	No	No	No
**Rugel and Brauer, 2020** **[**57]	SR	Canada	2003 – Nov 2019	PROSPERO CRD42018106050	51	20	ROBINS-E	Yes	Traffic-related air pollution	Mortality, cardiovascular disease, chronic respiratory disease, allergy, type II diabetes, reproductive outcomes	Noise, natural space, neighborhood walkability	No	No	No
**Son et al., 2021** **[**58]	SR	USA	From inception to 30 Nov 2020	None	20	20	None	No	Air pollution	Mortality, hospital admissions, cardiovascular disease, respiratory outcomes	Greenness (e.g., normalized difference vegetation index, land use)	No	Unclear	No
**Thayamballi et al., 2021** **[**59]	SR	USA	From inception to 30 June 2018	None	64	64	None	No	Air pollution (PM_10_, PM_2.5_)	Birth outcomes (low birth weight, preterm birth, small-for-gestational age, stillbirth)	Race/ethnicity, maternal education	Yes	No	Unclear
**Vesterinen et al., 2017** **[**22]	SR**	USA	From inception to 29 May 2013	Yes***	69	69	Nav Guide	No	Smoking, alcohol, benzene, air pollution (proximity to traffic, O_3_, PM, NO_2_, SO_2_), aluminum, caffeine, diesel exhaust particulates, lead, methylmercury chloride, PFOS, sodium arsenate toluene, uranium	Human: birth outcomes (birthweight, small-for-gestational age), Developmental outcomes	Human: major life events, individual-level or place-based measures of material deprivation, poverty and other indicators of SES	Yes	No	No
											Animal: restraint, heat, nest material restriction, no light, restrain with bright light, various stressors			
										Animal: Pre- or post-natal offspring weight				
**Weinmayr et al., 2010** **[**60]	SR	Germany	1990 – 1 July 2008	None	77	50	None	No	Air pollution (PM_10_, NO_2_)	Asthmatic children’s respiratory health (respiratory symptoms, peak expiratory flow)	Urbanicity, geographic area	Yes	No	Unclear
**Yu et al., 2022** **[**61]	SR	USA	From inception to 1 May 2021	None	11	4	NOS/ Nav Guide	No	Air pollution (traffic NO_2_, CO, O_3_, PM), Mercury	Autism spectrum disorder	Education, neighborhood characteristics (deprivation, urbanicity, poverty rate), race	No	No	No

*RoB* Risk of Bias, *COI* Conflict of Interest, *SR* Systematic Review, *SES* socioeconomic status, *SEP* socioeconomic position, *NOS* Newcastle Ottawa Scale, *JBI* Joanna Briggs Institute Meta-analysis of Statistics Assessment and Review Instrument, *NTP-OHAT* National Toxicology Program Office of Health Assessment and Translation, *Nav Guide* Navigation Guide, *PM* Particulate Matter, *O3* ozone, *NO2* nitrogen dioxide, *CO* carbon monoxide, *SO2* sulfur dioxide, *TSP* total suspended particles, *NOx* nitrogen oxides, *FSIQ* full scale intelligence quotient, *PFOS* perfluorooctane sulfonate

Study Sponsorship & COI Coding

Study Sponsorship

• If there is no funding disclosure statement, it is “unclear”

• If there is one, and there is an industry sponsor, it is “yes”

• If there is no industry sponsor, it is “no”

Author COI

• If there is no author disclosure statement, then look at the author’s affiliations. If none of the authors are employed by an industry sponsor, it is “unclear.”

• If there is an author disclosure statement, and they declare they have no COI with industry, then look at the author’s affiliations. If none of the authors are employed by an industry sponsor, it is “no.”

• If there is an author disclosure statement, and they declare they have a COI with industry, or if ANY of the authors are employed by an industry sponsor, based on their affiliations then it is “yes.”

*Though the primary studies did not directly evaluate non-chemical stressors, the review authors conducted a meta-analysis comparing results by region and country SES.

**Vesterinen et al. 2017 reviewed human and animal evidence.

***Protocol was originally published online at http://www.dcn.ed.ac.uk/camarades/research.html#protocols but is no longer publicly accessible online. We contacted the authors and received the protocol by request.

### Quality assessment

We rated three reviews as “moderate” quality (Table 2) [23, 46, 47]. These studies had no critical weaknesses but had at least one “partial yes” in a critical component and/or multiple non-critical weaknesses. We rated four reviews as “low” quality, containing one critical weakness [24, 48–50]. We rated eleven reviews as “critically low” quality due to multiple critical flaws in their methodology [51–61]. We excluded critically low reviews from further analyses, leaving seven studies for further evaluation.

**Table 2:** Results of AMSTAR-2 assessment evaluating the quality of systematic and scoping reviews on joint effects of chemical and non-chemical stressors.

Review	AMSTAR-2 Criteria																Overall Quality
	1	2*	3	4*	5	6	7*	8	9*	10	11*	12	13*	14	15	16	
	PECO	Protocol	Study Designs	Search Strategy	Study Screening -Duplicate	Data Extraction - Duplicate	List of Excluded Studies	Description of Included Studies	RoB Tool	Primary Study COI	MA Methods	Impact of RoB on MA	RoB Discussion	Heterogeneity	Publication Bias	COI	
*Systematic Reviews*																	
Rodriguez-Villamizar et al., 2016	+	+	-	+	+	+	+	P	P	-	NA/M	NA/M	+	+	NA/M	-	Moderate
Rugel and Brauer, 2020	+	+	-	+	+	-	+	P	+	-	NA/M	NA/M	+	+	NA/M	+	Moderate
Vesterinen et al., 2017	+	+	-	+	+	-	P	-	+	-	+	+	+	+	+	+	Moderate
Lin et al., 2022	+	-	-	P	+	-	P	+	+	-	+	+	+	+	+	+	Low
Yu et al., 2022	P	-	-	+	+	+	+	+	P	-	NA/M	NA/M	+	-	NA/M	+	Low
Bell et al., 2013	P	-	-	-	-	-	-	P	-	-	+	-	-	-	+	+	Critically Low
Bell et al., 2014	P	-	-	-	-	-	P	P	-	-	+	-	-	-	+	+	Critically Low
Fuller et al., 2017	P	-	-	-	-	-	P	P	-	-	NA/M	NA/M	-	+	NA/M	+	Critically Low
Heo et al., 2019	+	-	-	-	-	+	P	P	-	-	NA/M	NA/M	-	-	NA/M	-	Critically Low
Li et al., 2022	+	P	-	+	+	+	P	+	-	-	+	-	-	+	+	+	Critically Low
Son et al., 2021	P	-	-	P	+	-	P	+	-	-	NA/M	NA/M	-	-	NA/M	-	Critically Low
Thayamballi et al., 2021	+	-	-	P	+	-	P	-	-	-	+	-	-	+	-	-	Critically Low
Weinmayr et al., 2010	P	-	-	-	-	-	-	P	-	-	+	-	-	+	+	-	Critically Low
*Scoping Reviews*																	
Deziel et al., 2020	P	-	-	+	-	-	P	P	NA/S	-	NA/M	NA/M	NA/S	NA/S	NA/M	+	Low
Payne-Sturges et al., 2023	+	-	-	P	+	-	P	+	NA/S	-	NA/M	NA/M	NA/S	NA/S	NA/M	+	Low
Munoz-Pizza et al., 2020	P	-	-	-	-	-	P	-	NA/S	-	NA/M	NA/M	NA/S	NA/S	NA/M	+	Critically Low
Namin et al., 2021	-	-	-	P	-	+	-	-	NA/S	-	NA/M	NA/M	NA/S	NA/S	NA/M	-	Critically Low
Ruiz Jdel et al., 2016	P	-	-	-	-	-	P	-	NA/S	-	NA/M	NA/M	NA/S	NA/S	NA/M	+	Critically Low

*AMSTAR-2 critical domain; + = Yes; - = No; P = Partial yes; NA/M = No meta-analysis conducted; NA/S = Not applicable (scoping review)

*PECO* Population, Exposure, Comparator, Outcome statement, *RoB* risk of bias, *MA* meta-analysis, *COI* conflict of interest

For the critical criteria, only four reviews of the 18 (22%) pre-published a protocol (AMSTAR 2), though more than half of the reviews (11/18) conducted a comprehensive search of the literature (AMSTAR 4). Most reviews (12/18) summarized reasons for exclusion at full text in a flow chart rather than providing a complete list of excluded studies, receiving a ‘partial yes’ for AMSTAR 7. Of the 13 systematic reviews, five (38%) used a satisfactory technique for assessing risk of bias (AMSTAR 9) and accounted for risk of bias in the interpretation of the results (AMSTAR 13). All the studies that conducted a meta-analysis (*n* = 7) used appropriate methods for the statistical combination of results (AMSTAR 11).

For the non-critical criteria, none of the included reviews met criteria 3 or 10, as the authors failed to explain their selection of study designs (AMSTAR 3) and did not report funding and conflict of interest information for each primary study (AMSTAR 10). Most reviews (17/18) included the components of PI/ECO (AMSTAR 1). For criteria 5 and 6, 50% of reviews completed study selection in duplicate, while only 28% completed data extraction in duplicate. The majority of reviews (13/18) received a ‘partial yes’ or ‘yes’ for their description of the primary studies in AMSTAR 8. Only two assessed the potential impact of the risk of bias on the results of the evidence synthesis (AMSTAR 12). Most of the systematic reviews included a discussion of heterogeneity (AMSTAR 14) and publication bias (AMSTAR 15). Two-thirds of reviews (12/18) reported potential conflicts of interest (AMSTAR 16).

### Primary study overlap

Most reviews included in our analysis asked unique research questions, eliminating the need to conduct detailed overlap assessments. However, two reviews both evaluated SES and race/ethnicity as potential effect modifiers in the relationship between air pollution and child neurodevelopment (Table S7) [24, 50]. Using the GROOVE tool, we found overlap (CCA = 23.1%) in primary studies. We reported the identified overlap and presented the results of all relevant reviews, regardless of topic overlap, because we are not conducting a meta-analysis. Rather, we are only summarizing the available evidence due to insufficient studies in any one topic area [25].

### The joint effect of non-chemical stressors

We further evaluated seven reviews that were rated ‘moderate’ or ‘low’ quality, two of which were scoping reviews that did not analyze the data but summarized the existing evidence. The five included systematic reviews reported associations between air and environmental pollution, non-chemical stressors, and a variety of health endpoints, including asthma, birth outcomes, obesity, and neurodevelopment. Reviews considered a variety of non-chemical stressors, each uniquely defined by the authors (Table 3).

**Table 3:** Non-chemical stressors evaluated in systematic and scoping reviews that were rated moderate or low quality (*n* = 7).

Review	Non-Chemical Stressor Evaluated	Definition/Measurement
Rodriguez-Villamizar et al. 2016	Socioeconomic position (SEP)	Any single or combined measure related to income, education, or occupation. Aggregated SEP measures were defined as area-based SEP measures generally based on census data.
Vesterinen et al. 2017	Stress	*Human studies:* Dichotomized stress exposure variables into ‘high’ and ‘low’ based on race, educational attainment, socioeconomic status or social class, maternal mood, and life events.
		*Animal studies:* restraint, heat, nest material restriction, no light, restraint with bright light, and various stressors
Rugel and Brauer 2020	Noise	Restricted to non-occupational contexts, to include the noise generated by industrial environments but not exposures to sounds that occur inside settings, such as factories.
	Natural spaces	Comprising surrounding greenness based on surface-reflectance values derived from satellite-based platforms; greenspace, defined as any area containing or comprising vegetation and including parks, gardens, playgrounds, and street trees; and blue spaces, defined as any area containing water, such as lakes, rivers, beaches, and decorative fountains.
	Neighborhood walkability	Aspects of the urban form that are known to impact pedestrian behavior including: design and connectivity of street networks; density of residential and retail properties; and land-use mix, or the range of destination types within an area.
Lin et al. 2022	Country development status	“Developed” countries vs “developing” countries
Yu et al. 2022	Socioeconomic status (SES)	Markers for socioeconomic status (maternal education, race, and neighborhood deprivation and other characteristics from census data)
Deziel et al. 2020	Urban/Rural	As defined in primary studies using Zip codes
	Private water and/or environmental law violations	Private water and/or environmental law violations County ratio of water wells divided by the number of births
Payne-Sturges et al. 2023	Family income	Receipt of medical assistance
	SES, Race/ethnicity	Race, ethnicity, and other indicators of sociodemographic and socioeconomic disadvantage

Non-chemical stressors listed are those defined by the review authors.

### Summary of review findings

**Fig. 2:**
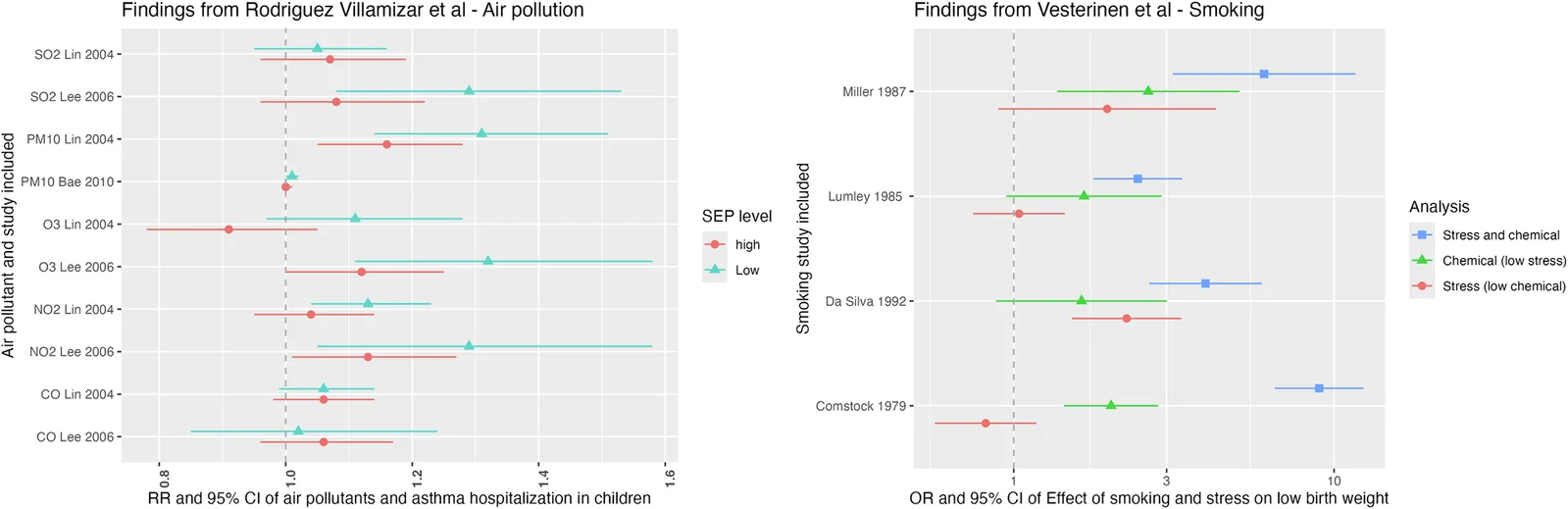
Findings reported in moderate-quality systematic reviews that explored the impact of individual-level stressors and showed the strengthened association between exposure and adverse health outcomes when both stress and exposure are considered jointly. SEP socioeconomic position, OR odds ratio, RR Risk Ratio, CI confidence interval.

The seven reviews that were of low or moderate quality explored a range of exposures, outcomes, and stressors and came to various conclusions about the impact of non-chemical stressors (Table 4 & Table S8). Two reviews conducted a certainty of the evidence assessment using GRADE, labeled as “tier 1”, GRADE is a validated tool that reflects how confident once can be that observed effects in a study reflects the true effect [62]. These two reviews included both individual and country-level stressors. Lin et al., found that PM had a stronger effect on obesity risk when accounting for the country’s development status (developed vs developing countries) (Particular Matter (PM) 2.5 Risk Ratio (RR): 1.16, 95% Confidence Interval (CI): 1.1-1.21; PM10 RR: 1.09, 95% CI: 1.07, 1.12), with low certainty of the evidence. Rugel and Brauer et al. did not conduct a meta-analysis but concluded that an individual’s exposure to noise was associated with an increased risk of traffic-related air pollution (TRAP) on cardiovascular disease, mortality, type 2 diabetes, and adverse reproductive outcomes (with a moderate certainty of the evidence) and that exposure to green spaces was associated with a *mitigation* of the effect of TRAP on chronic respiratory disease (with a low certainty of the evidence)[47, 49]. Three studies did not assess the certainty of the evidence but were rated as moderate quality with the AMSTAR tool (tier2) [23, 46, 50]. Rodriguez-Villamizar et al. found an increased risk of asthma-related hospitalizations among children in the low SES group, and Vesterinen et al. found that smoking increased the odds of low birthweight among individuals categorized in the high-stress group (Fig. 2). Detailed results of each review can be found in Table S9. Only one review included animal evidence (Vesterinen et al.); however authors reported inconsistent results, a high risk of bias, and findings were further limited by a small number of studies on each chemical [23].

**Table 4:** Review findings and conclusions by exposure and outcome of the moderate and low-quality systematic reviews included in the systematic review (*n* = 5).

Systematic Review	Population	Chemical Exposure	Non-Chemical Stressor^†^	Outcome	number primary studies (date range)	Meta-Analysis Results	Certainty of the Evidence	Review Author’s Conclusion (verbatim)^††^
**Tier 1 (With a certainty of the evidence assessment)**								
Lin et al. 2022	Humans	PM2.5	Country development status	Obesity	19 (2019–2021)	Exposure to PM2.5 (RR: 1.16, 95% CI: 1.11–1.21) and PM10 (RR: 1.09, 95% CI: 1.07–1.12) with per 10 μg/m3 increment could increase the risk of obesity in the global population	Low	No matter what the type of the PM, PM has a stronger effect on developing countries than developed countries.
	Humans	PM10	Country development status	Obesity	15 (2014–2021)		Low	
Rugel and Brauer 2020	Individuals residing in largely urban areas	Traffic-related air pollution	Noise	Cardiovascular disease	13 (2006–2019)	NA	Moderate	Sufficient evidence for small increases in CVD morbidity with higher noise exposures; Sufficient evidence for ***lack*** of effect of TRAP exposures on increased CVD morbidity
	Individuals residing in largely urban areas	Traffic-related air pollution	Noise	Mortality	6 (2008–2019)		Moderate	Limited evidence for small increases in mortality with higher noise exposures; Sufficient evidence for small increases in mortality with higher TRAP exposures
	Individuals residing in largely urban areas	Traffic-related air pollution	Noise	Reproductive outcomes	5 (2014–2019)		Moderate	Limited evidence for small increases in adverse reproductive outcomes with higher TRAP exposures; Limited evidence for ***lack*** of effect of noise on adverse reproductive outcomes
	Individuals residing in largely urban areas	Traffic-related air pollution	Noise	Type 2 diabetes	4 (2012–2018)		Moderate	Inadequate evidence for effects of either TRAP or noise on Type 2 diabetes after adjustment for other exposures
	Individuals residing in largely urban areas	Traffic-related air pollution	Natural spaces	Chronic respiratory disease	5 (2015–2017)		Low	Limited evidence for small decreases in chronic respiratory disease with higher natural space exposures; Limited evidence for partial *mitigation* of TRAP effects on chronic respiratory disease with higher natural space exposures
	Individuals residing in largely urban areas	Traffic-related air pollution	Natural spaces	Physical health	20 (2011–2018)		Low	Inadequate evidence for all outcomes
	Individuals residing in largely urban areas	Traffic-related air pollution	Natural spaces, noise	Reproductive outcomes	3 (2014–2019)		Moderate	Limited evidence for small decreases in adverse reproductive outcomes with higher natural space exposures
	Individuals residing in largely urban areas	Traffic-related air pollution	Natural spaces, noise	Mortality	1 (2017)		Moderate	Inadequate evidence for all outcomes
	Individuals residing in largely urban areas	Traffic-related air pollution	Natural spaces, noise	Cardiovascular disease	2 (2018–2019)		Moderate	Inadequate evidence for all outcomes
	Individuals residing in largely urban areas	Traffic-related air pollution	Natural spaces, noise	Diabetes	2 (2017–2018)		Moderate	Inadequate evidence for all outcomes
	Individuals residing in largely urban areas	Traffic-related air pollution	Natural spaces, noise, neighborhood walkability	Health	3 (2014–2019)		Moderate	Inadequate evidence for all outcomes
**Tier 2 (Without a certainty of the evidence assessment)**								
Rodriquez-Villamizar et al. 2016	Children	Air pollution	Socioeconomic position	Asthma-related hospitalizations	5 (2004–2013)	NA	Did not assess	The results revealed that associations between hospitalizations and air pollutants seem to have a stronger negative influence on children living in low SEP conditions. However, confirmation of the effect modification by statistically significant interactions between air pollutants and SEP was evident only in one out of five studies, probably related to sample size limitations of the original studies.
	Children	Air pollution	Socioeconomic position	Asthma-related ED visits/calls	3 (2008–2014)			Overall, we found three studies failing to demonstrate any SEP differential effect on the association between pollutant exposure and asthma-related ED visits/calls.
	Children	Air pollution	Socioeconomic position	Ambulatory visits	1 (2009)			None
	Children	Air pollution	Socioeconomic position	Repeat hospital visits	1 (2009)			None
Vesterinen et al. 2017	Humans	Smoking	Stress	Birth weight	9 (1984–2011)	Smoking was associated with significantly increased odds of LBW in the high stress (low SES) group (combined exposure, OR 4.75 (2.46 ± 9.16); Χ2 = 33.28, I2 = 91.0%) compared to the low stress group (high SES) (OR 1.95 (95% CI 1.53 ± 2.48); Χ2 = 1.49, I2 = 0%). There was also an elevated but not statistically significant odds ratio of LBW for high stress (low SES) compared to low stress among non-smokers (OR 1.34 (0.82 ± 2.20); Χ2 = 16.24, I2 = 81.5%)	Did not assess	We found evidence for a combined effect of chemical exposure and stress on birth weight.
	Humans	Air pollution	Stress	Birth weight	6 (1985–2011)			
	Humans	Smoking	SES	Low birth weight	4 (1971–1992)			Our meta-analysis results on the effect of smoking and low SES, found a clear trend towards a reduction in birth weight for smoking in both the absence and presence of low SES.
	Animals (mice, rats)	Various chemicals	Stress	Pre- or post-natal offspring weight	22 (1989–2013)			In general, the findings from animal studies were not consistent; however, the dataset was extremely heterogeneous and sparse.
Yu et al. 2022	Children	Air pollution	Education	Autism spectrum disorder	3 (2013–2017)	NA	Did not assess	Three studies (2 case-control and 1 ecological design) demonstrated no consistent pattern of effect modification of the air pollution-ASD association by education level.
	Children	Air pollution	Neighborhood characteristics	Autism spectrum disorder	4 (2010–2019)			Results from studies of effects of neighborhood deprivation on air pollution-ASD associations were inconsistent.
	Children	Air pollution	Race	Autism spectrum disorder	1 (2018)			None

*SES* socioeconomic status, *COE* certainty in the body of evidence, *ED* emergency department, *PM* particulate matter, *TRAP* traffic-related air pollution, *CVD* cardiovascular disease

† Defined in Table 3.

†† “None” indicates that the review authors did not come to any conclusions on associations between the stated exposure and outcome.

## Discussion

The goal of this overview was to identify and evaluate the quality of the existing literature on how non-chemical stressors influence the relationship between environmental exposures and adverse health outcomes, utilizing systematic review methodologies to examine human and animal evidence. Reviews suggest that there is an increased effect of exposure on adverse health outcomes when chemical and non-chemical stressors are considered together. The findings from the two systematic reviews that conducted a certainty of the evidence assessment suggest a stronger impact on health outcomes with the consideration of the non-chemical stressors of exposure to noise and the country’s development status [47, 49]. While these studies evaluated area- and geographic-level non-chemical stressors, they provide further evidence that non-chemical stressors can moderate or interact with the exposure-outcome relationship and justify the need for additional high-quality systematic reviews evaluating their impact. The lack of certainty assessment for most of the included systematic reviews meant that we could not rely on their findings to make a conclusive statement of effect. Our evaluation and synthesis of these systematic and scoping reviews further revealed large evidence and methodological gaps in the literature on this topic, including lack of certainty of the evidence assessment, the overall low quality of the identified reviews and variability in the definition, conceptualization, and use of non-chemical stressors in the reviews.

### Systematic and scoping review quality

The majority of included reviews lacked systematic and pre-established methods for evidence synthesis. Establishing and documenting methods prior to conducting the systematic review is a critical component of a systematic review, reducing the impact of author’s biases, promoting transparency, and allowing for replication [63], however, only four of the reviews included in our overview pre-published a protocol. Evaluating primary studies’ risk of bias is also a key element of a systematic review [26], though most of the included reviews did not conduct a risk of bias assessment.

Further, three of the reviews that did assess risk of bias used tools lacking scientific justification, including the Newcastle-Ottawa Scale (NOS), and the ROBINS-E tool. Both methods numerically score studies, which has been criticized by the expert ad hoc committees convened by the National Academy of Sciences, Engineering and Medicine (NASEM) as a poor measure of overall study quality [64] and for its ability to erroneously exclude studies from analyses [65, 66]. Only two-thirds of the reviews reported potential author and financial conflicts of interest, and none of the reviews reported funding and conflict of interest information for their primary studies. This lack of transparency could lead to biased results as industry-sponsored studies tend to report results and conclusions that are more likely to favor the study sponsor [67]. Of the five systematic reviews, only two assessed the certainty of the evidence, which diminishes our ability to rely on the findings for decision-making in environmental health and limits the studies we can rely upon to quantitatively incorporate the impact of non-chemical stressors into risk assessment methodologies.

There is an immense need for higher-quality systematic reviews on this topic. The National Academies of Sciences, Engineering, and Medicine have published recommendations on methods for robust systematic review. They recommend the Navigation Guide [26] and the NTP-OHAT [68] systematic review method as tools regulatory agencies should apply for risk assessment. Both methods offer a robust systematic review methodology, including a qualitative risk of bias assessment and an evaluation of the certainty of the evidence and do not encourage the removal of primary studies from analysis. These methods should be consistently applied across the field of environmental health so that future analyses can strengthen CRA methodologies.

### Evidence gaps

Most reviews evaluating human evidence used socioeconomic indicators as the non-chemical stressors, though the measurement of SES varied widely. Some studies used individual-level measures of SES, or proxies for SES, such as education, income, health insurance status, or employment status, while others utilized family-level, community-level, or even country-level measures, such as a country’s development status, or urban vs rural living environments. The heterogeneity in definitions, conceptualizations, and measurements of non-chemical stressors prevented us from comparing or directly combining results across reviews. Furthermore, using SES as a measure for vulnerability or stress may also assume that individuals of the same income level have the same level of a given stressor. Though it is well-established in the literature that lower levels of SES are associated with higher levels of some severe forms of stress (e.g. financial strain), the relationship between stress and SES is nuanced, and there is evidence, for example, that higher levels of education may induce greater stress due to job pressure, overwork, and work/family conflict [69]. Adding additional nuance, individuals with higher status jobs or higher SES may have additional resources to cope with exposure to stressors than individuals with lower ranking jobs or SES [70]. Studies also often compared health across only a few categories (e.g., high SES vs low SES, poor vs not poor), which may obscure important health gradients related to SES [71].

The impact of non-chemical stressors on the relationship between exposure to environmental pollutants and adverse health outcomes depends on how well the measured variables capture social stressors. While race/ethnicity is commonly used as a proxy for exposure to complex social stressors [23, 50], such as racism, it may not fully account for nuances in individuals’ or community experiences with psychosocial stress that arise from structural oppression and the buffering effect of sharing a community with people from similar racial and ethnic backgrounds [8, 72, 73]. Thus, using race/ethnicity as a proxy for social stressors may result in an over- or underestimate of the impact of social stressors. Further, the assumption that certain racial and ethnic categories are equivalent to increased stress may further reify stereotypical and oversimplified understanding of the role racial marginalization plays in the relationships between environmental exposures and health.

Of the thousands of reviews identified in our search, relatively few examined non-chemical stressors in conjunction with the exposure-outcome relationship. Of those reviews included in this overview, few studies included indicators for non-chemical stressor exposures beyond SES and race/ethnicity (those that did, evaluated greenness, neighborhood walkability, or noise). Additionally, a few reviews provided a description of how the non-chemical stressors were conceptualized, including defining and justifying the indicators used to assign exposure to the non-chemical stressor, including for SES and race/ethnicity. Except for Payne-Sturges et al. [24], most review authors also failed to discuss potential biological mechanisms associated with non-chemical stressor exposures, which limits options for combining studies in meta-analyses or developing an overall statement on the health impact of a non-chemical stressor.

### Strengths and limitations

This is the first overview to evaluate the influence of extrinsic non-chemical stressors on the relationship between environmental chemical exposures and adverse health effects. We utilized best practices in systematic review, including rigorous methods informed by Cochrane’s guidance for Overviews of Reviews [25] and the Navigation Guide [26]. We established our methodology in a published protocol to reduce bias and increase transparency. We also used and adapted AMSTAR-2, an established tool, to evaluate the quality of reviews on this topic [38]. We evaluated a wide range of exposures and outcomes, and our inclusion of scoping reviews provided us with a more comprehensive understanding of the scope of the evidence on this topic. Our findings identified several evidence gaps in the existing literature, including failing to adhere to important methodological steps required for a rigorous systematic review, such as conducting certainty of the evidence assessments, which can help to inform future research and improve how chemical and non-chemical stressors are combined and can be integrated into CRA methodologies.

Our overview does have several limitations. We did not search the gray literature or the reference lists of included studies, limiting the body of evidence evaluated. In addition, the scoping reviews we included did not conduct risk of bias assessments or meta-analyses and few conducted certainty of the evidence assessments. Due to time constraints of our funding, we did not conduct certainty assessments for any reviews for reviews that did not conduct one, limiting the studies on which we could draw conclusions on the interaction or modifying effect of non-chemical stressors. Hence, our summaries were dependent on how the authors of the included reviews evaluated and synthesized the evidence.

### Recommendations

We undertook this overview of systematic reviews to identify, evaluate, and synthesize relevant systematic and scoping reviews that assess and describe the impact of chemical and non-chemical stressors on adverse health outcomes and provide recommendations to the Environmental Protection Agency (EPA) for incorporating non-chemical stressors into regulatory analyses. We identified several areas where the literature can be improved to further advance these recommendations.

First, based on the AMSTAR-quality assessment, we rated 7 studies as moderate or low quality; 11 of 18 reviews were scored as critically low and were excluded from analysis, and 4 additional studies were scored as low-quality. The majority of reviews failed to pre-publish their study methods impacting their quality score. To improve the overall quality of systematic reviews, we recommend that authors conducting systematic reviews in environmental health pre-publish their protocols and methods for data synthesis and evaluation. Further, we recommend training in systematic review methods for use in the field of environmental health and regulatory agencies.

Second, there was a wide variability in the definition, use, and conceptualization of non-chemical stressors in the identified systematic and scoping reviews. We recommend that future studies exploring co-exposures to chemical and non-chemical stressors should adopt a more expansive and comprehensive definition of stressors and identify ways to more directly operationalize stressors, such as systemic racism, food insecurity, exposure to violence, adverse life events, and sexual orientation and gender identity discrimination. There is also a need for additional research on effect modification and interaction by non-chemical stressors for a more diverse array of chemical and other environmental exposures, beyond air pollution, to inform future risk assessment approaches in ways that account for sensitive populations.

Our analysis also identified several key areas for advancing our understanding of the impact of non-chemical stressors on exposures and outcomes. Given the robust evidence on air pollution and childhood asthma and air pollution and mortality risk in adults, we recommend that future studies evaluate the modifying effect of non-chemical stressors on these evidence-rich exposure/outcome relationships. Environmental pollution, including lead, and childhood neurodevelopment is another relevant and evidence-rich relationship that needs additional research, including through systematic review, and can be used by decision-makers at EPA to quantify the additional risk posed by non-chemical stressors. We have proposed a future systematic review exploring the impact of NCS on exposure to lead, particulate matter, and organophosphate chemicals on child neurodevelopment [74].

## Conclusion

Evidence suggests that the combined influence of chemical exposures and non-chemical stressors (NCS) is more strongly associated with adverse health outcomes than when each is considered in isolation, underscoring the need for regulatory frameworks to account for these interactions. Current risk assessment methods used by regulatory agencies like the US EPA do not adequately account for the impact of NCS on chemical risk, limiting public health protections. In this overview, we applied best-practice systematic review methodologies to identify scoping and systematic reviews that examined the combined effects of chemical and NCS on adverse health outcomes, ultimately informing improved methods for chemical risk assessment. However, only two of the included reviews conducted certainty assessments using GRADE, and most failed to implement key systematic review methodological steps, such as having pre-registered protocols and transparent approaches to evidence synthesis. These limitations reduced the quality of the available evidence base and limited our ability to draw conclusions about the modifying effect of NCS on the exposure-outcome relationship from the available studies. Future research should employ rigorous systematic review methodologies and broaden consideration of NCS to improve our understanding of the combined effect of chemical and NCS. This will ultimately strengthen the evidence base for regulatory decision-making and advance CRA methods that better protect susceptible populations.

## Supplementary information

## Data Availability

All data generated or analyzed during this study are included in this published article and its supplementary information files.

## Footnotes

1 We used a rigorous method to determine if a review used the required methodological principles to meet the definition of a systematic review, as narrative reviews are often incorrectly categorized as such in the environmental health literature [29].
