# Supplementary material for: The combined impact of chemical and non-chemical stressors on adverse health outcomes: an overview of reviews from the epidemiological literature and experimental animal studies
Source: J Expo Sci Environ Epidemiol. 2026 Jun 30;36(5):1013–29. doi: 10.1038/s41370-026-00862-x (PMC13538057; doi:10.1038/s41370-026-00862-x)
Supplement: Supplementary file 1 [file 41370_2026_862_MOESM1_ESM.pdf]

Title

The combined impact of chemical and non-chemical stressors on adverse health outcomes: an overview of reviews from the epidemiological literature and experimental animal studies

Authors

Jessica Trowbridge, PhD, MPH,[1] Emily Lasher, MSPH,[1] Xing Gao, PhD,[1] Nicholas Chartres, PhD,[2] Rashmi Joglekar, PhD,[1] Rachel Morello-Frosch PhD, MPH[3], Mary A. Fox[4], Devon Payne-Sturges[5], Jessie P. Buckley[6], Tracey J Woodruff, PHD, MPH[1*]

[1]Program on Reproductive Health and the Environment, Department of Obstetrics, Gynecology, and Reproductive Sciences, University of California, San Francisco, CA, USA.

[2]School of Pharmacy, Faculty of Medicine and Health, The University of Sydney, Sydney, New South Wales, Australia.

[3]Department of Environmental Science and Policy Management and School of Public Health, University of California, Berkeley. Dr. Morello-Frosch stepped off the project as of May 1, 2024.

[4] Risk Sciences and Public Policy Institute, Department of Health Policy and Management, Johns Hopkins Bloomberg School of Public Health, Baltimore, MD, USA

[5] Department of Global, Environmental and Occupational Health, University of Maryland School of Public Health, College Park, MD

[6] Gillings School of Global Public Health, Department of Epidemiology, University of North Carolina at Chapel Hill, Chapel Hill, NC, USA

*Corresponding Author
Tracey.Woodruff@ucsf.edu

Table of Contents

Table S1. PRIOR Checklist
Table S2. PRISMA Guidelines for Systematic Reviews
Table S3. Search Strings
Table S4. Modified AMSTAR 2.0 Tool to Evaluate Environmental Health Systematic Reviews
Table S5. Justifications for Exclusion at Full Text (n=79)
Table S6. Justifications for Exclusion at Evaluation (n=4)
Table S7. Results of Overlap Assessment for Two Reviews Evaluating the Impact of Air Pollution on Child Neurodevelopment (GROOVE Tool)
Table S8. Population, Exposures, and Outcomes of Included Scoping Reviews
Table S9. Detailed Review Results
Table S10. Deviations from Protocol

Table S1.

PRIOR Checklist

(Gates M, Gates A, Pieper D, et al. Reporting guideline for overviews of reviews of healthcare interventions: development of the PRIOR statement. *BMJ* 2022;378:e070849. doi:10.1136/bmj-2022-070849.)

**Section**<br>Topic	**#**	**Item**	**Location reported**
**TITLE**			
Title	1	Identify the report as an overview of reviews.	*line 2*
**ABSTRACT**			
Abstract	2	Provide a comprehensive and accurate summary of the purpose, methods, and results of the overview of reviews.	*lines 26-55*
**INTRODUCTION**			
Rationale	3	Describe the rationale for conducting the overview of reviews in the context of existing knowledge.	*lines 81-87*
Objectives	4	Provide an explicit statement of the objective(s) or question(s) addressed by the overview of reviews.	*lines 99- 109*
**METHODS**			
Eligibility criteria	5a	Specify the inclusion and exclusion criteria for the overview of reviews. If supplemental primary studies were included, this should be stated, with a rationale.	*lines 113-167*
	5b	Specify the definition of 'systematic review' as used in the inclusion criteria for the overview of reviews.	*lines 113-11*<br>*Table S2*
Information sources	6	Specify all databases, registers, websites, organizations, reference lists, and other sources searched or consulted to identify systematic reviews and supplemental primary studies (if included). Specify the date when each source was last searched or consulted.	*lines 168- 173*
Search strategy	7	Present the full search strategies for all databases, registers and websites, such that they could be reproduced. Describe any search filters and limits applied.	*Table S3*
Selection process	8a	Describe the methods used to decide whether a systematic review or supplemental primary study (if included) met the inclusion criteria of the overview of reviews.	*Lines 179-184*
	8b	Describe how overlap in the populations, interventions, comparators, and/or outcomes of systematic reviews was identified and managed during study selection.	*Lines 265-26*
Data collection process	9a	Describe the methods used to collect data from reports.	*N/A - did not collect reports*
	9b	If applicable, describe the methods used to identify and manage primary study overlap at the level of the comparison and outcome during data collection. For each outcome, specify the method used to illustrate and/or quantify the degree of primary study overlap across systematic reviews.	*Lines 268-27*
	9c	If applicable, specify the methods used to manage discrepant data across systematic reviews during data collection.	*NA*
Data items	10	List and define all variables and outcomes for which data were sought. Describe any assumptions made and/or measures taken to identify and clarify missing or unclear information.	*lines 146-149 &*<br>*185-201*
Risk of bias assessment	11a	Describe the methods used to *assess* risk of bias or methodological quality of the included systematic reviews.	*AMSTAR*<br>*Lines 213-244*
	11b	Describe the methods used to *collect* data on (from the systematic reviews) and/or *assess* the risk of bias of the primary studies included in the systematic reviews. Provide a justification for instances where flawed, incomplete, or missing assessments are identified but not re-assessed.	*Lines 198-*<br>*211; 258-260*
	11c	Describe the methods used to *assess* the risk of bias of supplemental primary studies (if included).	*NA*
Synthesis methods	12a	Describe the methods used to summarize or synthesize results and provide a rationale for the choice(s).	*Lines 273-281*
	12b	Describe any methods used to explore possible causes of heterogeneity among results.	*NA*
	12c	Describe any sensitivity analyses conducted to assess the robustness of the synthesized results.	*NA*
Reporting bias assessment	13	Describe the methods used to *collect* data on (from the systematic reviews) and/or *assess* the risk of bias due to missing results in a summary or synthesis (arising from reporting biases at the levels of the systematic reviews, primary studies, and supplemental primary studies, if included).	*Lines 198*
Certainty assessment	14	Describe the methods used to *collect* data on (from the systematic reviews) and/or *assess* certainty (or confidence) in the body of evidence for an outcome.	*Lines 253-263*
**RESULTS**			
Systematic review and supplemental primary study selection	15a	Describe the results of the search and selection process, including the number of records screened, assessed for eligibility, and included in the overview of reviews, ideally with a flow diagram.	*Lines 283-289*
	15b	Provide a list of studies that might appear to meet the inclusion criteria, but were excluded, with the main reason for exclusion.	*Tables S5&S6*

**Section** Topic	**#**	**Item**	**Location reported**
Characteristics of systematic reviews and supplemental primary studies	16	Cite each included systematic review and supplemental primary study (if included) and present its characteristics.	Table1
Primary study overlap	17	Describe the extent of primary study overlap across the included systematic reviews.	lines 335- 342
Risk of bias in systematic reviews, primary studies, and supplemental primary studies	18a	Present assessments of risk of bias or methodological quality for each included systematic review.	Lines 297-319
	18b	Present assessments (*collected* from systematic reviews or *assessed* anew) of the risk of bias of the primary studies included in the systematic reviews.	NA
	18c	Present assessments of the risk of bias of supplemental primary studies (if included).	NA
Summary or synthesis of results	19a	For all outcomes, summarize the evidence from the systematic reviews and supplemental primary studies (if included). If meta-analyses were done, present for each the summary estimate and its precision and measures of statistical heterogeneity. If comparing groups, describe the direction of the effect.	Line 335-348 Table S9
	19b	If meta-analyses were done, present results of all investigations of possible causes of heterogeneity.	NA
	19c	If meta-analyses were done, present results of all sensitivity analyses conducted to assess the robustness of synthesized results.	NA
Reporting biases	20	Present assessments (*collected* from systematic reviews and/or *assessed* anew) of the risk of bias due to missing primary studies, analyses, or results in a summary or synthesis (arising from reporting biases at the levels of the systematic reviews, primary studies, and supplemental primary studies, if included) for each summary or synthesis assessed.	Table 2
Certainty of evidence	21	Present assessments (*collected* or *assessed* anew) of certainty (or confidence) in the body of evidence for each outcome.	Table 4
**DISCUSSION**			
Discussion	22a	Summarize the main findings, including any discrepancies in findings across the included systematic reviews and supplemental primary studies (if included).	lines 372 - 387
	22b	Provide a general interpretation of the results in the context of other evidence.	lines 372-428
	22c	Discuss any limitations of the evidence from systematic reviews, their primary studies, and supplemental primary studies (if included) included in the overview of reviews. Discuss any limitations of the overview of reviews methods used.	lines 464- 470
	22d	Discuss implications for practice, policy, and future research (both systematic reviews and primary research). Consider the relevance of the findings to the end users of the overview of reviews, e.g., healthcare providers, policymakers, patients, among others.	Lines 450-489
**OTHER INFORMATION**			
Registration and protocol	23a	Provide registration information for the overview of reviews, including register name and registration number, or state that the overview of reviews was not registered.	line 120
	23b	Indicate where the overview of reviews protocol can be accessed, or state that a protocol was not prepared.	line 122
	23c	Describe and explain any amendments to information provided at registration or in the protocol. Indicate the stage of the overview of reviews at which amendments were made.	Supplement
Support	24	Describe sources of financial or non-financial support for the overview of reviews, and the role of the funders or sponsors in the overview of reviews.	line 25
Competing interests	25	Declare any competing interests of the overview of reviews' authors.	line 30
Author information	26a	Provide contact information for the corresponding author.	line 22
	26b	Describe the contributions of individual authors and identify the guarantor of the overview of reviews.	line 519
Availability of data and other materials	27	Report which of the following are available, where they can be found, and under which conditions they may be accessed: template data collection forms; data collected from included systematic reviews and supplemental primary studies; analytic code; any other materials used in the overview of reviews.	line 534

Table S2. PRISMA Guidelines for Systematic Reviews

To meet the minimum criteria for systematic review, the paper must include all of the following highlighted information:

Section and Topic	Item #	Checklist item
**TITLE**		
Title	1	Identify the report as a systematic review.
**ABSTRACT**		
Abstract	2	See the PRISMA 2020 for Abstracts checklist.
**INTRODUCTION**		
Rationale	3	Describe the rationale for the review in the context of existing knowledge.
Objectives	4	Provide an explicit statement of the objective(s) or question(s) the review addresses.
**METHODS**		
Eligibility criteria	5	Specify the inclusion and exclusion criteria for the review and how studies were grouped for the syntheses.
Information sources	6	Specify all databases, registers, websites, organisations, reference lists and other sources searched or consulted to identify studies. Specify the date when each source was last searched or consulted.
Search strategy	7	Present the full search strategies for all databases, registers and websites, including any filters and limits used.
Selection process	8	Specify the methods used to decide whether a study met the inclusion criteria of the review, including how many reviewers screened each record and each report retrieved, whether they worked independently, and if applicable, details of automation tools used in the process.
Data collection process	9	Specify the methods used to collect data from reports, including how many reviewers collected data from each report, whether they worked independently, any processes for obtaining or confirming data from study investigators, and if applicable, details of automation tools used in the process.
Data items	10a	List and define all outcomes for which data were sought. Specify whether all results that were compatible with each outcome domain in each study were sought (e.g. for all measures, time points, analyses), and if not, the methods used to decide which results to collect.
	10b	List and define all other variables for which data were sought (e.g. participant and intervention characteristics, funding sources). Describe any assumptions made about any missing or unclear information.
Study risk of bias assessment	11	Specify the methods used to assess risk of bias in the included studies, including details of the tool(s) used, how many reviewers assessed each study and whether they worked independently, and if applicable, details of automation tools used in the process.

Section and Topic	Item #	Checklist item
Effect measures	12	Specify for each outcome the effect measure(s) (e.g. risk ratio, mean difference) used in the synthesis or presentation of results.
Synthesis methods	13a	Describe the processes used to decide which studies were eligible for each synthesis (e.g. tabulating the study intervention characteristics and comparing against the planned groups for each synthesis (item #5)).
	13b	Describe any methods required to prepare the data for presentation or synthesis, such as handling of missing summary statistics, or data conversions.
	13c	Describe any methods used to tabulate or visually display results of individual studies and syntheses.
	13d	Describe any methods used to synthesize results and provide a rationale for the choice(s). If meta-analysis was performed, describe the model(s), method(s) to identify the presence and extent of statistical heterogeneity, and software package(s) used.
	13e	Describe any methods used to explore possible causes of heterogeneity among study results (e.g. subgroup analysis, meta-regression).
	13f	Describe any sensitivity analyses conducted to assess robustness of the synthesized results.
Reporting bias assessment	14	Describe any methods used to assess risk of bias due to missing results in a synthesis (arising from reporting biases).
Certainty assessment	15	Describe any methods used to assess certainty (or confidence) in the body of evidence for an outcome.
**RESULTS**		
Study selection	16a	Describe the results of the search and selection process, from the number of records identified in the search to the number of studies included in the review, ideally using a flow diagram.
	16b	Cite studies that might appear to meet the inclusion criteria, but which were excluded, and explain why they were excluded.
Study characteristics	17	Cite each included study and present its characteristics.
Risk of bias in studies	18	Present assessments of risk of bias for each included study.
Results of individual studies	19	For all outcomes, present, for each study: (a) summary statistics for each group (where appropriate) and (b) an effect estimate and its precision (e.g. confidence/credible interval), ideally using structured tables or plots.
Results of syntheses	20a	For each synthesis, briefly summarise the characteristics and risk of bias among contributing studies.
	20b	Present results of all statistical syntheses conducted. If meta-analysis was done, present for each the summary estimate and its precision (e.g. confidence/credible interval) and measures of statistical heterogeneity. If comparing groups, describe the direction of the effect.

Section and Topic	Item #	Checklist item
	20c	Present results of all investigations of possible causes of heterogeneity among study results.
	20d	Present results of all sensitivity analyses conducted to assess the robustness of the synthesized results.
Reporting biases	21	Present assessments of risk of bias due to missing results (arising from reporting biases) for each synthesis assessed.
Certainty of evidence	22	Present assessments of certainty (or confidence) in the body of evidence for each outcome assessed.
**DISCUSSION**		
Discussion	23a	Provide a general interpretation of the results in the context of other evidence.
	23b	Discuss any limitations of the evidence included in the review.
	23c	Discuss any limitations of the review processes used.
	23d	Discuss implications of the results for practice, policy, and future research.
**OTHER INFORMATION**		
Registration and protocol	24a	Provide registration information for the review, including register name and registration number, or state that the review was not registered.
	24b	Indicate where the review protocol can be accessed, or state that a protocol was not prepared.
	24c	Describe and explain any amendments to information provided at registration or in the protocol.
Support	25	Describe sources of financial or non-financial support for the review, and the role of the funders or sponsors in the review.
Competing interests	26	Declare any competing interests of review authors.
Availability of data, code and other materials	27	Report which of the following are publicly available and where they can be found: template data collection forms; data extracted from included studies; data used for all analyses; analytic code; any other materials used in the review.

Table S3. Search Strings

Search Summary

Source	Result Counts
PubMed	2,523
Web of Science	1,108
**Total**	**3,631**
**Total Unique**	**2,804**

Searches Run 1/31/2024

Search Details

PubMed

Group	String	Results
Susceptibility	(Activit* OR Addict* OR "addiction" OR "alcohol user" OR alcohol* OR Behavior* OR Behaviour* OR Choice* OR "Diet" OR "drug addict" OR "drug user" OR Exercis* OR Lifestyle* OR "physical activity" OR "physical inactivity" OR "Sedentary" OR smoker* OR "Substance Abuse" OR "adverse childhood events" OR "adverse childhood experience" OR "Adverse childhood experiences" OR "Bullying" OR "Childhood Stress" OR "crime" OR "Foster Care" OR "harassment" OR "household dysfunction" OR "intergenerational trauma" OR "trauma" OR victim* OR "violence" OR "allostatic load" OR crimin* OR Imprison* OR Incarcerat* OR "parental incarceration" OR "Probation" OR "co-exposure" OR "cumulative" OR "exposome" OR Metal* OR mixture* OR "nutrient" OR Pesticide* OR phthalate* OR "ableism" OR Amputee* OR "Blind" OR "Deaf" OR disab* OR handicap* OR Hearing Impair* OR Parapleg* OR Visual Impair* OR Visually Impair* OR "wheelchair" OR "academic achievement" OR "college education" OR "college level" OR "Education Access" OR "Education level" OR "educational achievement" OR "Educational Attainment" OR "English language proficiency" OR "Graduation Status" OR "highest degree" OR "Illiteracy" OR "linguistic competency" OR mentor* OR "Parental education attainment" OR "Quality of Education" OR Epigenetic* OR Food bank* OR "Food Desert" OR "food insecurity" OR "food security" OR "Food stamps" OR "hunger" OR School lunch* OR SNAP Benefit* OR "boys" OR "female" OR "Gender identity" OR "Genderqueer" OR "girls" OR "Intersex" OR "male" OR "nonbinary" OR "non-binary" OR "Gender Minority" OR "Gender Minorities" OR "sex" OR "transgender" OR "transphobia" OR "adverse outcome pathway" OR "adverse outcome pathways" OR "gene-environment interaction" OR genetic* OR genotype* OR polymorphism* OR "variability" OR variat* OR "access to healthcare" OR "Health Care Access" OR Health Care Cost* OR Health Disparit* OR "health education" OR "Health Insurance" OR "Health literacy" OR "Health Risk Behavior" OR "health warnings" OR hospital closure* OR "Insurance Access" OR "Insurance Coverage" OR "Lack of Care" OR "Quality of Health Care" OR "Underinsured" OR "Uninsured" OR Adoles* OR Adult* OR Child* OR Elder* OR Infan* OR "Senior Citizen" OR Teenage* OR "family dysfunction" OR "guardian" OR "maternal anxiety" OR Maternal* OR "ADHD" OR Adjustment Disorder* OR "Anorexia" OR "Anxiety" OR "Anxious" OR	26,522,857

	Attention Deficit* OR "Bipolar" OR "Bulimia" OR Depress* OR Dissociat* OR "Dissociative Identity Disorder" OR Eating Disorder* OR "Gender Dysphoria" OR Learning Disab* OR Mental Disorder* OR "Mental Health" OR "Neurotic" OR Schizo* OR Stress* OR Neurodivers* OR "Neurodivergent" OR "access to sport and recreation" OR "accessibility" OR "Clean Water" OR "crowded household" OR "energy networks" OR "Garbage" OR "green space" OR "greenspace" OR "group quarters" OR Highway* OR Housing Cost* OR "Housing Density" OR "infrastructure" OR "Internet Access" OR "landfill" OR "Mobile home" OR "Mobile homes" OR "neighborhood" OR "neighborhood deprivation" OR "open space" OR "Project building" OR "Public Housing" OR Public Park* OR "Public transport" OR "Quality of Housing" OR "residence characteristics" OR "residence characteristics" OR "Residential characteristics" OR "underserved" OR Walkab* OR "waste management" OR "waste treatment" OR Water Contam* OR "water supply" OR associated factor* OR related factor* OR endotoxin* OR Environment* OR "infectious agents" OR "microbial contaminants" OR "nonchemical" OR "non-chemical" OR "vector borne diseases" OR "vector-borne diseases" OR "waterborne pathogens" OR Pregnan* OR veteran* OR "Vulnerable population" OR "climate" OR "cold" OR "heat" OR "heatwave" OR "humidity" OR "noise" OR "temperature" OR allerg* OR "comorbidity" OR "morbidity" OR "multimorbidity" OR "pre-existing" OR "acculturation" OR "acculturative stress" OR African American* OR Alaska Native* OR Alaskan Native* OR American Indian* OR Asian* OR Black* OR "Caucasian" OR "colonization" OR Cultural Bias* OR discriminat* OR "Ethnicity" OR "Hispanic" OR "Immigration Status" OR "Indigenous" OR Latina* OR Latino* OR "Latinx" OR "marginalized" OR "migration status" OR Minorit* OR "minorities" OR "Minority" OR Native American* OR Native Hawaiian* OR "Oppression" OR Pacific Islander* OR "People of Color" OR "Privilege" OR "race" OR "racism" OR "Segregation" OR subpopulation* OR "Under represented groups" OR White* OR "Racialized" OR "metro" OR "nonmetro" OR "Rural" OR "Urban" OR "urbanization" OR bisexual* OR "Gay" OR Homophob* OR homosexual* OR "Lesbian" OR LGBT* OR "Men who have sex with men" OR "Queer" OR "sexual orientation" OR "Sexuality" OR "light at night" OR "night shift" OR overwork* OR "shift-work" OR "affluence" OR "classism" OR "Cost of Living" OR "debt" OR Depriv* OR "disadvantaged" OR "Economic Factors" OR "Economic Stability" OR "Economic Status" OR "financial resources" OR "homeless" OR "Homelessness" OR "Housing" OR "Housing Access" OR "Housing Stability" OR "impoverished" OR "Income" OR "Income inequality" OR "low income" OR "night-shift" OR "parental occupation" OR "Poor" OR "Poverty" OR "Poverty Areas" OR "Single Parent" OR "Single-Parent" OR "social class" OR "socially disadvantaged" OR "socioeconomic" OR "Socioeconomic Factors" OR "socioeconomic position" OR "Socioeconomic Status" OR unemploy* OR "Unemployment" OR "Unhoused People" OR "Wealth" OR "Wealth Distribution")	
Chemical	("Acenaphthene" OR "Acenaphthylene" OR "Acetone cyanohydrin" OR "Acetophenone" OR "Acrolein" OR "Acrylic acid" OR "Adiponitrile"	3,915,435

	OR "Aldrin" OR "Allyl alcohol" OR "Aluminum" OR "4-Amino-2,6-dinitrotoluene" OR "2-Amino-4,6-dinitrotoluene" OR "3-Amino-4-chlorobenzotrifluoride" OR "Aminodinitrotoluenes" OR "m-Aminophenol" OR "o-Aminophenol" OR "p-Aminophenol" OR "4-Aminopyridine" OR "2-Aminopyridine" OR "Ammonia" OR "Ammonium perchlorate" OR "Ammonium picrate" OR "Ammonium polyphosphate" OR "tert-Amyl alcohol" OR "tert-Amyl methyl ether" OR "Aniline" OR "Anilinobenzothiazole" OR "Anthracene" OR "9,10-Anthraquinone" OR "Antimony" OR "Antimony trioxide" OR "Aroclor 1254" OR "Aroclor 5460" OR "Azodicarbonamide" OR "Benzaldehyde" OR "Benzene" OR "Benzenethiol" OR "Benzo[e]pyrene" OR "Benzoic acid" OR "Benzothiazole" OR "Benzotrichloride" OR "Benzyl alcohol" OR "Benzyl chloride" OR "Bifenox" OR "1,1'-Biphenyl" OR "Boron Trichloride" OR "1-Bromo-2-chloroethane" OR "Bromobenzene" OR "Bromochloromethane" OR "Bromodichloromethane" OR "4-Bromodiphenyl ether" OR "p-Bromofluorobenzene" OR "Bromomethane" OR "2-Butanone" OR "sec-Butyl alcohol" OR "Butylated hydroxytoluene" OR "n-Butylbenzene" OR "sec-Butylbenzene" OR "tert-Butylbenzene" OR "Butyl benzyl phthalate" OR "tert-Butyl formate" OR "Calcium pyrophosphate" OR "Carbazole" OR "Chlordane" OR "Chlordecone" OR "4-Chloro-2-methylaniline" OR "4-Chloro-3-methylphenol" OR "2-Chloro-6-fluorophenol" OR "2-Chloroacetaldehyde" OR "Chloroacetic acid" OR "p-Chloroaniline" OR "Chlorobenzene" OR "p-Chlorobenzoic acid" OR "Chlorobenzoic acid, 2-" OR "4-Chlorobenzotrifluoride" OR "1-Chlorobutane" OR "2-Chlorobutane" OR "Chloroethane" OR "2-Chloroethanol" OR "Chloromethane" OR "2-Chloronaphthalene" OR "o-Chloronitrobenzene" OR "p-Chloronitrobenzene" OR "2-Chlorophenol" OR "2-Chloropropane" OR "o-Chlorotoluene" OR "p-Chlorotoluene" OR "Chrysene" OR "Cobalt" OR "aliphatic hydrocarbons" OR "Coronene" OR "Crotonaldehyde" OR "trans-Crotonaldehyde" OR "Cyanogen bromide" OR "Cyclohexane" OR "Cyclohexanone" OR "Cyclohexene" OR "Cyclosarin" OR "p-Cymene" OR "o,p'-DDD" OR "p,p'-DDD" OR "p,p'-DDE" OR "o,p'-DDT" OR "n-Decane" OR "Delta-Hexachlorocyclohexane" OR "2,5-diaminoToluene" OR "Diammonium phosphate" OR "Dibenzofuran" OR "Dibenzothiophene" OR "1,2-Dibromo-3-chloropropane" OR "1,3-Dibromobenzene" OR "Dibromochloromethane" OR "Dicalcium phosphate" OR "1,4-Dichloro-2-butene" OR "4,4'-Dichlorobenzophenone" OR "3,4-Dichlorobenzotrifluoride" OR "Dichlorodifluoromethane" OR "1,1-Dichloroethane" OR "1,2-Dichloroethane" OR "1,2-Dichloroethylene" OR "2,4-Dichlorophenol" OR "1,2-Dichloropropane" OR "1,3-Dichloropropane" OR "2,2-Dichloropropane" OR "Dicyclohexylamine" OR "Dicyclopentadiene" OR "Diethylene glycol monobutyl ether" OR "Diethylene glycol monomethyl ether" OR "Diethylformamide" OR "Diethyl phthalate" OR "Diisopropyl ether" OR "Diisopropyl methylphosphonate" OR "2,4-Dimethylaniline" OR "3,3'-Dimethylbenzidine" OR "Dimethylformamide" OR "1,1-Dimethylhydrazine" OR "Dimethylmercury" OR "Dimethyl methylphosphonate" OR "Dimethylphenethylamine" OR "2,4-Dimethylphenol" OR "Dimethyl Phthalate" OR "Dimethyl sulfide" OR	

	"Dimethylterephthalate" OR "1,2-Dinitrobenzene" OR "1,4-Dinitrobenzene" OR "1,3-Dinitrobenzene" OR "4,6-Dinitro-o-cresol" OR "2,4-Dinitrophenol" OR "2,6-Dinitrotoluene" OR "Di-n-octyl phthalate" OR "Dinoseb" OR "Diphenylamine" OR "Diphenyl ether" OR "Diphenyl sulfone" OR "Dipotassium phosphate" OR "Disodium phosphate" OR "Disulfoton" OR "Diundecyl phthalate" OR "Endosulfan" OR "Endosulfan sulfate" OR "Endrin" OR "Epichlorohydrin" OR "2-Ethoxyethanol" OR "Ethoxyethanol acetate" OR "Ethyl acetate" OR "Ethyl Acrylate" OR "Ethylbenzene" OR "Ethyl chloride" OR "Ethylene cyanohydrin" OR "Ethylene diamine" OR "Ethyl ether" OR "Ethyl methacrylate" OR "Ethylsarin" OR "Fluoranthene" OR "Fluorene" OR "Fluorobenzene" OR "2-Fluorobiphenyl" OR "2-Fluorophenol" OR "Formic acid" OR "Furan" OR "Gadolinium" OR "Glycidaldehyde" OR "Guanidine Compounds" OR "Guanidine nitrate" OR "n-Heptanal" OR "n-Heptane" OR "Hexachlorobenzene" OR "Hexachlorobutadiene" OR "Hexadecanoic acid" OR "Hexamethylphosphoramide" OR "Hexanedioic acid" OR "2-Hexanone" OR "HFC-143a" OR "Hydrazine" OR "Hydrogen sulfide" OR "Hydroquinone" OR "Indeno" OR "Inorganic phosphates" OR "Iodomethane" OR "Iron sulfide" OR "Isobutanol" OR "Isobutyl Alcohol" OR "Isopropanol" OR "p-Isopropyltoluene" OR "Lactonitrile" OR "Lanthanum" OR "Leptophos" OR "Lewisite" OR "Lithium" OR "Lithium perchlorate" OR "Lutetium" OR "Malononitrile" OR "Melamine" OR "2-Mercaptobenzothiazole" OR "Mercuric sulfide" OR "Methacrylonitrile" OR "Methoxychlor" OR "2-Methoxyethanol" OR "2-Methoxyethanol acetate" OR "4-Methyl-2-pentanol" OR "4-Methyl-2 Pentanone" OR "2-Methyl-5-nitroaniline" OR "Methyl acetate" OR "Methyl acrylate" OR "Methylcyclohexane" OR "n-Methylcyclohexylamine" OR "Methylcyclopentane" OR "4,4'-Methylenebis" OR "Methylene bromide" OR "Methyl hydrazine" OR "Methyl isobutyl ketone" OR "Methyl mercaptan" OR "1-Methylnaphthalene" OR "2-Methylnaphthalene" OR "2-Methylphenol" OR "3-Methylphenol" OR "4-Methylphenol" OR "Methyl phosphonic acid" OR "alpha-Methylstyrene" OR "Mirex" OR "Monoammonium phosphate" OR "Monocalcium phosphate" OR "Monopotassium phosphate" OR "Monosodium phosphate" OR "2-Naphthylamine" OR "Neodymium chloride" OR "N-Hexane" OR "Nicotinonitrile" OR "Nitroaniline, 2-" OR "Nitroaniline, 3-" OR "Nitroaniline, 4-" OR "Nitrocellulose" OR "2-Nitrodiphenylamine" OR "Nitrogen Mustard" OR "Nitroglycerin" OR "Nitroguanidine" OR "Nitromethane" OR "Nitrophenol, 2-" OR "p-Nitrophenol" OR "2-Nitropropane" OR "Nitrosodimethylamine, N-" OR "N-Methylaniline" OR "N,N-Dimethylcyclohexylamine" OR "cis-Nonachlor" OR "trans-Nonachlor" OR "Nonane, n-" OR "Octadecanoic acid" OR "Orthocresol" OR "PBDEs" OR "Pentachlorobenzene" OR "Pentachloroethane" OR "Pentaerythritol tetranitrate" OR "Pentamethyl dipropylenetriamine" OR "Pentamethylphosphoramide" OR "Perfluorobutane Sulfonate" OR "Perfluorobutyrate" OR "Perylene" OR "Phenanthrene" OR "Phenothiazine" OR "1-Phenyl-1-" OR "Phenylenediamine, o-" OR "Phenyl Isothiocyanate" OR "Phenylphenol 2-" OR "Phosphoric Acid" OR "Phosphorus pentoxide" OR "Phosphorus, white" OR "m-Phthalic	

	acid" OR "o-Phthalic acid" OR "p-Phthalic Acid" OR "Picramic acid" OR "Picric acid" OR "Piperidine" OR "Polychlorinated terphenyls" OR "Polyphosphoric acid" OR "Potassium Perchlorate" OR "Potassium Perfluorobutane Sulfonate" OR "Potassium tripolyphosphate" OR "Praseodymium chloride" OR "Promethium" OR "2-Propanol" OR "2-Propanone" OR "n-Propyl alcohol" OR "n-Propylbenzene" OR "Propylene glycol" OR "Pyrene" OR "2-bromo-Pyridine" OR "Rubidium" OR "Samarium chloride" OR "Samarium nitrate" OR "SAN Trimer" OR "Sarin" OR "Selenium" OR "Selenourea" OR "Sodium acid pyrophosphate" OR "Sodium aluminum phosphate" OR "Sodium hexametaphosphate" OR "Sodium perchlorate" OR "Sodium polyphosphate" OR "Sodium trimetaphosphate" OR "Sodium tripolyphosphate" OR "Soman" OR "Strontium" OR "Sulfolane" OR "1,1'-Sulfonylbis" OR "Tabun" OR "Terbium" OR "1,2,4,5-Tetrachlorobenzene" OR "1,1,1,2-Tetrachloroethane" OR "1,1,2,2-Tetrachloroethane" OR "Tetrahydrothiophene" OR "Tetramethylcyclohexane" OR "Tetrasodium pyrophosphate" OR "Thiocyanate" OR "Thiocyanic Acid" OR "Thiodiglycol" OR "Thiophene" OR "Thulium" OR "Titanium" OR "Titanium dioxide" OR "Toluene" OR "3,4-Toluenediamine" OR "p-Toluidine" OR "Toxaphene" OR "Triacetin" OR "1,2,4-Tribromobenzene" OR "2,4,6-Tribromophenol" OR "Tributyl phosphate" OR "Tri-Butyltin compounds" OR "Tricaine methanesulfonate" OR "Tricalcium phosphate" OR "2,4,6-Trichloroaniline" OR "1,2,3-Trichlorobenzene" OR "1,2,4-Trichlorobenzene" OR "1,1,2-Trichloroethane" OR "Trichlorofluoromethane" OR "2,4,5-Trichlorophenol" OR "2,4,6-Trichlorophenol" OR "2,4,5-Trichlorophenoxyacetic acid" OR "1,1,2-Trichloropropane" OR "1,2,3-Trichloropropene" OR "2,3,6-Trichlorotoluene" OR "alpha-2,6-Trichlorotoluene" OR "Tridymite" OR "Triethylene glycol" OR "1,1,1-Trifluoroethane" OR "Trimagnesium phosphate" OR "Trimellitic anhydride" OR "1,2,3-Trimethylbenzene" OR "1,2,4-Trimethylbenzene" OR "1,3,5-Trimethylbenzene" OR "2,2,4-Trimethylpentane" OR "2,4,4-Trimethylpentene" OR "Trimethyl phosphate" OR "1,3,5-Trinitrobenzene" OR "Trinitrophenylmethylnitramine" OR "2,4,6-Trinitrotoluene" OR "Triphenylphosphine oxide" OR "Tripoli" OR "Tripotassium phosphate" OR "Trisodium phosphate" OR "Tungsten" OR "Vanadium" OR "Vanadium pentoxide" OR "Vinyl bromide" OR "Xylene" OR "Ytterbium" OR "Zirconium" OR "Acephate" OR "Acetaldehyde" OR "Acetochlor" OR "Acetone" OR "Acetonitrile" OR "Acetophenone" OR "Acetyl chloride" OR "Acifluorfen, sodium" OR "Acrolein" OR "Acrylamide" OR "Acrylic acid" OR "Acrylonitrile" OR "Adiponitrile" OR "Alachlor" OR "Alar" OR "Aldicarb" OR "Aldicarb sulfone" OR "Aldrin" OR "Ally" OR "Allyl alcohol" OR "Allyl chloride" OR "Aluminum phosphide" OR "Amdro" OR "Ametryn" OR "Aminopyridine" OR "Amitraz" OR "Ammonia" OR "Ammonium acetate" OR "Ammonium methacrylate" OR "Ammonium sulfamate" OR "Aniline" OR "Anisidine" OR "Anthracene" OR "Antimony" OR "Antimony trioxide" OR "Apollo" OR "Aramite" OR "Aroclor 1016" OR "Aroclor 1248" OR "Aroclor 1254" OR "Arsenic, Inorganic" OR "Arsine" OR "Asbestos" OR "Assure" OR "Asulam" OR "Atrazine" OR	

	"Avermectin B1" OR "Azobenzene" OR "Barium" OR "Baygon" OR "Bayleton" OR "Baythroid" OR "Benefin" OR "Benomyl" OR "Bentazon" OR "Benz[a]anthracene" OR "Benzaldehyde" OR "Benzene" OR "Benzidine" OR "Benzo[a]pyrene" OR "Benzo[b]fluoranthene" OR "Benzo[g,h,i]perylene" OR "Benzo[k]fluoranthene" OR "Benzoic acid" OR "Benzotrichloride" OR "Benzyl chloride" OR "Beryllium" OR "Bidrin" OR "Biphenthrin" OR "Biphenyl" OR "Bis(2-chloro-1-methylethyl) ether" OR "Bis(2-chloroethoxy)methane" OR "Bis(chloroethyl)ether" OR "Bis(chloromethyl)ether" OR "Bisphenol A" OR "Boron" OR "Bromate" OR "Brominated dibenzofurans" OR "Bromobenzene" OR "Bromochloromethane" OR "Bromodichloromethane" OR "Bromodiphenyl ether" OR "Bromoform" OR "Bromomethane" OR "Bromotrichloromethane" OR "Bromoxynil" OR "Bromoxynil octanoate" OR "Butadiene" OR "n-Butanol" OR "tert-Butyl Alcohol" OR "Butyl benzyl phthalate" OR "Butylate" OR "Butylchloride" OR "Cacodylic acid" OR "Cadmium" OR "Calcium cyanide" OR "Caprolactam" OR "Captafol" OR "Captan" OR "Carbaryl" OR "Carbofuran" OR "Carbon disulfide" OR "Carbon tetrachloride" OR "Carbonyl sulfide" OR "Carbosulfan" OR "Carboxin" OR "Cerium Oxide" OR "Chloral hydrate" OR "Chloramben" OR "Chlordane" OR "Chlordecone" OR "Chlorimuron-ethyl" OR "Chlorine" OR "Chlorine dioxide" OR "Chlorite" OR "Chloro-1,1-difluoroethane" OR "Chloroacetophenone" OR "Chloroaniline" OR "Chlorobenzene" OR "Chlorobenzilate" OR "Chlorobutane" OR "Chlorocyclopentadiene" OR "Chlorodifluoromethane" OR "Chloroform" OR "Chloromethyl methyl ether" OR "Chloronaphthalene" OR "Chlorophenol" OR "Chlorophenyl methyl sulfide" OR "Chlorophenyl methyl sulfone" OR "Chloroprene" OR "Chlorothalonil" OR "Chlorotoluene" OR "Chlorpropham" OR "Chlorpyrifos" OR "Chlorsulfuron" OR "Chromium(VI)" OR "Chrysene" OR "Cobalt" OR "Coke oven emissions" OR "Copper" OR "Copper cyanide" OR "Creosote" OR "Crotonaldehyde" OR "Cumene" OR "Cyanazine" OR "Cyanide, free" OR "Cyanogen" OR "Cyanogen bromide" OR "Cyclohexane" OR "Cyclohexanone" OR "Cyclohexylamine" OR "Cyhalothrin/Karate" OR "Cypermethrin" OR "Cyromazine" OR "Dacthal" OR "Danitol" OR "Decabromodiphenyl ether" OR "Demeton" OR "Diaminotoluene" OR "Diazomethane" OR "Dibenz[a,h]anthracene" OR "Dibenzofuran" OR "Dibromo-3-chloropropane" OR "Dibromobenzene" OR "Dibromochloromethane" OR "Dibromodichloromethane" OR "Dibromodiphenyl ether" OR "Dibromoethane" OR "Dibutyl phthalate" OR "Dicamba" OR "Dichloroacetic acid" OR "Dichlorobenzene" OR "Dichlorobenzidine" OR "Dichlorodifluoromethane" OR "Dichlorodiphenyl dichloroethane" OR "Dichlorodiphenyldichloroethylene" OR "Dichlorodiphenyltrichloroethane" OR "Dichloroethane" OR "Dichloroethylene" OR "Dichloromethane" OR "Dichlorophenol" OR "Dichlorophenoxy)butyric acid" OR "Dichlorophenoxyacetic acid" OR "Dichloropropane" OR "Dichloropropanol" OR "Dichloropropene" OR "Dichlorvos" OR "Dicofol" OR "Dieldrin" OR "Diesel engine exhaust" OR "Diethyl sulfate" OR "Diethyl-p-nitrophenylphosphate" OR "Diethylene glycol dinitrate" OR "Di(2-ethylhexyl)adipate" OR "Di (2-	

	ethylhexyl)phthalate" OR "Difenzoquat" OR "Diflubenzuron" OR "Difluoroethane" OR "Diisobutyl phthalate" OR "Diisononyl phthalate" OR "Diisopropyl methylphosphonate" OR "Dimethipin" OR "Dimethoate" OR "Dimethyl phthalate" OR "Dimethyl sulfate" OR "Dimethyl terephthalate" OR "Dimethylamine" OR "Dimethylaniline" OR "Dimethylbenzidine" OR "Dimethylformamide" OR "Dimethylphenol" OR "Dinitro-o-cyclohexyl phenol" OR "Dinitrobenzene" OR "Dinitrophenol" OR "Dinitrotoluene" OR "Dinoseb" OR "Dioxane" OR "Diphenamid" OR "Diphenylamine" OR "Diphenylhydrazine" OR "Diquat" OR "Disulfoton" OR "Dithiane" OR "Diuron" OR "Dodine" OR "Endosulfan" OR "Endothall" OR "Endrin" OR "Epichlorohydrin" OR "Epoxybutane" OR "Ethephon" OR "Ethion" OR "Ethoxyethanol" OR "Ethyl acetate" OR "Ethyl carbamate" OR "Ethyl chloride" OR "Ethyl dipropylthiocarbamate" OR "Ethyl ether" OR "Ethyl Tertiary Butyl Ether" OR "Ethylbenzene" OR "Ethylene diamine" OR "Ethylene glycol" OR "Ethylene glycol monobutyl ether" OR "Ethylene oxide" OR "Ethylene thiourea" OR "Ethyleneimine" OR "Express" OR "Fenamiphos" OR "Fluometuron" OR "Fluoranthene" OR "Fluorene" OR "Fluorine" OR "Fluridone" OR "Flurprimidol" OR "Flutolanil" OR "Fluvalinate" OR "Folpet" OR "Fomesafen" OR "Fonofos" OR "Formaldehyde" OR "Formic acid" OR "Fosetyl-al" OR "Furan" OR "Furfural" OR "Furmecyclox" OR "Glufosinate-ammonium" OR "Glycidaldehyde" OR "Glyphosate" OR "Haloxyfop-methyl" OR "Harmony" OR "Heptachlor" OR "Heptachlor epoxide" OR "Heptane" OR "Hexabromobenzene" OR "Hexabromocyclododecane" OR "Hexabromodiphenyl ether" OR "Hexachlorobenzene" OR "Hexachlorobutadiene" OR "Hexachlorocyclohexane" OR "Hexachlorocyclopentadiene" OR "Hexachlorodibenzo-p-dioxin" OR "Hexachloroethane" OR "Hexachlorophene" OR "Hexahydro-1,3,5-trinitro-1,3,5-triazine" OR "Hexamethylene diisocyanate" OR "Hexane" OR "Hexanone" OR "Hexazinone" OR "Hydrogen chloride" OR "Hydrogen Cyanide" OR "Cyanide Salts" OR "Hydrogen sulfide" OR "Hydroquinone" OR "Imazalil" OR "Imazaquin" OR "Indeno[1,2,3-cd]pyrene" OR "Iprodione" OR "Isobutyl alcohol" OR "Isophorone" OR "Isopropalin" OR "Isopropyl methyl phosphonic acid" OR "Isoxaben" OR "Lactofen" OR "Lead" OR "Libby Amphibole Asbestos" OR "Limonene" OR "Linuron" OR "Londax" OR "Malathion" OR "Maleic anhydride" OR "Maleic hydrazide" OR "Maneb" OR "Manganese" OR "Mepiquat chloride" OR "Mercuric chloride" OR "Mercury" OR "Merphos" OR "Metalaxyl" OR "Methacrylonitrile" OR "Methamidophos" OR "Methanol" OR "Methidathion" OR "Methomyl" OR "Methoxychlor" OR "Methoxyethanol" OR "Methyl chloride" OR "Methyl ethyl ketone" OR "Methyl iodide" OR "Methyl isobutyl ketone" OR "Methyl isocyanate" OR "Methyl methacrylate" OR "Methyl parathion" OR "Methyl tert-butyl ether" OR "Methyl-4-chlorophenoxyacetic acid" OR "Methylene Diphenyl Diisocyanate" OR "monomeric MDI" OR "polymeric MDI" OR "Methylmercury" OR "Methylnaphthalene" OR "Methylphenol" OR "Metolachlor" OR "Metribuzin" OR "Mirex" OR "Molinate" OR "Molybdenum" OR "Monochloramine" OR "Naled" OR "Naphthalene" OR "Napropamide" OR "Nickel carbonyl" OR "Nickel refinery dust" OR "Nickel	

	subsulfide" OR "Nickel" OR "Nitrapyrin" OR "Nitrate" OR "Nitric oxide" OR "Nitrite" OR "Nitrobenzene" OR "Nitrogen dioxide" OR "Nitroguanidine" OR "Nitrophenol" OR "Nitropropane" OR "Nitrosodi-N-propylamine" OR "Nitrosodiethanolamine" OR "Nitrosodiethylamine" OR "Nitrosodimethylamine" OR "Nitrosodiphenylamine" OR "Nitrosopyrrolidine" OR "Nonabromodiphenyl ether" OR "Norflurazon" OR "NuStar" OR "Octabromodiphenyl ether" OR "Octahydro-1,3,5,7-tetranitro-1,3,5,7-tetrazocine" OR "Oryzalin" OR "Oxadiazon" OR "Oxamyl" OR "Oxyfluorfen" OR "Paclobutrazol" OR "Paraquat" OR "Parathion" OR "Pendimethalin" OR "Pentabromodiphenyl ether" OR "Pentachlorobenzene" OR "Pentachloronitrobenzene" OR "Pentachlorophenol" OR "Pentafluoroethane" OR "Perchlorate" OR "Perfluorobutanoic Acid" OR "Perfluorodecanoic Acid" OR "Perfluorohexanesulfonic Acid" OR "Perfluorohexanoic Acid" OR "Permethrin" OR "Phenanthrene" OR "Phenmedipham" OR "Phenol" OR "Phenylenediamine" OR "Phenylmercuric acetate" OR "Phosalone" OR "Phosgene" OR "Phosmet" OR "Phosphine" OR "Phosphoric acid" OR "Phthalic anhydride" OR "Picloram" OR "Pirimiphos-methyl" OR "Polychlorinated Biphenyls" OR "Polycyclic aromatic hydrocarbon" OR "Potassium cyanide" OR "Potassium silver cyanide" OR "Prochloraz" OR "Prometon" OR "Prometryn" OR "Pronamide" OR "Propachlor" OR "Propanil" OR "Propargite" OR "Propargyl alcohol" OR "Propazine" OR "Propham" OR "Propiconazole" OR "Propiolactone" OR "Propionaldehyde" OR "Propylene glycol" OR "Propylene glycol monomethyl ether" OR "Propylene oxide" OR "Propyleneimine" OR "Pursuit" OR "Pydrin" OR "Pyrene" OR "Pyridine" OR "Quinalphos" OR "Quinoline" OR "Quinone" OR "Radium 228" OR "Radium 226" OR "Radon 222" OR "Refractory ceramic fibers" OR "Resmethrin" OR "Rotenone" OR "Savey" OR "Selenious acid" OR "Selenium sulfide" OR "Selenourea" OR "Sethoxydim" OR "Silver" OR "Silver cyanide" OR "Simazine" OR "Sodium azide" OR "Sodium cyanide" OR "Sodium diethyldithiocarbamate" OR "Sodium fluoroacetate" OR "Strontium" OR "Strychnine" OR "Styrene" OR "Systhane" OR "Tebuthiuron" OR "Terbacil" OR "Terbutryn" OR "Tetrabromodiphenyl ether" OR "Tetrachlorobenzene" OR "Tetrachlorocyclopentadiene" OR "Tetrachlorodibenzo-p-dioxin" OR "Tetrachloroethane" OR "Tetrachloroethylene" OR "Tetrachlorophenol" OR "Tetrachlorovinphos" OR "Tetraethyl lead" OR "Tetraethyldithiopyrophosphate" OR "Tetrafluoroethane" OR "Tetrahydrofuran" OR "Thallium" OR "Thallium acetate" OR "Thallium carbonate" OR "Thallium chloride" OR "Thallium nitrate" OR "Thallium oxide" OR "Thallium(I) sulfate" OR "Thiobencarb" OR "Thiophanate-methyl" OR "Thiram" OR "Toluene" OR "Toluene diisocyanate" OR "Toxaphene" OR "Tralomethrin" OR "Triallate" OR "Triasulfuron" OR "Tribromobenzene" OR "Tribromodiphenyl ether" OR "Tributyltin oxide" OR "Trichloro-1,2,2-trifluoroethane" OR "Trichloroacetic acid" OR "Trichlorobenzene" OR "Trichlorocyclopentadiene" OR "Trichloroethane" OR "Trichloroethylene" OR "Trichlorofluoromethane" OR "Trichlorophenol" OR "Trichlorophenoxy) propionic acid" OR "Trichlorophenoxyacetic acid" OR "Trichloropropane" OR "Tricresol"	

	OR "Tridiphane" OR "Triethylamine" OR "Triethylene glycol monoethyl ether" OR "Trifluralin" OR "Trimethylbenzene" OR "Trimethylpentane" OR "Trinitrobenzene" OR "Trinitrotoluene" OR "Uranium" OR "Urea" OR "Vanadium pentoxide" OR "Vernam" OR "Vinclozolin" OR "Vinyl acetate" OR "Vinyl bromide" OR "Vinyl chloride" OR "Warfarin" OR "White phosphorus" OR "Xylenes" OR "Zinc" OR "Zinc cyanide" OR "Zinc phosphide" OR "Zineb" OR "environmental exposure" OR "environmental pollutant" OR Pesticide* OR "Flame Retardant" OR "Flame Retardants" OR "Fire Retardant" OR "Fire Retardants" OR "PFAS" OR "PFOS" OR "polyfluoroalkyl substances" OR "perfluoroalkyl substances" OR "polyfluoroalkyl substance" OR "perfluoroalkyl substance" OR Organophosphate* OR "Polycyclic Aromatic Hydrocarbon" OR "Polycyclic Aromatic Hydrocarbons" OR "Endocrine Disruptor" OR "Endocrine Disruptors" OR Paraben* OR Phenol* OR Bisphenol* OR Phthalate* OR "Traffic Related Air Pollution" OR "Particulate Matter")	
CRA	(cumulative OR joint OR effect modification OR mediation OR effect modifier OR mediator)	3,091,940
Reviews	(“Systematic Review*” OR “Scoping Review*” OR “Systematic Evidence Map*”)	372,074
**Combined**	Susceptibility AND Chemical AND CRA AND Reviews	2,523

Web of Science

Group	String	Results
Susceptibility	(Activit* OR Addict* OR "addiction" OR "alcohol user" OR alcohol* OR Behavior* OR Behaviour* OR Choice* OR "Diet" OR "drug addict" OR "drug user" OR Exercis* OR Lifestyle* OR "physical activity" OR "physical inactivity" OR "Sedentary" OR smoker* OR "Substance Abuse" OR "adverse childhood events" OR "adverse childhood experience" OR "Adverse childhood experiences" OR "Bullying" OR "Childhood Stress" OR "crime" OR "Foster Care" OR "harassment" OR "household dysfunction" OR "intergenerational trauma" OR "trauma" OR victim* OR "violence" OR "allostatic load" OR crimin* OR Imprison* OR Incarcerat* OR "parental incarceration" OR "Probation" OR "co-exposure" OR "cumulative" OR "exposome" OR Metal* OR mixture* OR "nutrient" OR Pesticide* OR phthalate* OR "ableism" OR Amputee* OR "Blind" OR "Deaf" OR disab* OR handicap* OR Hearing Impair* OR Parapleg* OR Visual Impair* OR Visually Impair* OR "wheelchair" OR "academic achievement" OR "college education" OR "college level" OR "Education Access" OR "Education level" OR "educational achievement" OR "Educational Attainment" OR "English language proficiency" OR "Graduation Status" OR "highest degree" OR "Illiteracy" OR "linguistic competency" OR mentor* OR "Parental education attainment" OR "Quality of Education" OR Epigenetic* OR Food bank* OR "Food Desert" OR "food insecurity" OR "food security" OR "Food stamps" OR "hunger" OR School lunch* OR SNAP Benefit* OR "boys" OR "female" OR "Gender identity" OR "Genderqueer" OR "girls" OR "Intersex" OR "male" OR "nonbinary" OR "non-binary" OR	29,960,089

	"Gender Minority" OR "Gender Minorities" OR "sex" OR "transgender" OR "transphobia" OR "adverse outcome pathway" OR "adverse outcome pathways" OR "gene-environment interaction" OR genetic* OR genotype* OR polymorphism* OR "variability" OR variat* OR "access to healthcare" OR "Health Care Access" OR Health Care Cost* OR Health Disparit* OR "health education" OR "Health Insurance" OR "Health literacy" OR "Health Risk Behavior" OR "health warnings" OR hospital closure* OR "Insurance Access" OR "Insurance Coverage" OR "Lack of Care" OR "Quality of Health Care" OR "Underinsured" OR "Uninsured" OR Adoles* OR Adult* OR Child* OR Elder* OR Infan* OR "Senior Citizen" OR Teenage* OR "family dysfunction" OR "guardian" OR "maternal anxiety" OR Maternal* OR "ADHD" OR Adjustment Disorder* OR "Anorexia" OR "Anxiety" OR "Anxious" OR Attention Deficit* OR "Bipolar" OR "Bulimia" OR Depress* OR Dissociat* OR "Dissociative Identity Disorder" OR Eating Disorder* OR "Gender Dysphoria" OR Learning Disab* OR Mental Disorder* OR "Mental Health" OR "Neurotic" OR Schizo* OR Stress* OR Neurodivers* OR "Neurodivergent" OR "access to sport and recreation" OR "accessibility" OR "Clean Water" OR "crowded household" OR "energy networks" OR "Garbage" OR "green space" OR "greenspace" OR "group quarters" OR Highway* OR Housing Cost* OR "Housing Density" OR "infrastructure" OR "Internet Access" OR "landfill" OR "Mobile home" OR "Mobile homes" OR "neighborhood" OR "neighborhood deprivation" OR "open space" OR "Project building" OR "Public Housing" OR Public Park* OR "Public transport" OR "Quality of Housing" OR "residence characteristics" OR "residence characteristics" OR "Residential characteristics" OR "underserved" OR Walkab* OR "waste management" OR "waste treatment" OR Water Contam* OR "water supply" OR associated factor* OR related factor* OR endotoxin* OR Environment* OR "infectious agents" OR "microbial contaminants" OR "nonchemical" OR "non-chemical" OR "vector borne diseases" OR "vector-borne diseases" OR "waterborne pathogens" OR Pregnan* OR veteran* OR "Vulnerable population" OR "climate" OR "cold" OR "heat" OR "heatwave" OR "humidity" OR "noise" OR "temperature" OR allerg* OR "comorbidity" OR "morbidity" OR "multimorbidity" OR "pre-existing" OR "acculturation" OR "acculturative stress" OR African American* OR Alaska Native* OR Alaskan Native* OR American Indian* OR Asian* OR Black* OR "Caucasian" OR "colonization" OR Cultural Bias* OR discriminat* OR "Ethnicity" OR "Hispanic" OR "Immigration Status" OR "Indigenous" OR Latina* OR Latino* OR "Latinx" OR "marginalized" OR "migration status" OR Minorit* OR "minorities" OR "Minority" OR Native American* OR Native Hawaiian* OR "Oppression" OR Pacific Islander* OR "People of Color" OR "Privilege" OR "race" OR "racism" OR "Segregation" OR subpopulation* OR "Under represented groups" OR White* OR "Racialized" OR "metro" OR "nonmetro" OR "Rural" OR "Urban" OR "urbanization" OR bisexual* OR "Gay" OR Homophob* OR homosexual* OR "Lesbian" OR LGBT* OR "Men who have sex with men" OR "Queer" OR "sexual orientation" OR "Sexuality" OR "light at night" OR "night shift" OR overwork* OR "shift-work" OR "affluence" OR "classism" OR "Cost of Living" OR	

	"debt" OR Depriv* OR "disadvantaged" OR "Economic Factors" OR "Economic Stability" OR "Economic Status" OR "financial resources" OR "homeless" OR "Homelessness" OR "Housing" OR "Housing Access" OR "Housing Stability" OR "impoverished" OR "Income" OR "Income inequality" OR "low income" OR "night-shift" OR "parental occupation" OR "Poor" OR "Poverty" OR "Poverty Areas" OR "Single Parent" OR "Single-Parent" OR "social class" OR "socially disadvantaged" OR "socioeconomic" OR "Socioeconomic Factors" OR "socioeconomic position" OR "Socioeconomic Status" OR unemploy* OR "Unemployment" OR "Unhoused People" OR "Wealth" OR "Wealth Distribution")	
Chemical	("Acenaphthene" OR "Acenaphthylene" OR "Acetone cyanohydrin" OR "Acetophenone" OR "Acrolein" OR "Acrylic acid" OR "Adiponitrile" OR "Aldrin" OR "Allyl alcohol" OR "Aluminum" OR "4-Amino-2,6-dinitrotoluene" OR "2-Amino-4,6-dinitrotoluene" OR "3-Amino-4-chlorobenzotrifluoride" OR "Aminodinitrotoluenes" OR "m-Aminophenol" OR "o-Aminophenol" OR "p-Aminophenol" OR "4-Aminopyridine" OR "2-Aminopyridine" OR "Ammonia" OR "Ammonium perchlorate" OR "Ammonium picrate" OR "Ammonium polyphosphate" OR "tert-Amyl alcohol" OR "tert-Amyl methyl ether" OR "Aniline" OR "Anilinobenzothiazole" OR "Anthracene" OR "9,10-Anthraquinone" OR "Antimony" OR "Antimony trioxide" OR "Aroclor 1254" OR "Aroclor 5460" OR "Azodicarbonamide" OR "Benzaldehyde" OR "Benzene" OR "Benzenethiol" OR "Benzo[e]pyrene" OR "Benzoic acid" OR "Benzothiazole" OR "Benzotrichloride" OR "Benzyl alcohol" OR "Benzyl chloride" OR "Bifenox" OR "1,1'-Biphenyl" OR "Boron Trichloride" OR "1-Bromo-2-chloroethane" OR "Bromobenzene" OR "Bromochloromethane" OR "Bromodichloromethane" OR "4-Bromodiphenyl ether" OR "p-Bromofluorobenzene" OR "Bromomethane" OR "2-Butanone" OR "sec-Butyl alcohol" OR "Butylated hydroxytoluene" OR "n-Butylbenzene" OR "sec-Butylbenzene" OR "tert-Butylbenzene" OR "Butyl benzyl phthalate" OR "tert-Butyl formate" OR "Calcium pyrophosphate" OR "Carbazole" OR "Chlordane" OR "Chlordecone" OR "4-Chloro-2-methylaniline" OR "4-Chloro-3-methylphenol" OR "2-Chloro-6-fluorophenol" OR "2-Chloroacetaldehyde" OR "Chloroacetic acid" OR "p-Chloroaniline" OR "Chlorobenzene" OR "p-Chlorobenzoic acid" OR "Chlorobenzoic acid, 2-" OR "4-Chlorobenzotrifluoride" OR "1-Chlorobutane" OR "2-Chlorobutane" OR "Chloroethane" OR "2-Chloroethanol" OR "Chloromethane" OR "2-Chloronaphthalene" OR "o-Chloronitrobenzene" OR "p-Chloronitrobenzene" OR "2-Chlorophenol" OR "2-Chloropropane" OR "o-Chlorotoluene" OR "p-Chlorotoluene" OR "Chrysene" OR "Cobalt" OR "aliphatic hydrocarbons" OR "Coronene" OR "Crotonaldehyde" OR "trans-Crotonaldehyde" OR "Cyanogen bromide" OR "Cyclohexane" OR "Cyclohexanone" OR "Cyclohexene" OR "Cyclosarin" OR "p-Cymene" OR "o,p'-DDD" OR "p,p'-DDD" OR "p,p'-DDE" OR "o,p'-DDT" OR "n-Decane" OR "Delta-Hexachlorocyclohexane" OR "2,5-diaminoToluene" OR "Diammonium phosphate" OR "Dibenzofuran" OR "Dibenzothiophene" OR "1,2-Dibromo-3-chloropropane" OR "1,3-Dibromobenzene" OR "Dibromochloromethane" OR "Dicalcium phosphate" OR "1,4-	7,587,739

	Dichloro-2-butene" OR "4,4'-Dichlorobenzophenone" OR "3,4-Dichlorobenzotrifluoride" OR "Dichlorodifluoromethane" OR "1,1-Dichloroethane" OR "1,2-Dichloroethane" OR "1,2-Dichloroethylene" OR "2,4-Dichlorophenol" OR "1,2-Dichloropropane" OR "1,3-Dichloropropane" OR "2,2-Dichloropropane" OR "Dicyclohexylamine" OR "Dicyclopentadiene" OR "Diethylene glycol monobutyl ether" OR "Diethylene glycol monomethyl ether" OR "Diethylformamide" OR "Diethyl phthalate" OR "Diisopropyl ether" OR "Diisopropyl methylphosphonate" OR "2,4-Dimethylaniline" OR "3,3'-Dimethylbenzidine" OR "Dimethylformamide" OR "1,1-Dimethylhydrazine" OR "Dimethylmercury" OR "Dimethyl methylphosphonate" OR "Dimethylphenethylamine" OR "2,4-Dimethylphenol" OR "Dimethyl Phthalate" OR "Dimethyl sulfide" OR "Dimethylterephthalate" OR "1,2-Dinitrobenzene" OR "1,4-Dinitrobenzene" OR "1,3-Dinitrobenzene" OR "4,6-Dinitro-o-cresol" OR "2,4-Dinitrophenol" OR "2,6-Dinitrotoluene" OR "Di-n-octyl phthalate" OR "Dinoseb" OR "Diphenylamine" OR "Diphenyl ether" OR "Diphenyl sulfone" OR "Dipotassium phosphate" OR "Disodium phosphate" OR "Disulfoton" OR "Diundecyl phthalate" OR "Endosulfan" OR "Endosulfan sulfate" OR "Endrin" OR "Epichlorohydrin" OR "2-Ethoxyethanol" OR "Ethoxyethanol acetate" OR "Ethyl acetate" OR "Ethyl Acrylate" OR "Ethylbenzene" OR "Ethyl chloride" OR "Ethylene cyanohydrin" OR "Ethylene diamine" OR "Ethyl ether" OR "Ethyl methacrylate" OR "Ethylsarin" OR "Fluoranthene" OR "Fluorene" OR "Fluorobenzene" OR "2-Fluorobiphenyl" OR "2-Fluorophenol" OR "Formic acid" OR "Furan" OR "Gadolinium" OR "Glycidaldehyde" OR "Guanidine Compounds" OR "Guanidine nitrate" OR "n-Heptanal" OR "n-Heptane" OR "Hexachlorobenzene" OR "Hexachlorobutadiene" OR "Hexadecanoic acid" OR "Hexamethylphosphoramide" OR "Hexanedioic acid" OR "2-Hexanone" OR "HFC-143a" OR "Hydrazine" OR "Hydrogen sulfide" OR "Hydroquinone" OR "Indeno" OR "Inorganic phosphates" OR "Iodomethane" OR "Iron sulfide" OR "Isobutanol" OR "Isobutyl Alcohol" OR "Isopropanol" OR "p-Isopropyltoluene" OR "Lactonitrile" OR "Lanthanum" OR "Leptophos" OR "Lewisite" OR "Lithium" OR "Lithium perchlorate" OR "Lutetium" OR "Malononitrile" OR "Melamine" OR "2-Mercaptobenzothiazole" OR "Mercuric sulfide" OR "Methacrylonitrile" OR "Methoxychlor" OR "2-Methoxyethanol" OR "2-Methoxyethanol acetate" OR "4-Methyl-2-pentanol" OR "4-Methyl-2 Pentanone" OR "2-Methyl-5-nitroaniline" OR "Methyl acetate" OR "Methyl acrylate" OR "Methylcyclohexane" OR "n-Methylcyclohexylamine" OR "Methylcyclopentane" OR "4,4'-Methylenebis" OR "Methylene bromide" OR "Methyl hydrazine" OR "Methyl isobutyl ketone" OR "Methyl mercaptan" OR "1-Methylnaphthalene" OR "2-Methylnaphthalene" OR "2-Methylphenol" OR "3-Methylphenol" OR "4-Methylphenol" OR "Methyl phosphonic acid" OR "alpha-Methylstyrene" OR "Mirex" OR "Monoammonium phosphate" OR "Monocalcium phosphate" OR "Monopotassium phosphate" OR "Monosodium phosphate" OR "2-Naphthylamine" OR "Neodymium chloride" OR "N-Hexane" OR "Nicotinonitrile" OR "Nitroaniline, 2-" OR "Nitroaniline, 3-" OR "Nitroaniline, 4-" OR	

	"Nitrocellulose" OR "2-Nitrodiphenylamine" OR "Nitrogen Mustard" OR "Nitroglycerin" OR "Nitroguanidine" OR "Nitromethane" OR "Nitrophenol, 2-" OR "p-Nitrophenol" OR "2-Nitropropane" OR "Nitrosodimethylamine, N-" OR "N-Methylaniline" OR "N,N-Dimethylcyclohexylamine" OR "cis-Nonachlor" OR "trans-Nonachlor" OR "Nonane, n-" OR "Octadecanoic acid" OR "Orthocresol" OR "PBDEs" OR "Pentachlorobenzene" OR "Pentachloroethane" OR "Pentaerythritol tetranitrate" OR "Pentamethyl dipropylenetriamine" OR "Pentamethylphosphoramide" OR "Perfluorobutane Sulfonate" OR "Perfluorobutyrate" OR "Perylene" OR "Phenanthrene" OR "Phenothiazine" OR "1-Phenyl-1-" OR "Phenylenediamine, o-" OR "Phenyl Isothiocyanate" OR "Phenylphenol 2-" OR "Phosphoric Acid" OR "Phosphorus pentoxide" OR "Phosphorus, white" OR "m-Phthalic acid" OR "o-Phthalic acid" OR "p-Phthalic Acid" OR "Picramic acid" OR "Picric acid" OR "Piperidine" OR "Polychlorinated terphenyls" OR "Polyphosphoric acid" OR "Potassium Perchlorate" OR "Potassium Perfluorobutane Sulfonate" OR "Potassium tripolyphosphate" OR "Praseodymium chloride" OR "Promethium" OR "2-Propanol" OR "2-Propanone" OR "n-Propyl alcohol" OR "n-Propylbenzene" OR "Propylene glycol" OR "Pyrene" OR "2-bromo-Pyridine" OR "Rubidium" OR "Samarium chloride" OR "Samarium nitrate" OR "SAN Trimer" OR "Sarin" OR "Selenium" OR "Selenourea" OR "Sodium acid pyrophosphate" OR "Sodium aluminum phosphate" OR "Sodium hexametaphosphate" OR "Sodium perchlorate" OR "Sodium polyphosphate" OR "Sodium trimetaphosphate" OR "Sodium tripolyphosphate" OR "Soman" OR "Strontium" OR "Sulfolane" OR "1,1'-Sulfonylbis" OR "Tabun" OR "Terbium" OR "1,2,4,5-Tetrachlorobenzene" OR "1,1,1,2-Tetrachloroethane" OR "1,1,2,2-Tetrachloroethane" OR "Tetrahydrothiophene" OR "Tetramethylcyclohexane" OR "Tetrasodium pyrophosphate" OR "Thiocyanate" OR "Thiocyanic Acid" OR "Thiodiglycol" OR "Thiophene" OR "Thulium" OR "Titanium" OR "Titanium dioxide" OR "Toluene" OR "3,4-Toluenediamine" OR "p-Toluidine" OR "Toxaphene" OR "Triacetin" OR "1,2,4-Tribromobenzene" OR "2,4,6-Tribromophenol" OR "Tributyl phosphate" OR "Tri-Butyltin compounds" OR "Tricaine methanesulfonate" OR "Tricalcium phosphate" OR "2,4,6-Trichloroaniline" OR "1,2,3-Trichlorobenzene" OR "1,2,4-Trichlorobenzene" OR "1,1,2-Trichloroethane" OR "Trichlorofluoromethane" OR "2,4,5-Trichlorophenol" OR "2,4,6-Trichlorophenol" OR "2,4,5-Trichlorophenoxyacetic acid" OR "1,1,2-Trichloropropane" OR "1,2,3-Trichloropropene" OR "2,3,6-Trichlorotoluene" OR "alpha-2,6-Trichlorotoluene" OR "Tridymite" OR "Triethylene glycol" OR "1,1,1-Trifluoroethane" OR "Trimagnesium phosphate" OR "Trimellitic anhydride" OR "1,2,3-Trimethylbenzene" OR "1,2,4-Trimethylbenzene" OR "1,3,5-Trimethylbenzene" OR "2,2,4-Trimethylpentane" OR "2,4,4-Trimethylpentene" OR "Trimethyl phosphate" OR "1,3,5-Trinitrobenzene" OR "Trinitrophenylmethylnitramine" OR "2,4,6-Trinitrotoluene" OR "Triphenylphosphine oxide" OR "Tripoli" OR "Tripotassium phosphate" OR "Trisodium phosphate" OR "Tungsten" OR "Vanadium" OR "Vanadium pentoxide" OR "Vinyl bromide" OR "Xylene" OR	

	"Ytterbium" OR "Zirconium" OR "Acephate" OR "Acetaldehyde" OR "Acetochlor" OR "Acetone" OR "Acetonitrile" OR "Acetophenone" OR "Acetyl chloride" OR "Acifluorfen, sodium" OR "Acrolein" OR "Acrylamide" OR "Acrylic acid" OR "Acrylonitrile" OR "Adiponitrile" OR "Alachlor" OR "Alar" OR "Aldicarb" OR "Aldicarb sulfone" OR "Aldrin" OR "Ally" OR "Allyl alcohol" OR "Allyl chloride" OR "Aluminum phosphide" OR "Amdro" OR "Ametryn" OR "Aminopyridine" OR "Amitraz" OR "Ammonia" OR "Ammonium acetate" OR "Ammonium methacrylate" OR "Ammonium sulfamate" OR "Aniline" OR "Anisidine" OR "Anthracene" OR "Antimony" OR "Antimony trioxide" OR "Apollo" OR "Aramite" OR "Aroclor 1016" OR "Aroclor 1248" OR "Aroclor 1254" OR "Arsenic, Inorganic" OR "Arsine" OR "Asbestos" OR "Assure" OR "Asulam" OR "Atrazine" OR "Avermectin B1" OR "Azobenzene" OR "Barium" OR "Baygon" OR "Bayleton" OR "Baythroid" OR "Benefin" OR "Benomyl" OR "Bentazon" OR "Benz[a]anthracene" OR "Benzaldehyde" OR "Benzene" OR "Benzidine" OR "Benzo[a]pyrene" OR "Benzo[b]fluoranthene" OR "Benzo[g,h,i]perylene" OR "Benzo[k]fluoranthene" OR "Benzoic acid" OR "Benzotrichloride" OR "Benzyl chloride" OR "Beryllium" OR "Bidrin" OR "Biphenthrin" OR "Biphenyl" OR "Bis(2-chloro-1-methylethyl) ether" OR "Bis(2-chloroethoxy)methane" OR "Bis(chloroethyl)ether" OR "Bis(chloromethyl)ether" OR "Bisphenol A" OR "Boron" OR "Bromate" OR "Brominated dibenzofurans" OR "Bromobenzene" OR "Bromochloromethane" OR "Bromodichloromethane" OR "Bromodiphenyl ether" OR "Bromoform" OR "Bromomethane" OR "Bromotrichloromethane" OR "Bromoxynil" OR "Bromoxynil octanoate" OR "Butadiene" OR "n-Butanol" OR "tert-Butyl Alcohol" OR "Butyl benzyl phthalate" OR "Butylate" OR "Butylchloride" OR "Cacodylic acid" OR "Cadmium" OR "Calcium cyanide" OR "Caprolactam" OR "Captafol" OR "Captan" OR "Carbaryl" OR "Carbofuran" OR "Carbon disulfide" OR "Carbon tetrachloride" OR "Carbonyl sulfide" OR "Carbosulfan" OR "Carboxin" OR "Cerium Oxide" OR "Chloral hydrate" OR "Chloramben" OR "Chlordane" OR "Chlordecone" OR "Chlorimuron-ethyl" OR "Chlorine" OR "Chlorine dioxide" OR "Chlorite" OR "Chloro-1,1-difluoroethane" OR "Chloroacetophenone" OR "Chloroaniline" OR "Chlorobenzene" OR "Chlorobenzilate" OR "Chlorobutane" OR "Chlorocyclopentadiene" OR "Chlorodifluoromethane" OR "Chloroform" OR "Chloromethyl methyl ether" OR "Chloronaphthalene" OR "Chlorophenol" OR "Chlorophenyl methyl sulfide" OR "Chlorophenyl methyl sulfone" OR "Chloroprene" OR "Chlorothalonil" OR "Chlorotoluene" OR "Chlorpropham" OR "Chlorpyrifos" OR "Chlorsulfuron" OR "Chromium(VI)" OR "Chrysene" OR "Cobalt" OR "Coke oven emissions" OR "Copper" OR "Copper cyanide" OR "Creosote" OR "Crotonaldehyde" OR "Cumene" OR "Cyanazine" OR "Cyanide, free" OR "Cyanogen" OR "Cyanogen bromide" OR "Cyclohexane" OR "Cyclohexanone" OR "Cyclohexylamine" OR "Cyhalothrin/Karate" OR "Cypermethrin" OR "Cyromazine" OR "Dacthal" OR "Danitol" OR "Decabromodiphenyl ether" OR "Demeton" OR "Diaminotoluene" OR "Diazomethane" OR "Dibenz[a,h]anthracene" OR "Dibenzofuran" OR "Dibromo-3-	

	chloropropane" OR "Dibromobenzene" OR "Dibromochloromethane" OR "Dibromodichloromethane" OR "Dibromodiphenyl ether" OR "Dibromoethane" OR "Dibutyl phthalate" OR "Dicamba" OR "Dichloroacetic acid" OR "Dichlorobenzene" OR "Dichlorobenzidine" OR "Dichlorodifluoromethane" OR "Dichlorodiphenyl dichloroethane" OR "Dichlorodiphenyldichloroethylene" OR "Dichlorodiphenyltrichloroethane" OR "Dichloroethane" OR "Dichloroethylene" OR "Dichloromethane" OR "Dichlorophenol" OR "Dichlorophenoxy)butyric acid" OR "Dichlorophenoxyacetic acid" OR "Dichloropropane" OR "Dichloropropanol" OR "Dichloropropene" OR "Dichlorvos" OR "Dicofol" OR "Dieldrin" OR "Diesel engine exhaust" OR "Diethyl sulfate" OR "Diethyl-p-nitrophenylphosphate" OR "Diethylene glycol dinitrate" OR "Di(2-ethylhexyl)adipate" OR "Di (2-ethylhexyl)phthalate" OR "Difenzoquat" OR "Diflubenzuron" OR "Difluoroethane" OR "Diisobutyl phthalate" OR "Diisononyl phthalate" OR "Diisopropyl methylphosphonate" OR "Dimethipin" OR "Dimethoate" OR "Dimethyl phthalate" OR "Dimethyl sulfate" OR "Dimethyl terephthalate" OR "Dimethylamine" OR "Dimethylaniline" OR "Dimethylbenzidine" OR "Dimethylformamide" OR "Dimethylphenol" OR "Dinitro-o-cyclohexyl phenol" OR "Dinitrobenzene" OR "Dinitrophenol" OR "Dinitrotoluene" OR "Dinoseb" OR "Dioxane" OR "Diphenamid" OR "Diphenylamine" OR "Diphenylhydrazine" OR "Diquat" OR "Disulfoton" OR "Dithiane" OR "Diuron" OR "Dodine" OR "Endosulfan" OR "Endothall" OR "Endrin" OR "Epichlorohydrin" OR "Epoxybutane" OR "Ethephon" OR "Ethion" OR "Ethoxyethanol" OR "Ethyl acetate" OR "Ethyl carbamate" OR "Ethyl chloride" OR "Ethyl dipropylthiocarbamate" OR "Ethyl ether" OR "Ethyl Tertiary Butyl Ether" OR "Ethylbenzene" OR "Ethylene diamine" OR "Ethylene glycol" OR "Ethylene glycol monobutyl ether" OR "Ethylene oxide" OR "Ethylene thiourea" OR "Ethyleneimine" OR "Express" OR "Fenamiphos" OR "Fluometuron" OR "Fluoranthene" OR "Fluorene" OR "Fluorine" OR "Fluridone" OR "Flurprimidol" OR "Flutolanil" OR "Fluvalinate" OR "Folpet" OR "Fomesafen" OR "Fonofos" OR "Formaldehyde" OR "Formic acid" OR "Fosetyl-al" OR "Furan" OR "Furfural" OR "Furmecyclox" OR "Glufosinate-ammonium" OR "Glycidaldehyde" OR "Glyphosate" OR "Haloxyfop-methyl" OR "Harmony" OR "Heptachlor" OR "Heptachlor epoxide" OR "Heptane" OR "Hexabromobenzene" OR "Hexabromocyclododecane" OR "Hexabromodiphenyl ether" OR "Hexachlorobenzene" OR "Hexachlorobutadiene" OR "Hexachlorocyclohexane" OR "Hexachlorocyclopentadiene" OR "Hexachlorodibenzo-p-dioxin" OR "Hexachloroethane" OR "Hexachlorophene" OR "Hexahydro-1,3,5-trinitro-1,3,5-triazine" OR "Hexamethylene diisocyanate" OR "Hexane" OR "Hexanone" OR "Hexazinone" OR "Hydrogen chloride" OR "Hydrogen Cyanide" OR "Cyanide Salts" OR "Hydrogen sulfide" OR "Hydroquinone" OR "Imazalil" OR "Imazaquin" OR "Indeno[1,2,3-cd]pyrene" OR "Iprodione" OR "Isobutyl alcohol" OR "Isophorone" OR "Isopropalin" OR "Isopropyl methyl phosphonic acid" OR "Isoxaben" OR "Lactofen" OR "Lead" OR "Libby Amphibole Asbestos" OR "Limonene" OR "Linuron" OR "Londax" OR "Malathion" OR "Maleic anhydride" OR "Maleic hydrazide" OR "Maneb" OR "Manganese" OR	

	"Mepiquat chloride" OR "Mercuric chloride" OR "Mercury" OR "Merphos" OR "Metalaxyl" OR "Methacrylonitrile" OR "Methamidophos" OR "Methanol" OR "Methidathion" OR "Methomyl" OR "Methoxychlor" OR "Methoxyethanol" OR "Methyl chloride" OR "Methyl ethyl ketone" OR "Methyl iodide" OR "Methyl isobutyl ketone" OR "Methyl isocyanate" OR "Methyl methacrylate" OR "Methyl parathion" OR "Methyl tert-butyl ether" OR "Methyl-4-chlorophenoxyacetic acid" OR "Methylene Diphenyl Diisocyanate" OR "monomeric MDI" OR "polymeric MDI" OR "Methylmercury" OR "Methylnaphthalene" OR "Methylphenol" OR "Metolachlor" OR "Metribuzin" OR "Mirex" OR "Molinate" OR "Molybdenum" OR "Monochloramine" OR "Naled" OR "Naphthalene" OR "Napropamide" OR "Nickel carbonyl" OR "Nickel refinery dust" OR "Nickel subsulfide" OR "Nickel" OR "Nitrapyrin" OR "Nitrate" OR "Nitric oxide" OR "Nitrite" OR "Nitrobenzene" OR "Nitrogen dioxide" OR "Nitroguanidine" OR "Nitrophenol" OR "Nitropropane" OR "Nitrosodi-N-propylamine" OR "Nitrosodiethanolamine" OR "Nitrosodiethylamine" OR "Nitrosodimethylamine" OR "Nitrosodiphenylamine" OR "Nitrosopyrrolidine" OR "Nonabromodiphenyl ether" OR "Norflurazon" OR "NuStar" OR "Octabromodiphenyl ether" OR "Octahydro-1,3,5,7-tetranitro-1,3,5,7-tetrazocine" OR "Oryzalin" OR "Oxadiazon" OR "Oxamyl" OR "Oxyfluorfen" OR "Paclobutrazol" OR "Paraquat" OR "Parathion" OR "Pendimethalin" OR "Pentabromodiphenyl ether" OR "Pentachlorobenzene" OR "Pentachloronitrobenzene" OR "Pentachlorophenol" OR "Pentafluoroethane" OR "Perchlorate" OR "Perfluorobutanoic Acid" OR "Perfluorodecanoic Acid" OR "Perfluorohexanesulfonic Acid" OR "Perfluorohexanoic Acid" OR "Permethrin" OR "Phenanthrene" OR "Phenmedipham" OR "Phenol" OR "Phenylenediamine" OR "Phenylmercuric acetate" OR "Phosalone" OR "Phosgene" OR "Phosmet" OR "Phosphine" OR "Phosphoric acid" OR "Phthalic anhydride" OR "Picloram" OR "Pirimiphos-methyl" OR "Polychlorinated Biphenyls" OR "Polycyclic aromatic hydrocarbon" OR "Potassium cyanide" OR "Potassium silver cyanide" OR "Prochloraz" OR "Prometon" OR "Prometryn" OR "Pronamide" OR "Propachlor" OR "Propanil" OR "Propargite" OR "Propargyl alcohol" OR "Propazine" OR "Propham" OR "Propiconazole" OR "Propiolactone" OR "Propionaldehyde" OR "Propylene glycol" OR "Propylene glycol monomethyl ether" OR "Propylene oxide" OR "Propyleneimine" OR "Pursuit" OR "Pydrin" OR "Pyrene" OR "Pyridine" OR "Quinalphos" OR "Quinoline" OR "Quinone" OR "Radium 228" OR "Radium 226" OR "Radon 222" OR "Refractory ceramic fibers" OR "Resmethrin" OR "Rotenone" OR "Savey" OR "Selenious acid" OR "Selenium sulfide" OR "Selenourea" OR "Sethoxydim" OR "Silver" OR "Silver cyanide" OR "Simazine" OR "Sodium azide" OR "Sodium cyanide" OR "Sodium diethyldithiocarbamate" OR "Sodium fluoroacetate" OR "Strontium" OR "Strychnine" OR "Styrene" OR "Systhane" OR "Tebuthiuron" OR "Terbacil" OR "Terbutryn" OR "Tetrabromodiphenyl ether" OR "Tetrachlorobenzene" OR "Tetrachlorocyclopentadiene" OR "Tetrachlorodibenzo-p-dioxin" OR "Tetrachloroethane" OR "Tetrachloroethylene" OR "Tetrachlorophenol" OR "Tetrachlorovinphos"	

	OR "Tetraethyl lead" OR "Tetraethyldithiopyrophosphate" OR "Tetrafluoroethane" OR "Tetrahydrofuran" OR "Thallium" OR "Thallium acetate" OR "Thallium carbonate" OR "Thallium chloride" OR "Thallium nitrate" OR "Thallium oxide" OR "Thallium(I) sulfate" OR "Thiobencarb" OR "Thiophanate-methyl" OR "Thiram" OR "Toluene" OR "Toluene diisocyanate" OR "Toxaphene" OR "Tralomethrin" OR "Triallate" OR "Triasulfuron" OR "Tribromobenzene" OR "Tribromodiphenyl ether" OR "Tributyltin oxide" OR "Trichloro-1,2,2-trifluoroethane" OR "Trichloroacetic acid" OR "Trichlorobenzene" OR "Trichlorocyclopentadiene" OR "Trichloroethane" OR "Trichloroethylene" OR "Trichlorofluoromethane" OR "Trichlorophenol" OR "Trichlorophenoxy) propionic acid" OR "Trichlorophenoxyacetic acid" OR "Trichloropropane" OR "Tricresol" OR "Tridiphane" OR "Triethylamine" OR "Triethylene glycol monoethyl ether" OR "Trifluralin" OR "Trimethylbenzene" OR "Trimethylpentane" OR "Trinitrobenzene" OR "Trinitrotoluene" OR "Uranium" OR "Urea" OR "Vanadium pentoxide" OR "Vernam" OR "Vinclozolin" OR "Vinyl acetate" OR "Vinyl bromide" OR "Vinyl chloride" OR "Warfarin" OR "White phosphorus" OR "Xylenes" OR "Zinc" OR "Zinc cyanide" OR "Zinc phosphide" OR "Zineb" OR "environmental exposure" OR "environmental pollutant" OR Pesticide* OR "Flame Retardant" OR "Flame Retardants" OR "Fire Retardant" OR "Fire Retardants" OR "PFAS" OR "PFOS" OR "polyfluoroalkyl substances" OR "perfluoroalkyl substances" OR "polyfluoroalkyl substance" OR "perfluoroalkyl substance" OR Organophosphate* OR "Polycyclic Aromatic Hydrocarbon" OR "Polycyclic Aromatic Hydrocarbons" OR "Endocrine Disruptor" OR "Endocrine Disruptors" OR Paraben* OR Phenol* OR Bisphenol* OR Phthalate* OR "Traffic Related Air Pollution" OR "Particulate Matter")	
CRA	(cumulative OR joint OR effect modification OR mediation OR effect modifier OR mediator)	1,389,994
Reviews	(“Systematic Review*” OR “Scoping Review*” OR “Systematic Evidence Map*”)	350,702
**Combined**	Susceptibility AND Chemical AND CRA AND Reviews	1,108

Table S4. Modified AMSTAR 2.0 Tool to Evaluate Environmental Health Systematic Reviews

Guidance: Use both the publication and the protocol to identify relevant information for the AMSTAR evaluation.

<table>
<tr><td colspan="3">1. Did the research questions and inclusion criteria for the review include the components of PI/ECO?</td></tr>
<tr><td>For Yes:
☐ Population
☐ Intervention/ Exposure
☐ Comparator group
☐ Outcome</td><td>Optional (recommended)
☐ Timeframe for follow-up</td><td>Yes
No
Partial yes (if comparator does not match ours explicitly)</td></tr>
<tr><td colspan="3">2. Did the report of the review contain an explicit statement that the review methods were established prior to the conduct of the review and did the report justify any significant deviations from the protocol?</td></tr>
<tr><td>For Partial Yes:
The authors state that they had a written protocol or guide that included ALL the following:
☐ review question(s)
☐ a search strategy
☐ inclusion/exclusion criteria
☐ a risk of bias assessment</td><td>For Yes:
As for partial yes, plus the protocol should be registered/was made publicly available before the review commenced and should also have specified:
☐ a meta-analysis/synthesis plan, if appropriate, and
☐ a plan for investigating causes of heterogeneity
☐ justification for any deviations from the protocol</td><td>Yes
Partial Yes
No</td></tr>
<tr><td colspan="3">3. Did the review authors explain their selection of the study designs for inclusion in the review?</td></tr>
<tr><td colspan="2">For Yes, the review should satisfy ONE of the following:
☐ Explanation for including only RCTs
☐ OR Explanation for including only NRSI
☐ OR Explanation for the type of epidemiological exposure studies
☐ OR Explanation for including both RCTs and NRSI</td><td>Yes
No</td></tr>
<tr><td colspan="3">4. Did the review authors use a comprehensive literature search strategy?</td></tr>
<tr><td>For Partial Yes (all the following):
☐ searched at least 2 databases (relevant to research question)
☐ provided key word and/or search strategy
☐ justified publication restrictions (e.g., language)/had no publication restrictions/or clearly stated their publication restrictions for language (even without justification)</td><td>For Yes, should also have (all the following):
☐ searched the reference lists / bibliographies of included studies
☐ included/consulted content experts in the field (optional)
☐ where relevant, searched for grey literature
☐ conducted search within 24 months of submission of the review</td><td>Yes
Partial Yes
No</td></tr>
</table>

5. Did the review authors perform study selection in duplicate?

For Yes, either ONE of the following:	
☐ at least two reviewers independently agreed on selection of eligible studies and achieved consensus on which studies to include<br>☐ OR two reviewers selected a sample of eligible studies and achieved good agreement (at least 80 percent), with the remainder selected by one reviewer.<br><br>For Partial Yes:<br>One reviewer selected eligible studies (no second reviewer), however this was noted as a limitation	Yes<br><br>Partial Yes<br><br>☐ No

6. Did the review authors perform data extraction in duplicate?

For Yes, either ONE of the following:	
☐ at least two reviewers achieved consensus on which data to extract from included studies<br>☐ OR two reviewers extracted data from a sample of eligible studies and achieved good agreement (at least 80 percent), with the remainder extracted by one reviewer.<br><br>For Partial yes:<br>One reviewer selected eligible studies (no second reviewer), however this was noted as a limitation	☐ Yes<br>☐ Partial Yes<br>☐ No

7. Did the review authors provide a list of excluded studies and justify the exclusions?

For Partial Yes:	For Yes, must also have:	
☐ provided a list of all potentially relevant studies that were read in full-text form but excluded from the review<br>OR<br>if a reason (usually in the PRISMA flow and a summary of XX studies for XX reason) for the exclusions was summarized	☐ Justified the exclusion from the review of each potentially relevant study	☐ Yes<br>☐ Partial Yes<br>☐ No

8. Did the review authors describe the included studies in adequate detail?

For Partial Yes (ALL the following):	For Yes, should also have ALL the following:	
☐ described populations<br>☐ described interventions/exposures<br>☐ described comparators<br>☐ described outcomes<br>☐ described research designs	☐ described population in detail<br>☐ described intervention/exposures in detail (including doses where relevant)<br>☐ described comparator in detail (including doses where relevant)<br>☐ described study's setting<br>☐ timeframe for follow-up	☐ Yes<br>☐ Partial Yes<br>☐ No

9. Did the review authors use a satisfactory technique for assessing the risk of bias (RoB) in individual studies that were included in the review? (NOT CONSIDERED FOR SCOPING REVIEWS)

**RCTs**<br>For **Partial Yes**, must have assessed RoB from<br>☐ unconcealed allocation, *and*<br>☐ lack of blinding of patients and assessors when assessing outcomes (unnecessary for objective outcomes such as all-cause mortality)<br>AND<br>Explicitly conducting tests of internal validity using a known qualitative domain-based tool, but the authors develop an overall qualitative rating for the study (studies that cited the WHO/ILO approach (or ROBINS) which used the Nav Guide).<br><br>**NO**<br>• No mention of internal validity nor any tests conducted to assess RoB/validity.<br>• If the study uses a scoring system (quantitative) to numerically score studies	For **Yes**, must also have assessed RoB from:<br>☐ allocation sequence that was not truly random, *and*<br>☐ selection of the reported result from among multiple measurements or analyses of a specified outcome<br>AND you need to have both of the below.<br>o Explicitly conducting tests of internal validity using a known qualitative domain-based tool (a citable protocol). They may use different ones.<br>o Presents results from the entire internal validity tool (e.g., responses to each question and not only the overall ROB rating for each study)	☐ Yes<br>☐ Partial Yes<br>☐ No<br>☐ Includes only NRSI

**NRSI**<br>For **Partial Yes**, must have assessed RoB:<br><br>**Partial**<br><br>Explicitly conducting tests of internal validity using a known qualitative domain-based tool, but the authors develop an overall qualitative rating for the study (studies that cited the WHO/ILO approach (or ROBINS) which used the Nav Guide).<br><br>**NO**<br><br>o No mention of internal validity nor any tests conducted to assess RoB/validity.<br><br>o If the study uses a scoring system (quantitative) to numerically score studies	For **Yes**, you need to have both of the below:<br><br>• Explicitly conducting tests of internal validity using a known qualitative domain-based tool (a citable protocol). They may use different ones.<br>• Presents results from the entire internal validity tool (e.g., responses to each question and not only the overall ROB rating for each study).	☐ Yes<br>☐ Partial Yes<br>☐ No<br>☐ Includes only RCTs

10. Did the review authors report on the sources of funding and COI for the studies included in the review?

For Yes

☐ Must have reported on the sources of funding & COI for individual studies included in the review. Note: Reporting that the reviewers looked for this information but it was not reported by study authors also qualifies

Options: Yes/No

11. If meta-analysis was performed did the review authors use appropriate methods for statistical combination of results?

For Yes:	
☐ The authors justified combining the data in a meta-analysis<br>☐ AND they statistically combined effect estimates from NRSI that were adjusted for confounding, rather than combining raw data, or justified combining raw data when adjusted effect estimates were not available	☐ Yes<br>☐ No<br>☐ No meta-analysis conducted

12. If meta-analysis was performed, did the review authors assess the potential impact of RoB in individual studies on the results of the meta-analysis or other evidence synthesis? (NOT CONSIDERED FOR SCOPING REVIEWS)

For Yes:	
☐ If the pooled estimate was based on RCTs/exposure studies and/or NRSI at variable RoB, the authors performed analyses to investigate possible impact of RoB on summary estimates of effect.	☐ Yes<br>☐ No<br>☐ No meta-analysis conducted

13. Did the review authors account for RoB in individual studies when interpreting/ discussing the results of the review? (NOT CONSIDERED FOR SCOPING REVIEWS)

For Yes:	
☐ If RCTs/Exposure studies with moderate or high RoB, or NRSI were included the review provided a discussion of the likely impact of RoB on the results	☐ Yes<br>☐ No

14. If meta-analysis was performed, did the review authors provide a satisfactory explanation for, and discussion of, any heterogeneity observed in the results of the review? (NOT CONSIDERED FOR SCOPING REVIEWS)

For Yes:	
☐ There was no significant heterogeneity in the results<br>☐ OR if heterogeneity was present the authors performed an investigation of sources of any heterogeneity in the results and discussed the impact of this on the results of the review<br><br>If meta-analysis was not performed, did the review authors discuss the reasons why, including a discussion of heterogeneity?	☐ Yes<br>☐ No

15. Did the review authors assess publication bias (small study bias) and discuss its likely impact on the results of the review?

For Yes:	
☐ performed graphical or statistical tests for publication bias and discussed the likelihood and magnitude of impact of publication bias	☐ Yes<br>☐ No<br>☐ No meta-analysis conducted

16. **Did the review authors report any potential sources of conflict of interest, including any funding they received for conducting the review?**	
For Yes:<br>□ For "Yes", "1) There is an author COI statement and it is concordant with the authors affiliations and 2) there is a study funding statement" - If any of this information is missing, or it isn't concordant, then it is a "NO"	□ Yes<br>□ No

To cite this tool: Shea BJ, Reeves BC, Wells G, Thuku M, Hamel C, Moran J, Moher D, Tugwell P, Welch V, Kristjansson E, Henry DA. AMSTAR 2: a critical appraisal tool for systematic reviews that include randomised or non-randomised studies of healthcare interventions, or both. BMJ. 2017 Sep 21;358:j4008.

Table S5. Justifications for Exclusion at Full Text (n=79)

Author/Year	Title	Exclusion Justification
Heo et al., 2021	Suicide and Associations with Air Pollution and Ambient Temperature: A Systematic Review and Meta-Analysis	No interaction between extrinsic susceptibility factor and environmental agent
Vasconcellos et al., 2014	Societal-level Risk Factors Associated with Pediatric Hearing Loss: A Systematic Review	No interaction between extrinsic susceptibility factor and environmental agent
Habib et al., 2014	Gender in occupational health research of farmworkers: a systematic review	No interaction between extrinsic susceptibility factor and environmental agent
Chen et al., 2017	Does temperature modify the effect of PM(10) on mortality? A systematic review and meta-analysis	No interaction between extrinsic susceptibility factor and environmental agent
García-Gómez et al., 2022	In the pursuit of new social neurons. Neurogenesis and social behavior in mice: A systematic review	No interaction between extrinsic susceptibility factor and environmental agent
Fernandes et al., 2023	School-Based Interventions to Support Healthy Indoor and Outdoor Environments for Children: A Systematic Review	No interaction between extrinsic susceptibility factor and environmental agent
González-Alzaga et al., 2014	A systematic review of neurodevelopmental effects of prenatal and postnatal organophosphate pesticide exposure	No interaction between extrinsic susceptibility factor and environmental agent
Sheehan et al., 2014	Global methylmercury exposure from seafood consumption and risk of developmental neurotoxicity: a systematic review	No interaction between extrinsic susceptibility factor and environmental agent
Bahns et al., 2021	Occupational risk factors for meniscal lesions: a systematic review and meta-analysis	No interaction between extrinsic susceptibility factor and environmental agent
Adaji et al., 2019	Understanding the effect of indoor air pollution on pneumonia in children under 5 in low- and middle-income countries: a systematic review of evidence	No interaction between extrinsic susceptibility factor and environmental agent
Balachandar et al., 2023	Association Between Blood Lead Levels and Thyroid Function: An Updated Systematic Review and Meta-Analysis	No interaction between extrinsic susceptibility factor and environmental agent
Kazemi et al., 2022	Comparison of dietary and physical activity behaviors in women with and without polycystic ovary syndrome: a systematic review and meta-analysis of 39471 women	No interaction between extrinsic susceptibility factor and environmental agent
Farace et al., 2020	Amyotrophic lateral sclerosis and lead: A systematic update	No interaction between extrinsic susceptibility factor and environmental agent
Gaio et al., 2019	Ambient air pollution and lipid profile: Systematic review and meta-analysis	No interaction between extrinsic susceptibility factor and environmental agent
Ellis et al., 2023	Adult Organophosphate and Carbamate Insecticide Exposure and Sperm Concentration: A Systematic Review and Meta-Analysis of the Epidemiological Evidence	No interaction between extrinsic susceptibility factor and environmental agent

Gutiérrez-Torres et al., 2018	Prenatal Exposure to Endocrine Disruptors and Cardiometabolic Risk in Preschoolers: A Systematic Review Based on Cohort Studies	No interaction between extrinsic susceptibility factor and environmental agent
Hernández-Ruiz et al., 2019	A Review of A Priori Defined Oxidative Balance Scores Relative to Their Components and Impact on Health Outcomes	No interaction between extrinsic susceptibility factor and environmental agent
Gao et al., 2020	A Systematic Review and Meta-Analysis of Short-Term Ambient Ozone Exposure and COPD Hospitalizations	No interaction between extrinsic susceptibility factor and environmental agent
Bilal et al., 2023	Surface water quality, public health, and ecological risks in Bangladesh-a systematic review and meta-analysis over the last two decades	No interaction between extrinsic susceptibility factor and environmental agent
Clark et al., 2020	The association between traffic-related air pollution and obstructive sleep apnea: A systematic review	No interaction between extrinsic susceptibility factor and environmental agent
Dalrymple et al., 2018	Can antenatal diet and lifestyle interventions influence childhood obesity? A systematic review	No interaction between extrinsic susceptibility factor and environmental agent
Gallego-Viñas et al., 2019	Chronic mercury exposure and blood pressure in children and adolescents: a systematic review	No interaction between extrinsic susceptibility factor and environmental agent
Yazdani Abyaneh et al., 2017	Giant Basal Cell Carcinomas Express Neuroactive Mediators and Show a High Growth Rate: A Case-Control Study and Meta-Analysis of Etiopathogenic and Prognostic Factors	No interaction between extrinsic susceptibility factor and environmental agent
Kalyvas et al., 2016	Maternal environmental risk factors for congenital hydrocephalus: a systematic review	No interaction between extrinsic susceptibility factor and environmental agent
Agrawal et al., 2021	Early life exposures and the risk of inflammatory bowel disease: Systematic review and meta-analyses	No interaction between extrinsic susceptibility factor and environmental agent
Ballesteros et al., 2017	Exposure to perfluoroalkyl substances and thyroid function in pregnant women and children: A systematic review of epidemiologic studies	No interaction between extrinsic susceptibility factor and environmental agent
Braithwaite et al., 2019	Air Pollution (Particulate Matter) Exposure and Associations with Depression, Anxiety, Bipolar, Psychosis and Suicide Risk: A Systematic Review and Meta-Analysis	No interaction between extrinsic susceptibility factor and environmental agent
Brasil et al., 2018	Pesticides as risk factors for head and neck cancer: A review	No interaction between extrinsic susceptibility factor and environmental agent
Duan et al., 2023	Risk factors of amyotrophic lateral sclerosis: a global meta-summary	No interaction between extrinsic susceptibility factor and environmental agent
Malacarne et al., 2022	The built environment as determinant of childhood obesity: A systematic literature review	No interaction between extrinsic susceptibility factor and environmental agent
Rivera-Núñez et al., 2022	Phenols, Parabens, Phthalates and Puberty: a Systematic Review of Synthetic Chemicals	No interaction between extrinsic susceptibility factor and environmental agent

	Commonly Found in Personal Care Products and Girls' Pubertal Development	
Xu et al., 2015	Meta-analysis of modifiable risk factors for Alzheimer's disease	No interaction between extrinsic susceptibility factor and environmental agent
Yang et al., 2018	Global association between ambient air pollution and blood pressure: A systematic review and meta-analysis	No interaction between extrinsic susceptibility factor and environmental agent
Lin et al., 2020	Residential exposure to petrochemical industrial complexes and the risk of leukemia: A systematic review and exposure-response meta-analysis	No interaction between extrinsic susceptibility factor and environmental agent
Meltzer et al., 2020	A Systematic Review of Environmental Health Outcomes in Selected American Indian and Alaska Native Populations	No interaction between extrinsic susceptibility factor and environmental agent
Okasha et al., 2003	Exposures in childhood, adolescence and early adulthood and breast cancer risk: a systematic review of the literature	No interaction between extrinsic susceptibility factor and environmental agent
Dos Santos Oliveira et al., 2023	The interplay between DNA methylation and cardiac autonomic system functioning: a systematic review	No interaction between extrinsic susceptibility factor and environmental agent
Fujii et al., 2022	DNA methylation as a mediator of associations between the environment and chronic diseases: A scoping review on application of mediation analysis	No interaction between extrinsic susceptibility factor and environmental agent
Ports et al., 2019	Adverse Childhood Experiences and the Presence of Cancer Risk Factors in Adulthood: A Scoping Review of the Literature From 2005 to 2015	No interaction between extrinsic susceptibility factor and environmental agent
Brooks and Brooks, 2021	A systematic review: what factors predict Post-Traumatic Stress Symptoms in ambulance personnel?	No interaction between extrinsic susceptibility factor and environmental agent
Benmarhnia et al., 2018	Heterogeneity in the Relationship between Disinfection By-Products in Drinking Water and Cancer: A Systematic Review	No interaction between extrinsic susceptibility factor and environmental agent
Carracedo-Martínez et al., 2010	Case-crossover analysis of air pollution health effects: a systematic review of methodology and application	No interaction between extrinsic susceptibility factor and environmental agent
Ciabattini et al., 2021	Systematic review and meta-analysis of recent high-quality studies on exposure to particulate matter and risk of lung cancer	No interaction between extrinsic susceptibility factor and environmental agent
Clarke et al., 2022	Moving from nature to nurture: a systematic review and meta-analysis of environmental factors associated with juvenile idiopathic arthritis	No interaction between extrinsic susceptibility factor and environmental agent
Hossain et al., 2019	Sociodemographic, climatic variability and lower respiratory tract infections: a systematic literature review	No interaction between extrinsic susceptibility factor and environmental agent
Williams and Ross, 2007	Consequences of prenatal toxin exposure for mental health in children and adolescents: a systematic review	No interaction between extrinsic susceptibility factor and environmental agent
Putra et al., 2020	The Relationship Between Green Space and Prosocial Behaviour Among Children and Adolescents: A Systematic Review	No interaction between extrinsic susceptibility factor and environmental agent

Boogaard et al., 2023	Long-term exposure to traffic-related air pollution and non-accidental mortality: A systematic review and meta-analysis	No interaction between extrinsic susceptibility factor and environmental agent
Georgakis et al., 2017	Season of birth and primary central nervous system tumors: a systematic review of the literature with critical appraisal of underlying mechanisms	No interaction between extrinsic susceptibility factor and environmental agent
da Silva Junior et al., 2023	Empirical relationship between chromosomal damage and airborne particulate matter: A systematic review and meta-analysis of studies in exposed populations	No interaction between extrinsic susceptibility factor and environmental agent
García-Hernández et al., 2020	Exposure to ultrafine particles in children until 18 years of age: A systematic review	No interaction between extrinsic susceptibility factor and environmental agent
Ribeiro et al., 2018	Neighborhood Socioeconomic Deprivation and Allostatic Load: A Scoping Review	No interaction between extrinsic susceptibility factor and environmental agent
Shahunja et al., 2022	Family, neighborhood and psychosocial environmental factors and their associations with asthma in Australia: a systematic review and Meta-analysis	No interaction between extrinsic susceptibility factor and environmental agent
Sundaresan et al., 2015	Occupational and environmental risk factors for chronic rhinosinusitis: a systematic review	No interaction between extrinsic susceptibility factor and environmental agent
Chandia-Poblete et al., 2022	The influence of air pollution exposure on the short- and long-term health benefits associated with active mobility: A systematic review	No interaction between extrinsic susceptibility factor and environmental agent
Ledda et al., 2023	Unmasking the Hidden Danger: A Decade-Long Systematic Review of Case-Control Studies on Single Occupational Risks and Prostate Cancer	No interaction between extrinsic susceptibility factor and environmental agent
Albers et al., 2014	Modifiable risk factors for primary headache. A systematic review	No interaction between extrinsic susceptibility factor and environmental agent
Zona et al., 2023	SENTIERI - Epidemiological Study of Residents in National Priority Contaminated Sites. Sixth Report	No interaction between extrinsic susceptibility factor and environmental agent
Moore et al., 2024	Exposure to air pollutants and rheumatoid arthritis biomarkers: A scoping review	No interaction between extrinsic susceptibility factor and environmental agent
Shah and Knowledge Synth Grp Determinants, 2010	Paternal factors and low birthweight, preterm, and small for gestational age births: a systematic review	No interaction between extrinsic susceptibility factor and environmental agent
Migkos et al., 2019	Epidemiological characteristics of psoriatic arthritis	No interaction between extrinsic susceptibility factor and environmental agent
Navas-Acien et al., 2007	Lead exposure and cardiovascular disease - A systematic review	No interaction between extrinsic susceptibility factor and environmental agent

Stanhope et al., 2021	The relationship between the neighborhood built and social environment and hypertensive disorders of pregnancy: A scoping review	No interaction between extrinsic susceptibility factor and environmental agent
Tsuji et al., 2015	Low-level arsenic exposure and developmental neurotoxicity in children: A systematic review and risk assessment	No interaction between extrinsic susceptibility factor and environmental agent
Aboud and Yousafzai, 2015	Global Health and Development in Early Childhood	No interaction between extrinsic susceptibility factor and environmental agent
Boogaard et al., 2023	Evidence Synthesis of Observational Studies in Environmental Health: Lessons Learned from a Systematic Review on Traffic-Related Air Pollution	No interaction between extrinsic susceptibility factor and environmental agent
Dzhambov et al., 2020	Analytical approaches to testing pathways linking greenspace to health: A scoping review of the empirical literature	No interaction between extrinsic susceptibility factor and environmental agent
Fox et al., 2021	Cumulative Risks from Stressor Exposures and Personal Risk Factors in the Workplace: Examples from a Scoping Review	No interaction between extrinsic susceptibility factor and environmental agent
Casey et al., 2023	Methods in Public Health Environmental Justice Research: a Scoping Review from 2018 to 2021	No interaction between extrinsic susceptibility factor and environmental agent
Ghosh et al., 2021	Ambient and household PM2.5 pollution and adverse perinatal outcomes: A meta-regression and analysis of attributable global burden for 204 countries and territories	No interaction between extrinsic susceptibility factor and environmental agent
Goodman et al., 2002	Neurobehavioural testing in workers occupationally	No interaction between extrinsic susceptibility factor and environmental agent
Gu et al., 2023	Appraising causal risk and protective factors for rheumatoid arthritis	No interaction between extrinsic susceptibility factor and environmental agent
Boffetta et al., 2012	Occupational exposure to beryllium and cancer risk: a review of the epidemiologic evidence	Fails PRISMA 6
Kondo et al., 2019	Meta-Analysis of Heterogeneity in the Effects of Wildfire Smoke Exposure on Respiratory Health in North America	Fails PRISMA 11
Hu et al., 2007	The epidemiology of lead toxicity in adults: measuring dose and consideration of other methodologic issues	Not a review
Rosa et al., 2018	Evidence establishing a link between prenatal and early-life stress and asthma development	Fails PRISMA 5
Nardone et al., 2018	Ambient Air Pollution and Asthma-Related Outcomes in Children of Color of the USA: a Scoping Review of Literature Published Between 2013 and 2017	Fails PRISMA 5
Mocelin et al., 2022	Adverse early-life environmental exposures and their repercussions on adult respiratory health	Fails PRISMA 5
Gardner-Frolick et al., 2022	Selecting Data Analytic and Modeling Methods to Support Air Pollution and Environmental Justice	No relevant population

	Investigations: A Critical Review and Guidance Framework	

Table S6. Justifications for Exclusion at Evaluation (n=4)

Author/Year	Title	Exclusion Justification
Deguen et al., 2022	Exposome and Social Vulnerability: An Overview of the Literature Review	Review of reviews
Requia et al., 2018	Global Association of Air Pollution and Cardiorespiratory Diseases: A Systematic Review, Meta-Analysis, and Investigation of Modifier Variables	The authors did not use the primary studies identified in the SR to evaluate the relationship with modifiers.
Baldacci et al., 2018	Environmental and individual exposure and the risk of congenital anomalies: a review of recent epidemiological evidence	Study included both primary studies and "other studies;" chemical exposures and SES are each explored independently of each other.
Zhao et al., 2021	Environmental factors and risks of cognitive impairment and dementia: A systematic review and meta-analysis	The majority of social stressors were treated as exposures and did not evaluate the interaction or moderation of social stressors on the environmental exposures and outcome relationship.

Table S7. Results of Overlap Assessment for Two Reviews Evaluating the Impact of Air Pollution on Child Neurodevelopment (GROOVE Tool)

Air Pollution/Child Neurodevelopment

Primary Studies	*Systematic Review* Yu et al. 2022	*Scoping Review* Payne-Sturges et al. 2023
Becerra et al. 2013	x	
Gong et al. 2017	x	
Dickerson et al. 2016	x	x
McGuinn et al. 2019	x	x
Kalkbrenner et al. 2010	x	
Al-Hamdan et al. 2018	x	x
Wang et al. 2017		x
Padula et al. 2017		x
Pagliaccio et al. 2020		x
Lovasi et al. 2014		x
Cowell et al. 2015		x
Perera et al. 2009		x
Suarez et al. 2007		x

Table S8. Population, exposures, outcomes, and number of primary studies identified by moderate and low-quality scoping reviews included in the analysis (n=2)

Scoping Review	Population	Chemical Exposure	Non-Chemical Stressor	Outcome	# primary studies (date range)
Payne-Sturges et al. 2023	US Children	Ambient air pollution	SES	Neurodevelopment	5 (2014-2020)
	US Children	Ambient air pollution	Race/ethnicity	Neurodevelopment	4 (2007-2018)
	US Children	Lead	SES	Neurodevelopment	15 (1987-2020)
	US Children	Lead	Race/ethnicity	Neurodevelopment	11 (1993-2022)
	US Children	Mercury	SES	Neurodevelopment	2 (2006-2014)
	US Children	OPs	SES	Neurodevelopment	3 (2014-2019)
	US Children	OPs	Race/ethnicity	Neurodevelopment	6 (2006-2022)
	US Children	PCBs	Sociodemographic variables	Neurodevelopment	1 (2014)
	US Children	Phthalates	Race	Neurodevelopment	1 (2021)
	US Children	Multiple chemical exposures/ mixtures	SES	Autism spectrum disorder	1 (2016)
	US Children	Multiple chemical exposures/ mixtures	Race	Neurodevelopment	2 (2017-2020)
Deziel et al. 2020	Children	Proximity to oil and gas activity	Urban/Rural	Pulmonary artery and valve defects; aortic artery and valve defects; Construal defects; Tricuspid valve defects	1 (2019)
	Infants	Proximity to oil and gas activity	Private water and/or environmental law violations	Infant mortality	1 (2017)
	Women of reproductive age	Proximity to oil and gas activity	Family income	Antenatal anxiety and depression	1 (2019)

Table S9. Detailed Review Results

Exposure	Outcome	Non-Chemical Stressor	Non-Chemical Stressor Measurement/Definition	Population	Results Reported in Review	Certainty in the Body of Evidence	Certainty in the Body of Evidence Rationale	Author's Conclusions	Contributing Primary Studies	Overall Review Conclusion
					**Rodriquez-Villamizar et al. 2016**					
Air pollution	Asthma-related hospitalization	Socioeconomic position		Children	Neidell (14) found that the effects of NO2 and CO in California were larger than other CAPs; however, O3 had a larger effect on children living in low SEP areas, especially for those aged 3–6 and 12–18 years, for which the interaction terms were statistically significant [β = 0.091 (p < 0.01) and β = 0.042 (p < 0.05), respectively]. Yap et al. (25) analyzed the effect of PM2.5 on hospitalizations for asthma in children 1–9 years old in 12 California counties. The analysis of SEP modification-role yielded non-significant interactions; however, the associations between PM2.5 exposure and asthma hospitalizations were stronger in areas with higher SEP in the Central Valley region (RR 0.98 95%CI 0.95–1.01 for low SEP and RR 1.07 95%CI 1.04–1.09 for high SEP).<br><br>When using lags between 0 and 5 days for NO2, O3 and PM10, the associations with asthma-related hospitalizations were higher for children living in low SEP areas; however, the 95% CI for high versus low SEP do overlap. For CO and SO2, there was no clear pattern of effect-modification by SEP in the included studies. From those three studies, only Lee et al. (23) assessed statistical interaction found non-significant results.	n/a	n/a	The results revealed that associations between hospitalizations and air pollutants seem to have a stronger negative influence on children living in low SEP conditions. However, confirmation of the effect modification by statistically significant interactions between air pollutants and SEP was evident only in one out of five studies, probably related to sample size limitations of the original studies.	Neidell 2004<br>Yap et al. 2013<br>Lin et al. 2004<br>Lee et al. 2006<br>Bae et al. 2010	
Air pollution	Asthma-related ED visits/calls	Socioeconomic position	We used Krieger's definition of SEP for this review and considered any single or combined measure related to income, education or occupation, as a SEP measure. Aggregated SEP measures were defined as area based SEP measures generally based on census data.	Children	Three studies assessed the role of SEP as an effect modifier on the association between air pollutants and asthma-related ED visits/calls. Gleason et al. (16) assessed the effect of same-day PM2.5 using the proportion of people living in poverty according to the New Jersey census as the SEP indicator. The study did not find any pattern of effect modification by SEP level stratification and the interaction terms were not statistically significant. The effect of same day O3 on asthma ED visits also was not modified by SEP aggregated measures in another study (Figure 3) (15, 16). In Strasbourg, France, the effects of SO2, PM10 and NO2 on emergency calls were not different by SEP level; the interaction terms for SEP and the categorical and continuous variable were not significant (15). In contrast, Shmool et al. (26) used an exploratory ecologic spatial analysis in New York City and found a statistically significant modification effect of NO2 on asthma ED visits by SEP (i.e. defined as a factor two mainly characterized by "crowding and poor access to resources"; p-value of interaction term < 0.05). The negative effect was higher in city areas in which the SEP indicator was above the median (β = 0.055±SE = 0.026 for low SEP and β = –0.001±SE = 0.008 for high SEP). However, the authors reported non-significant interaction terms (absence of effect modification) in the association between NO2 and asthma ED visits when using area-level poverty rates as a SEP modifier.	n/a	n/a	Overall, we found three studies failing to demonstrate any SEP differential effect on the association between pollutant exposure and asthma-related ED visits/calls.	Laurent et al. 2008<br>Gleason et al. 2014<br>Schmool et al. 2014	***This review reveals that there is weak evidence of SEP as an effect-modifier of the association between air pollution and children's asthma exacerbations. While stronger negative effects on asthma-related hospitalizations occur for children living in a lower SEP, the sample size of some of the original studies limited the statistical assessment of the modification effect.***
Air pollution	Ambulatory visits	Socioeconomic position		Children	Burra et al. (13) measured social disadvantage and assessed the effect modification on the association between air pollution and ambulatory visits for asthma in an urban setting. For children under 18 years old, there was a significant SEP quintile ratio (Q1/Q5 = 1.006 95%CI 1.000–1.013) using the single day SO2 exposure. There was also a significant difference in the SEP Q1/ Q5 risk ratio using the PM2.5 5-day cumulative average exposure for males and single-day exposure for females (RR = 1.017 95%CI 1.002–1.031 and RR = 1.012 95%CI 1.001–1.023, respectively). No significant associations were found between O3 and ambulatory asthma visits.	n/a	n/a	None	Burra et al. 2009	
Air pollution	Repeat hospital visits	Socioeconomic position		Children	Delfino et al. (27) studied the effect of traffic-related air pollution on repeated hospital encounters for asthma in urban California. Using different SEP measures at census block level, they found a stronger association with NOx among children living in higher income areas (HR = 1.07, 95%CI 0.98–1.16 for low SEP vs. HR = 1.14, 95%CI 0.006–1.24 for high SEP measured by median household income); however, the assessment of statistical interaction gave non-significant results and the authors suggested that this result might have been attributed to more accurate data collected in this group.	n/a	n/a	None	Delfino et al. 2009	
					**Vesterinen et al., 2017**					
Smoking	Birth weight	Stress	Stress exposures are difficult to measure and were measured variably across the human studies; stress exposure can be broadly grouped into place-based stress (clusters of high stress conditions such as neighborhoods of greater poverty) and individual-based (e.g. discrimination and number of life events). Some of these exposures are direct and others are indirect; however in the present study we considered all exposures equally, including exposures which can be classified as indicators of stress response (e.g. maternal mood) and stressors (e.g. SES). Additionally, we found little methodological consistency across studies in their measurement of chronic psychosocial stress.	Humans (during the reproductive or developmental time period)	Smoking in the presence of high stress was associated with a significant reduction in mean birth weight for all five different stress comparisons (Fig 4A), a significantly greater odds of LBW for all five comparisons (Fig 4C).<br><br>We found a statistically significant interaction between smoking and high financial difficulty on mean birth weight (Fig 4B) and lower social class on odds of LBW (Fig 4D). For data on relative reduction in birth weight, smoking among those with lower educational attainment resulted in a significant interaction on the original scale. The remaining interaction terms on smoking and stress were either elevated but not significant, were null, or were in the opposite direction (n = 4 for mean birth weight (Fig 4B) and n = 3 for odds of LBW; Fig 4D).	n/a	n/a	We found evidence for a combined effect of chemical exposure and stress on birth weight.	Phung 2003<br>Newton 1984<br>Rogers 1998<br>Dubois 2006<br>Comstock 1971<br>da Silva 1992<br>Lumley 1985<br>Miller 1987<br>Gyllstrom 2011	
Air pollution	Birth weight	Stress		Humans (during the reproductive or developmental time period)	Of the other 11 chemicals and hazards (which were mostly air pollution-related exposures), we in general found that with the exception of traffic proximity exposures (distance to major highways, cumulative traffic density and residential proximity to highway), there was a significant decrease in BW or increasing the odds of LBW in the presence of high-stress compared to the control group (low stress/low chemical) (Fig 5A and 5B).<br><br>Their interaction was less clear; a similar number of air pollutants in the presence and absence of stress (either race or neighborhood poverty) had a significant and non-significant interaction on the original scale; we found an interaction between benzene with high occupational stress and reduced birth weight [81] (Fig 4B). We found that the interactions between alcohol and low maternal mood [36] or low SES [32] were essentially null or negative, but with confidence intervals that crossed one (Fig 4D). Interaction results for traffic proximity variables combined with stress were essentially null (Fig 5B).	n/a	n/a		Zeka 2008<br>Morello-Frosch 2010<br>Bell 2007<br>Chen 2000<br>Gyllstrom 2011<br>Lumley 1985	***Overall we found this to be an understudied topic and recommend further investigation in high quality, pragmatically designed animal or human observational studies. However, within the context of the small body of evidence currently available, we found that exposure to several chemicals in the absence of stress is associated with a reduction in fetal growth in humans, and this effect is generally larger in***

Smoking	Low birth weight	SES	SES or proxies for SES (social class, years of paternal education)	Humans (during the reproductive or developmental time period)	Smoking was associated with significantly increased odds of LBW in the high stress (low SES) group (combined exposure, OR 4.75 (2.46±9.16); X2 = 33.28, I2 = 91.0%) compared to the low stress group (high SES) (OR 1.95 (95% CI 1.53±2.48); X2 = 1.49, I2 = 0%). There was also an elevated but not statistically significant odds ratio of LBW for high stress (low SES) compared to low stress among non-smokers (OR 1.34 (0.82±2.20); X2 = 16.24, I2 = 81.5%). The pooled interaction term from the meta-analysis was positive but not significant (1.87 (0.78±4.48); X2 = 14.25, I2 = 79.0%; Fig 6). Due to the small number of included studies we did not formally assess for publication bias.	n/a	n/a	Our meta-analysis results on the effect of smoking and low SES, found a clear trend towards a reduction in birth weight for smoking in both the absence and presence of low SES.	Lumley 1985<br>da Silva 1992<br>Comstock 1971<br>Miller 1987	***the presence of stress. Conversely, we found little effect of stress in the absence of chemical exposure. Our assessment of the animal data were limited by the small number of studies on each chemical and stressor and by limitations to their quality, particularly the lack of allocation concealment, sequence generation and blinding. Overall, we found that prenatal exposure for several chemicals is harmful, but there is limited evidence of interaction with stress exposure, and combined exposure was associated with significantly reduced fetal weight in comparison to either exposure alone in about half of studies.***
Various chemicals<br><br>alcohol (n = 5 studies), aluminum (n = 3), caffeine (n = 1), diesel exhaust particulates (n = 1), lead (n = 2), methylmercury chloride (n = 1), PFOS (n = 3), sodium arsenate (n = 1), toluene (n = 3) and uranium (n = 2)	Pre- or post-natal offspring weight	Stress	restraint (n = 18) heat (n = 1), nest material restriction (n = 1), no light (n = 1), restraint with bright light (n = 1), and various stressors (n = 2)	Animals (mammalians)	*Combined effect of chemical and stress exposures.* Of the ten chemicals we evaluated quantitatively, there was a significant reduction in fetal growth outcomes when we evaluated prenatal aluminum exposure and prenatal restraint stress in mice combined at all times of assessment and rats at all but the earliest times points and one comparison measured at postnatal day 21 (Fig 7). Additionally we found a trend towards a reduction in weight when toluene (assessed in two studies with ten comparisons) was paired with restraint or a variety of stressors (data not shown). For all other chemical and stress exposures there was no clear trend.<br><br>*Interaction* . In 98 comparisons from four studies, we found five comparisons where the combined exposure to stress and chemicals was associated with significantly lower weight than either exposure alone; these were for toluene and various stressors, aluminum and restraint (3 comparisons, 2 studies) and sodium arsenite and restraint. For chemicals including alcohol, PFOS, lead and uranium we found no evidence of a negative combined exposure. We found a trend towards positive interactions for toluene paired with restraint or various stressors in rats; however, all but one comparison had wide confidence intervals which crossed the line of no effect. Interestingly we found a trend towards negative interactions (albeit most comparisons were not significant) for both alcohol paired with restraint, heat or light in mice and uranium paired with restraint in rats. For all other chemicals and stressors, there was no consistent trend.	n/a	n/a	In general the findings from animal studies were not consistent; however, the dataset was extremely heterogeneous and sparse.<br><br>When animals were exposed to both the chemical and stress, we found a trend towards a relationship between aluminum, restraint stress and reduction in weight for mice at all times points; for rats we observed effects at all but the earliest time points, for toluene and restraint or various stressors, and uranium and restraint. Interestingly, we did not find clear interactions for any of the pairs of exposures although there was a trend towards positive interactions for alcohol in mice. We also found a trend towards negative interactions	Colomina 1997<br>Ward 1989<br>Ward 1989<br>Ward 1994<br>Ward 1996<br>Colomina 1998<br>Colomina 2005<br>Albina 2002<br>Colomina 1999<br>Bolton 2013<br>Cory-Slechta 2004<br>Cory-Slectha 2013<br>Colomina 1995<br>Fuentes 2006<br>Fuentes 2007<br>Ribes 2010<br>Rasco 1994<br>Hougaard 2003<br>Hougaard 2005<br>Ladefoged 2004<br>Albina 2003<br>Sanchez 2006	
**Rugel and Brauer, 2020**										
Traffic- related air pollution	Cardiovascular disease	Noise			There was sufficient evidence for small increases in poor cardiovascular outcomes associated with higher noise exposures based on four of 13 studies that reported results for noise as a primary exposure and adjusted for TRAP (Roswall et al. 2017a; Sørensen et al., 2012, 2014b, 2017), all four of which had minimal bias. However, it is important to note that although the studies assessed four distinct outcomes under the broad category of cardiovascular disease, their samples were drawn from a single underlying cohort: the Danish Diet, Cancer, and Health cohort. In addition, there was sufficient evidence for a lack of effect of TRAP on CVD morbidity after adjustment for noise, reported in 11 of 13 studies (Bodin et al., 2016; Cai et al., 2018; Carey et al., 2016; de Kluizenaar et al., 2018; Hoffmann et al., 2015; Linares et al., 2006; Nieuwenhuijsen et al., 2019; Roswall et al., 2017a; Sørensen et al., 2011; Tonne et al., 2016; Vivanco-Hidalgo et al., 2019) which included participants of a wide range of ages, from 15 to 83. Other factors leading to the sufficient rating for this set of exposures include a high degree of precision, consistency among exposure metrics and effect sizes (or lack thereof), and no indications of publication bias.			See Column F.	See Column F.	
Traffic- related air pollution	Mortality	Noise	In the context of this review, traffic-related air pollution (TRAP) was restricted to air pollutants associated with the most common forms of urban transportation, including cars, trucks, buses, and non-electric trains and streetcars, but not including air or marine traffic as a potential source. Noise exposure was restricted to non-occupational contexts, to include the noise generated by industrial environments but not exposures to sounds that occur inside settings such as factories. Natural spaces comprise a wide range of natural environments along a continuum from completely wild to fully human-designed, comprising surrounding greenness based on surface-reflectance values derived from satellite-based platforms; greenspace, defined as any area containing or comprising vegetation and including parks, gardens, playgrounds, and street trees;		Looking at the relationship with TRAP as the primary exposure, there was sufficient evidence for a slight increase in mortality risk associated with higher TRAP exposures: of the six studies that included sufficiently detailed results, four reported increased risk (Beelen et al., 2008; Gan et al., 2012a; Hvidtfeldt et al., 2019; Roswall et al., 2017a). Sørensen et al., 2014b only reported an increased risk for TRAP at the highest levels and in association with high noise levels, so it was not counted as contributing toward the evidence. Looking at the four studies that examined noise as a primary exposure and adjusted for TRAP, there was only limited evidence for small increases in mortality, with three studies reporting such an association (Gan et al., 2012a; Roswall et al., 2017a; Sørensen et al., 2014b).	Moderate	The quality of evidence among studies that examined both TRAP and noise was moderate. [no additional rationale provided by review authors]	See Column F.	See Column F.	
Traffic- related air pollution	Reproductive outcomes	Noise			There was also limited evidence for small increases in adverse reproductive outcomes in relation to TRAP exposure after adjustment for noise, seen in three of five studies (Gehring et al., 2014; Nieuwenhuijsen et al., 2019; Smith et al., 2017b). In contrast, there was limited evidence of a lack of association between noise and adverse reproductive outcomes when TRAP exposures were also considered, as reported in three of five studies (Barba-Vasseur et al., 2017; Hjortebjerg et al., 2016; Nieuwenhuijsen et al., 2019).			See Column F.	See Column F.	***In this systematic review of the physical health impacts of four spatially covarying exposures related to urban form, 51 individual studies met pre-defined inclusion criteria. Following risk-of-bias assessments and strength-of-evidence analyses, evidence was inadequate across all health outcomes for the majority of the 11 possible combinations of the exposures (Fig. 2). Specifically, studies meeting inclusion criteria were only identified for five exposure groups. Of those, the majority included both TRAP and noise. When these exposures were considered jointly, evidence was sufficient for increased mortality with higher TRAP and limited for noise exposures; sufficient for increased cardiovascular morbidity with higher noise exposures and sufficient for no effect of TRAP on CVD morbidity; and limited for increased adverse reproductive outcomes with higher TRAP, but not noise, exposures. In addition, there was limited evidence for a direct association between higher levels of natural space exposure and reductions in chronic respiratory disease and adverse reproductive outcomes, as well for partial mitigation of the adverse effects of TRAP on chronic respiratory disease via increased exposure to natural spaces. Evidence was inadequate for all other outcomes and exposure groups. The potentially protective effects of higher natural space exposures on adverse reproductive health outcomes***
Traffic- related air pollution	Type 2 diabetes	Noise			Although there was inadequate evidence for effects of either TRAP or noise on Type 2 diabetes after adjustment for other exposures, results from two of the four studies that examined this outcome without a high risk of bias (Clark et al., 2012; Renzi et al., 2018) suggest the potential for an independent association between TRAP and a slightly higher risk of Type 2 diabetes when noise exposures are also considered.			See Column F.	See Column F.	
Traffic- related air pollution	Chronic respiratory disease	Natural spaces		Individuals of all ages residing in largely urban areas	Looking only at those studies without a high risk of bias and for which the relationship between natural spaces and chronic respiratory disease was adjusted for TRAP, there was limited evidence of a slightly lower risk associated with higher levels of exposure, as reported in three of five studies (Alcock et al., 2017; Fisher et al., 2016; Sbihi et al., 2015). The small number of studies that reported specifically on TRAP as a primary explanatory variable resulted in an score of inadequate, but two studies reported some evidence of partial mitigation of adverse TRAP effects by exposure to natural spaces (Alcock et al., 2017; Sbihi et al., 2015).		The quality rating for the set of studies examining TRAP and natural spaces was appraised as being low due to a high risk of bias and indirectness. Forty percent of the related studies have a high risk of bias (versus just 15% of	See Column F.	See Column F.	

Traffic- related air pollution	Physical health	Natural spaces	and bluespaces, defined as any area containing water, such as lakes, rivers, beaches, and decorative fountains. Finally, neighborhood walkability was defined as those aspects of the urban form that are known to impact pedestrian behavior, comprising the design and connectivity of street networks; the density of residential and retail properties; and land-use mix, or the range of destination types within an area (Frank et al., 2010).	The evidence was rated as inadequate for all other outcomes. In addition, the overall evidence base for the relationship between TRAP, natural spaces, and physical health was appraised as low quality due to the fact that most studies had either a moderate or high risk of bias and due to a serious risk of indirectness in light of the heterogeneity of their natural space exposure measures. Eight of the 20 included studies had a high risk of bias (Dadvand et al., 2014; Fisher et al., 2016; Lee et al., 2018; Oliveira et al., 2016; Shen and Lung, 2016, 2017; Skarkova et al., 2015; Tarocco et al., 2011), and six are ecological in design (Alcock et al., 2017; Oliveira et al., 2016; Shen and Lung, 2016, 2017; Skarkova et al., 2015; Tarocco et al., 2011). In addition, although the majority of studies include some form of nitrogen oxides as a marker for TRAP exposure, a wide range of other pollutants are included as well, and there is even larger heterogeneity in terms of natural space exposure. The range of outcomes is also large, leading to many outcomes with just a few related studies.	Low	those looking at TRAP and noise, as one comparison), and close to a third are ecological in design. In addition, although three-quarters of the studies include some form of nitrogen oxides as a marker for TRAP exposure, a wide range of other pollutants are also incorporated, and there is even greater heterogeneity in terms of natural space exposure measures and approaches.	See Column F.	See Column F.	***also persisted after mutual adjustment for noise or both TRAP and noise. These results indicate a need for epidemiologic studies focused on urban exposures to incorporate assessment of multiple exposures in their designs, particularly by adopting an exposome approach to capture the full swathe of correlated factors that may influence health across the lifecourse. Furthermore, consideration of policies related to the design and function of the built environment should be informed by such evidence to consider the impacts of multiple exposures.***
Traffic- related air pollution	Reproductive outcomes	Natural spaces, noise		There is limited evidence for the protective effects of natural space exposure on adverse reproductive outcomes after adjustment for these two co-exposures (Cusack et al., 2018; Hystad et al., 2014; Nieuwenhuijsen et al., 2019).			See Column F.	See Column F.	
Traffic- related air pollution	Mortality	Natural spaces, noise			Moderate		See Column F.	See Column F.	
Traffic- related air pollution	Cardiovascular disease	Natural spaces, noise		Due to the small number of studies with a minimal or moderate risk of bias examining other conditions – one for mortality (Vienneau et al., 2017), two for cardiovascular disease (Vivanco-Hidalgo et al., 2018, 2019), and two for diabetes (Clark et al., 2017; Renzi et al., 2018) – there is inadequate evidence for all other outcomes.		No rationale provided.	See Column F.	See Column F.	
Traffic- related air pollution	Diabetes	Natural spaces, noise					See Column F.	See Column F.	
Traffic- related air pollution	Health	Noise, natural spaces, walkability		With a total of only three studies (Clark et al., 2017; Hystad et al., 2014; Nieuwenhuijsen et al., 2019) that integrate all four of the spatially correlated urban exposures that are the focus of this review, there is insufficient evidence for either an overall quality rating or a strength of evidence summary score other than inadequate.	Moderate		See Column F.	See Column F.	

Table S9. Detailed Review Results

Exposure	Outcome	Non-Chemical Stressor	Non-Chemical Stressor Measurement/Definition	Population	Results Reported in Review	Certainty in the Body of Evidence	Certainty in the Body of Evidence Rationale	Author's Conclusions	Contributing Primary Studies	Overall Review Conclusion
					**Lin et al. 2022**					
PM2.5	Obesity	Country development status	Studies from "developed" countries vs studies from "developing" countries	Population level data	The results of both subgroups are statistically significant, and particulate matter in developing countries (RR: 1.172, 95% CI: 1.118–1.228, P <0.001) is more inclined to cause obesity than in developed countries (RR: 1.104, 95% CI: 1.037–1.175, P = 0.002). There is a low heterogeneity in the developed country subgroup, but still a considerable heterogeneity in the developing country subgroup.	Low	Since the studies we included are all observational studies, the initial level of certainty is low certainty, but these studies can improve the GRADE by meeting the upgrade criteria. The results showed that 4 studies are of a high certainty of the evidence, 8 studies are of moderate certainty of the evidence, 6 studies are of low certainty of the evidence, and 8 studies are of very low certainty of evidence. It can be seen that the certainty of the evidence is overall low after evaluation, and the reliability of our conclusions needs to be viewed dialectically and objectively.	Besides, no matter what the type of the PM, PM has a stronger effect on developing countries than developed countries.	Developed countries (n=6)<br>de Bonta 2019; Furlong et al. 2020; Wilding et al. 2020; de Bonta et al. 2021; Zhang et al. 2019; Bowe et al. 2021<br><br>Developing countries (n=13; all from China)<br>Zhang et al. 2019; Yang et al. 2019b; Guo et al. 2020; Liu et al. 2020; Cao et al. 2021; Yang et al. 2019c; Tu et al. 2021; Zhang et al. 2021; Zhang et al. 2021; Zheng et al. 2021; Huang et al. 2021; Liu et al. 2021<br><br>Europe (n=4)<br>de Bonta 2019; Furlong et al. 2020; Wilding et al. 2020; de Bonta et al. 2021<br><br>Asia (n=11)<br>Zhang et al. 2019; Yang et al. 2019b; Dong et al. 2014; Li et al. 2015; Liu et al. 2020; Yang et al. 2019c; Tu et al. 2021; Zhang et al. 2021; Zhang et al. 2021; Zheng et al. 2021; Liu et al. 2021	***In summary, exposure to PM2.5 (RR: 1.159, 95% CI: 1.111–1.209) and PM10 (RR: 1.092, 95% CI: 1.070–1.116) with per 10 μg/m3 increment could increase the risk of obesity in the global population. Our study reported that the associations between PM and obesity tended to vary by region, age group, and participants number, etc.***
PM10	Obesity	Country development status	Studies from "developed" countries vs studies from "developing" countries	Population level data	The PM10 in developed countries has no significant difference (RR: 1.100, 95% CI:0.990–1.223, P = 0.077), while increased the risk of obesity in developing countries (RR: 1.093, 95% CI: 1.067–1.119, P < 0.001). And there is huge heterogeneity in both subgroups.					
					**Yu et al., 2022**					
Air pollution	Autism spectrum disorder	Education	Maternal education (less than high school vs high school vs higher than high school)<br><br>Maternal education (<12 years of education vs >12 years)<br><br>Census tracts below the average percent of college education vs census tracts above the average percent of college education	Children	Becerra et al. (2013) found that the effect of traffic-related NO2 during pregnancy on ASD was statistically significantly higher in children whose mothers did not graduate from high school than in children whose mothers graduated high school. However, in the same study, stronger associations of CO and NO2 exposure during pregnancy were observed in children with maternal education level greater than high school compared with those with less than high school, and these differences in point estimates were statistically significant. A Swedish study reported that associations of traffic-related NOx and PM10 exposure during pregnancy and the first year of life were stronger in the more educated subgroup (with more than 12 years of education), but neither main effects nor interactions were statistically significant (Gong et al., 2017). An ecological study on air toxics found that the association of mercury with ASD risk was significantly higher in census tracts with below-average percentage of college educated households (Dickerson et al., 2016).	n/a	n/a	Three studies (2 case-control and 1 ecological design) demonstrated no consistent pattern of effect modification of the air pollution-ASD association by education level.	Becerra et al. 2013<br>Gong et al. 2017<br>Dickerson et al. 2016	
Air pollution	Autism spectrum disorder	Neighborhood characteristics	Neighborhood deprivation, census tract urbanicity, census tract poverty rate	Children	Two case-control studies examined the modifying effect of neighborhood deprivation, based on an index including income, education, unemployment and other socioeconomic characteristics in small socioeconomically homogeneous areas (Table 3). One U.S. study found that children living in the most deprived neighborhoods had stronger ASD associations with PM2.5 (dichotomized at 12 μg/m3) during the first year of life [OR = 2.17 (95%CI: 1.14, 4.15); interaction p-value 0.08] (McGuinn et al., 2019). When modeling PM2.5 as a continuous variable, the interaction between neighborhood deprivation level also was not significant in the same study (Table 3). In contrast, a Swedish study found that traffic-related NOx and PM10 exposure during the first year of life were more strongly associated with ASD in less deprived neighborhoods (p-interaction = 0.09) (Gong et al., 2017). Kalkbrenner et al. (2010) reported that there were no modifying effects of census tract urbanicity (100% rural, mixed, and 100% urban) on the association of air toxics with ASD. Dickerson et al. (2016) found that children from census tracts with higher poverty rates had stronger associations of a combined metals index with ASD.	n/a	n/a	Results from studies of effects of neighborhood deprivation on air pollution-ASD associations were inconsistent.	McGuinn et al. 2019<br>Gong et al. 2017<br>Kalkbrenner et al. 2010<br>Dickerson et al. 2016	***Sparse and inconsistent findings limit our ability to draw conclusions about how SES modifies air pollution effects. Further research is needed to identify susceptible populations and potential targets for preventive intervention.***
Air pollution	Autism spectrum disorder	Race	Not defined	Children	Racial differences in effects were examined in only one ecological study, in which there was an association of ASD with unhealthy air quality (AQI >100) only among Asians, which was not significant (OR = 3.59; 95%CI: 0.87, 14.8; p-value = 0.07) after adjustment for confounders (Al-Hamdan et al., 2018).	n/a	n/a	None	Al-Hamdan et al., 2018	
					**Deziel et al., 2020**					
Proximity to oil and gas activity	Pulmonary artery and valve defects; aortic artery and valve defects; Conotruncal defects; Tricuspid valve defects	Urban/Rural	rural urban zip codes	Children	Table 1. McKenzie et al (2019) Rural: Any CHD ↑ (OR: 2.47, 95% CI: 1.3, 4.4) Aortic artery and valve defects ↗ (OR: 2.67, 95% CI: 1.1, 6.1) Pulmonary artery and valve defects ↗ (OR: 1.57, 95% CI: 0.62, 3.9) Conotruncal defects ↑ (OR: 4.07, 95% CI: 1.4, 12) Tricuspid valve defects-less than 5 cases Urban: Any CHD ↘ (OR: 0.947, 95% CI: 0.47, 1.9) Aortic artery and valve defects ↘ (OR: 0.737, 95% CI: 0.23, 2.3) Pulmonary artery and valve defects ↗ (OR: 1.77, 95% CI: 0.59, 4.7) Conotruncal defects ↘ (OR: 0.917, 95% CI: 0.30, 2.8) Tricuspid valve defects - less than 5 cases	n/a	n/a	None	McKienzie et al. 2019	
Proximity to oil and gas activity	Infant mortalities	Private water and/or environmetnal law violations	county ratio of water wells divided by the number of births.	Infants	Busby and Mangano,(2017) reported that there is some evidence that the associations observed for infant mortality at the county level were related to private water well density and/ or environmental law violations.	n/a	n/a	None	Busby and Mangano, 2017	***Our review found that 25 of the 29 studies reported at least one statistically significant association between the UOG exposure metric***

Proximity to oil and gas activity	Antenatal anxiety and depression	Family income	Receipt of medical assistance	Women of reproductive age	Casey et al. (2019) reported that living in the highest quartile of conventional and unconventional oil and gas activity versus quartiles 1–3 would increase the incidence of antenatal anxiety or depression (4.3 additional cases per 100 women, 95% CI: 1.5, 7.0). This risk difference appeared larger among mothers receiving Medical Assistance, an indicator of low family income (5.6 additional cases per 100 women, 95% CI: 0.5, 10.6). This study is the first study that evaluated psychological outcomes based on clinical diagnosis and the results are coherent with the associations reported for the general population in the self-reported studies.	n/a	n/a	None	Casey et al. 2019	***and an adverse health outcome. The most commonly studied endpoint was adverse birth outcomes, particularly preterm deliveries and low birth weight. Few studies evaluated the mediating pathways that may underpin these associations, highlighting a clear need for research on the potential exposure pathways and mechanisms underlying observed relationships.***
**Payne Sturges et al., 2023**										
Ambient air pollution	Neurodevelopmental outcomes	SES		Children in the US	Nineteen (or 37% of 52) of the ambient AP studies included an investigation of effect modification, 3 (or 6%) examined interaction and 3 studies (or 6%) presented results for both (Table 2). The impacts of AP exposures on juvenile delinquent behavior was stronger in families with psychosocial adversities, associated with worse scores on the Wide Range Assessment of Memory and Learning, 2nd ed.assessment (WRAML2), among Hispanic and Black boys with exposure to high prenatal stress, associated with more adverse Performance IQ scores among children from families with low SES and had stronger association with ASD for those living in high-rather than moderate-or low-deprivation neighborhoods.<br><br>SES status was a significant modifier for fine particle exposures and lower IQs, and this was stronger with the Performance IQ results. However, formal tests of heterogeneity were not performed by these authors for any of these studies. Investigations of effect modification with formal tests included findings that neighborhood SES was a significant modifier for effects of exposures to criteria pollutants on NTD, early life stress magnified the psychiatric effects of PAH exposures, and neighborhood low English proficiency worsened cognitive test scores within the low PAH exposure stratum. Of the 17 studies (29%) of ETS exposure investigated efect modifcation or interactions. All 5 studies reported interactions or effect modification between ETS and measures of health disparity, including poverty, race, and material hardship, on neurodevelopmental outcomes that included learning disabilities, cognitive de cits, and ADHD.	n/a	n/a	None	Wang et al. 2017<br>McGuinn et al. 2019<br>Padula et al. 2017<br>Pagliaccio et al. 2020<br>Lovasi et al. 2014	
Lead	Neurodevelopmental outcomes	SES	SES was measured in a variety of ways (income, parental education, poverty status, insurance) at the individual, family, and area level. Papers often used more than one SES variable in their analyses. Measurement of SES variables was less likely to be "unclear or not stated" as compared with race and ethnicity variables.	Children in the US	Fifteen studies examined SES. Nine studies revealed more adverse impact of Pb exposure in children from families with low SES. For example, in the Cincinnati Lead Study (CLS) with enrollment from 1979–1984, infant BBL sat 10 dofage (mean=4:6 y,range:1–22 lg=dL) had an interaction with SES(p=0:01) on Bayley Mental Development Index (MDI), indicating a deficit of 16.1 points in MDI across this range of exposure in infants from families with below-median SES. This interaction remained at 4 years of age when the children were assessed by Kaufman Assessment Battery for Children (K-ABC). Neonatal BBLs were inversely associated with K-ABC Mental Processing Composite (MPC) in children from families with below-median SES but not in children from families with higher SES. Subsequently, this research group's studies showed that BBLs at 78 months of age were associated with deficits in learning/ IQ in mid adolescence(p<0:07) for children from families with low SES.	n/a	n/a	None	Bellinger et al. 1988<br>Bellinger et al. 1990<br>Dietrich et al. 1987<br>Dietrich et al. 1991<br>Coscia et al. 2003<br>Greene and Ernhart 1993<br>Ji et al. 2018<br>Magzamen et al. 2013<br>Magzamen et al. 2015<br>Marshall et al. 2020<br>Mendelsohn et al. 1998<br>Mendelsohn et al. 1999<br>Miranda et al. 2009<br>Needleman et al. 2002<br>Ris et al. 2004	
Mercury	Neurodevelopmental outcomes	SES		Children in the US	Only two investigations considered interactions by comparators (Table 4). Brender et al. reported that household income modified the association between urinary Hg and NTD, whereas Orenstein et al. found no significant interactions with the socio- demographic variables. Specifically, Brender et al. noted that among women with the highest income (>$25,000), women with children born with neural tube disorder were nine times more likely (OR=9:0;95%CI:1.4,57) than control- women to have a urinary Hg level of ≥5:62 lg=L,a level that represented the 95th percentile for Mexican-American participants in the 1999–2000 NHANES. Quantitative results were not provided by Orenstein et al.	n/a	n/a	None	Brender et al. 2006<br>Orenstein et al. 2014	
Organophosphate pesticides (Ops)	Neurodevelopmental outcomes	SES		Children in the US	One study reported significant effect modification by financial hardship of the association between OP exposure and ASD.<br>Two studies found no significant interactions of prenatal chlorpyrifos exposure with neighborhood poverty.<br>Although statistically significant interaction between proximity to pesticide use and neighborhood poverty was not observed, Rowe et al. noted that the results of the multivariable regression models stratified by household poverty suggest that 10-y-old children in poorer households may experience greater cognitive impacts in association with OP and carbamate exposures. Statistically significant associations were found between living in the highest quartile of proximal pesticide use and full-scale IQ (FSIQ; β=−3.8; 95%CI=−7.0,−0.5), Perceptual Reasoning (β=−4.4; 95%CI=−8.7,−0.1), and Working Memory (β=−3.8; 95% CI: −7.2,−0.5) among children in households at or below the poverty threshold but not in those in households above the poverty threshold.	n/a	n/a	None	Lovasi et al. 2014<br>Taiwo 2019<br>Rowe et al. 2016	
Polychlorinated biphenyls (PCBs)	Neurodevelopmental outcomes	Sociodemographic variables		Children in the US	Only one study assessed interaction (Table 7): Orenstein et al. found no significant interactions with PCBs and the sociodemographic variables (i.e., maternal IQ, prenatal smoking, parental education, household income, and breastfeeding). Perhaps this is not surprising given that the authors did not find significant associations between prenatal PCB exposures and memory and learning skills as assessed by the WRAML or other learning outcomes.	n/a	n/a	None	Orenstein et al.	

Multiple chemical exposures /mixtures	Autism spectrum disorder	SES	Census tracts above vs below the poverty line	Children in the US	Five of the 17 mixtures studies (29%) assessed for effect modification by or interaction with sociodemographic or socioeconomic comparator variables (Table 8). Only Furlong et al. and Dickerson et al. found evidence of effect modification. For example, in census tracts below the poverty level and with combined metal concentrations (Pb, Hg, and As) in the 50th to the 75th percentile, adjusted relative risk (RR) for ASD prevalence was 1.36 (95% CI: 1.06, 1.74) compared with those in the lowest quartile of exposures.145 Conversely, among tracts above the poverty level, the RR was 1.10 (95% CI: 0.57, 2.10) for the same exposure comparison.	n/a	n/a	None	Dickerson et al.	***As a whole, the studies we reviewed indicated a complex story about how racial and ethnic minority and low-income children may be disproportionately harmed by exposures to neurotoxicants, and this has implications for targeting interventions, policy change, and other necessary investments to eliminate these health disparities. We provide recommendations on improving environmental epidemiological studies on environmental health disparities. To achieve environmental justice and health equity, we recommend concomitant strategies to eradicate both neurotoxic chemical exposures and systems that perpetuate social inequities.***
Ambient air pollution	Neurodevelopmental outcomes	Race/ethnicity		Children in the US	The impacts of AP exposures was associated with worse scores on the Wide Range Assessment of Memory and Learning, 2nd ed. assessment (WRAML2), among Hispanic and Black boys with exposure to high prenatal stress. Confidence in these associations were bolstered with statistically significant interaction terms reported by authors.<br><br>Other studies that evaluated interactions between PAH exposures, as measured by cord PAH-DNA adducts, and comparator variables found that joint exposure with material hardship resulted in reduced IQ and multiple ADHD symptom scores (more symptoms), whereas there was no evidence of interaction between AP and ethnicity (defined by authors as Black and Dominican) impacting IQ.<br><br>Among the studies with analyses on effect modification, all but two reported significant differences in effects by race, ethnicity, or SES. One of the few studies that included Asian populations, Al-Hamdan etal., reported effect modification by race/ethnicity was significant only for Asian populations exposed to unhealthy air quality [as measured by air quality index (AQI)] for ASD outcomes [odds ratio ðORÞ=2:96; 95% confidence interval (CI):1.11,7.88] as opposed to Hispanic populations (OR=1:308; 95%CI:0.607, 2.820), Black populations (OR=1:398; 95%CI:0.827,2.364), and White populations (OR=1:219; 95%CI:0.760,1.954). Maternal age and race/ethnicity was a significant modifier for proximity to Toxic Release Inventory (TRI) sites with chemical emissions and NTDs, but was significant only for mothers>35 years of age and for non-Hispanic white mothers.	n/a	n/a	None	Cowell et al. 2015<br>Perera et al. 2009<br>Al-Hamdan et al. 2018<br>Suarez et al. 2007	
Lead	Neurodevelopmental outcomes	Race/ethnicity	Among the 165 articles that included race and/or ethnicity variables, a majority (151 of 165 articles or 92%) did not provide a definition or a conceptualization. Among the few that did, the reasons varied from "because other studies included race" and race "as a distinct human type based on inherited physical characteristics" to race as culture with influence on diet and "behaviors and patterns of consumption." Race and ethnicity were sometimes conflated and together considered as indicators of culture. One article explicitly considered race as a proxy for institutional racism and psychosocial stress and another stated that race was a marker for "diminished life chances."	Children in the US	Of the 11 studies that examined effect modification or interaction by race, 5 studies (or 45%) reported worse out comes among racially minoritized children.Two of these studies found interactions between BBLs and race on school performance, using Pb screening data from early life and standardized test scores at school age. In Chicago Public School children,an interaction between race and BBLs was identified for reading and math test failure in the third grade. The OR of reading test failure per 5-lg=dL increase in BBLs was 1.28 (95%CI:1.21,1.35) in non-Hispanic black children, 1.47 (95%CI:1.29,1.66) in Hispanic children, and 1.93 (95%CI:1.47, 2.54) in non-Hispanic white children.70 However, because non-Hispanic Black children had the highest mean BBLs, the combined racial disparities for both exposure and outcome are greater than those ORs indicate. Lu et al. tested racial disparities in the effects of Pb in community drinking water supplies by adding an interaction term to their model and reported "effect measure modification by grade is statistically significant for math [b= −0:0242, standard error ðSEÞ=0:0051, p<0:01]."125 In other words, for student populations with a higher proportion of non-White students in Massachusetts school districts, "higher drinking water lead levels were associated with a larger reduction in standardized math test scores, compared with cohort years with more than 90% White students."	n/a	n/a	None	Amato et al. 2013<br>Coscia et al. 2003<br>Evens et al. 2015<br>Greene and Embart 1993<br>Lu et al. 2022<br>Magzamen et al. 2013<br>Magzamen et al. 2015<br>Mendelsohn et al. 1999<br>Min et al. 2009<br>Needleman et al. 2002<br>Wright et al. 2021	
Organophosphate pesticides (Ops)	Neurodevelopmental outcomes	Race/ethnicity		Children in the US	In one study from the Mount Sinai Children's Environmental Health Study, third trimester maternal urinary DAP metabolites were assessed for their association with scores on the Social Responsiveness Scale (SRS) for children 7–9 years of age.135 Although there was no overall association, for Black children, each 10-fold increase in total diethylphosphates (ΣDEP) metabolites was associated with poorer social responsiveness (β=5.1 points; 95% CI: 0.8, 9.4), as well as among boys in general (β=3.5 points; 95% CI: 0.2, 6.8).135 The association of ΣDEP metabolites with total SRS score was heterogeneous by race and by sex: ΣDEP p interaction=0.06 for race, and ΣDEP p interaction=0.12 for sex.135 However, to perform this analysis, Furlong et al. had to combine White and Hispanic study participants for sample size concerns. In a pooled analysis across four cohorts (from California, New York, and Ohio), Engel et al. reported that there was significant heterogeneity in the ΣDAP and total dimethylphosphates (ΣDMP) associations with the MDI at 24 months of age by race and ethnicity (p=0.06 and p=0.02, respectively), with the strongest negative associations found among Hispanic participants for ΣDAP and ΣDMP (ΣDAP β=–2.91; 95% CI: –4.71, –1.12; ΣDMP β=–2.34; 95% CI: –3.77, –0.91).92 Authors reported that the overall pooled association was still negative (ΣDAP β=–1.39; 95% CI: –2.67, –0.10) and concluded this was to a large degree driven by the strong negative association among Hispanic participants, specifically from the CHAMACOS cohort in California which "accounted for approximately 70% of all Hispanics included in this pooled analysis."	n/a	n/a	None	Furlong et al. 2014<br>Engel et al. 2016<br>Engel et al. 2011<br>Percy et al. 2022<br>Rauh et al. 2006<br>Rauh et al. 2011	
Pthalates	Neurodevelopmental outcomes	Race		Children in the US	Only one study assessed interaction. Bloom et al. used cross-product terms for race×Phth×time in regression models and found reduced head circumference associations with prenatal Phth exposures were stronger among White mothers than among African American mothers. It was unclear if these interactions were on the additive or multiplicative scale.	n/a	n/a	None	Bloom et al. 2021	

Multple chemical exposures/ mixtures	Neurodevelopmental outcomes	Race	Children in the US	When stratifying results by race, Furlong et al. noted that the association between the sum of dimethylphosphate metabolites (ΣDMPs) and decrements in the Working Memory Index differed by race/ethnicity, with a negative or inverse association among Black participants and no associations among White or Hispanic participants when accounting for other chemical exposures (9 Phth, 3 pyrethroid, and 5 phenol metabolites modeled as coexposures). This is counterintuitive because it suggests that pesticide exposures lead to better neurodevelopmental outcomes among Black children. The authors stated that this association between ΣDMPs and executive functioning among Black participants, but not Hispanic or White participants, was unexpected and may reflect residual confounding by race-specific factors or may be a chance finding given the small sample size. Neither Dickerson et al. or Furlong et al. mentioned a formal test of heterogeneity. In addition, Furlong et al. often used the terms effect modification and interaction interchangeably, and their “Methods” section did not provide enough information as to whether they also used interaction terms.<br><br>The studies by Dellefratte et al. and Persico et al. did not find a statistically significant difference in the exposure–effect relationships by poverty (air toxics and ADHD) or by race (proximity to TRI facilities and Superfund sites and cognitive outcomes) or statistically significant interaction terms. Although Persico et al. did not find differences in exposure–effect relationships by race (i.e., children from all backgrounds are harmed by proximity to Superfund sites before and during cleanup), these authors estimated that the racial disparities in exposures to environmental toxicants from Superfund sites alone accounted for ~2% of the Black–White cognitive test score gap in Florida during their study period.	n/a	n/a	None	Furlong et al.<br>Persico et al.

Supplement-10 Deviations from the Protocol

- We modified our search, limiting the number of databases. We did not search grey literature.
- During study screening, we ensured 100% agreement for all included studies, rather than for a random sample of 5% of screened studies.
- We refined the exposure of the PECO statement to include indicators for: allostatic load, discrimination/racism, education, food insecurity, geography, protective factors, socioeconomic status (SES) or socioeconomic position (SEP), sleep quality, general stress general and work-related stress. This was done prior to data extraction and evaluation.
- We added additional data extraction fields, including exposure measurement, certainty of the body of evidence ratings and rationale, and author's conclusions. We did not extract primary study sample sizes.
- We amended our methods for managing primary study overlap. While our protocol notes that when overlap is identified, we would only present the highest quality reviews, those with more recent publication dates, and/or those with meta-analyses, we decided to present the results of all reviews regardless of overlap. Cochrane's Overview of Reviews guidance states that "if the purpose is to present and describe the current body of systematic review evidence on a topic, it may be appropriate to include the results of all relevant systematic reviews, regardless of topic overlap" (27).
- The study protocol did not describe our methods for identifying study sponsorship and author conflict of interest. Our methods were established prior to data extraction and can be found in a footnote to Table 1.
